# Design, Synthesis and Biological Evaluation of Novel Thienylpyridyl- and Thioether-Containing Acetamides and Their Derivatives as Pesticidal Agents

**DOI:** 10.3390/molecules26185649

**Published:** 2021-09-17

**Authors:** Huan Li, Na Yang, Lixia Xiong, Baolei Wang

**Affiliations:** State Key Laboratory of Elemento-Organic Chemistry, College of Chemistry, Nankai University, Tianjin 300071, China; 2120180790@mail.nankai.edu.cn (H.L.); flyna2010@nankai.edu.cn (N.Y.); xionglixia@nankai.edu.cn (L.X.)

**Keywords:** thienylpyridylthioacetamide, thioether, sulfone, synthesis, pesticidal activity

## Abstract

Referring to the structural information of the “hit” compound **A** from the reported pharmacophore-based virtual screening, a series of novel thienylpyridyl- and thioether/sulfoxide/sulfone-containing acetamide derivatives have been designed and synthesized. The structures of new compounds were confirmed by ^1^H NMR, ^13^C NMR and HRMS. The single-crystal structure of **A** was firstly reported. All the new synthesized compounds were evaluated for insecticidal activities on *Mythimna separata* Walker and *Plutella xylostella* L. Through a step-by-step structural optimization, the high insecticidal agents, especially towards *Plutella xylostella* L., have been found, and thienylpyridyl- and sulfone/thioether-containing acetamides **Iq**, **Io**, **Ib** and **A,** which are comparable with the control insecticides cartap, triflumuron and chlorantraniliprole in the present study, can be used as novel lead structures for new insecticides innovation research. In addition, some of the compounds, e.g., **A**, **Ih**, **Id**, **Io** and **Iq**, also exhibited favourable fungicidal activities against *Physalospora piricola*, *Rhizoctonia cerealis* and *Sclerotinia sclerotiorum* and would provide useful guidance for the design and development of new fungicides.

## 1. Introduction

**Figure 1:**
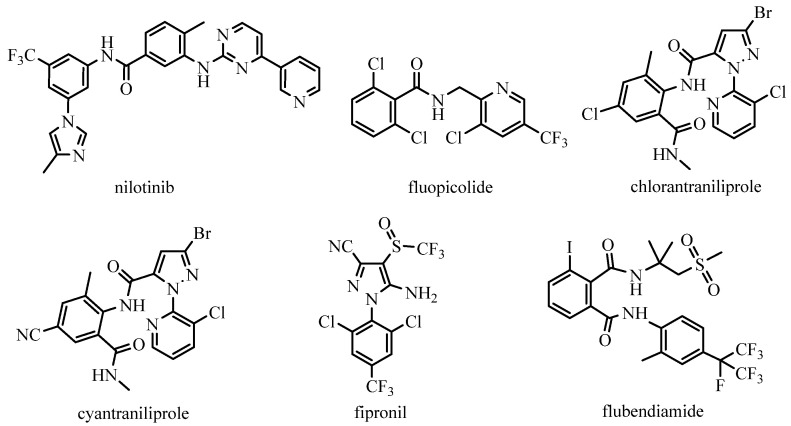
The chemical structures of some bioactive agents containing pyridine, amide and sulfur groups.

The discovery and development for new agrochemicals with novel structures, excellent biological activities and other beneficial properties are of great significance to agriculture and are also continuous subjects for pesticide researchers [1,2,3,4]. It is known that compounds containing amide or heterocycle moiety have been extensively investigated owing to their versatile biological activities in medicinal chemistry and pesticidal chemistry for long years. Amides and heterocycles are often important pharmacophores in many agrochemicals, either alone or in combination. As the typical representatives of heterocyclic compounds, pyridine derivatives exhibit a broad range of bioactivity potentials, and many of them have been developed as pharmaceuticals, insecticides, herbicides, fungicides, etc. [5,6,7,8]. For example, as shown in Figure 1, nilotinib is a kind of anticancer agents that has a pyridine ring [9]; chlorantraniliprole and cyantraniliprole, DuPont’s famous insecticides targeting at the ryanodine receptor (RyR), are both amide compounds with pyridine heterocyclic motif [10,11]. Similarly, organic sulphur molecules are also an important part of bioactive compounds. Thioethers are one type of organic sulphur compound that not only hold a variety of biological activities but can also provide reaction sites for further derivatizations (such as S to S=O and S(=O)_2_) and for the environmental degradation [12]; for example, fipronil and flubendiamide (Figure 1) are potent insecticides containing sulfoxide and sulfone motif, respectively, [13,14], and both groups are derived from their thioether precursors.

**Figure 2:**
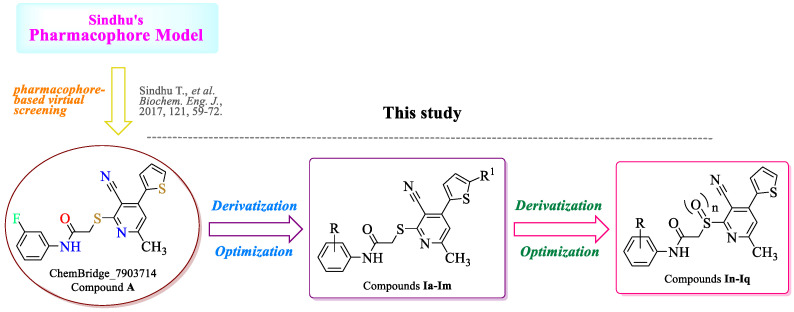
The design of the target compounds.

Recently, Sindhu et al. carried out a pharmacophore-based virtual screening study to look for potential insect RyR modulators that could offer useful information for insecticide design. Some top-ranked molecules via such research were obtained from a virtual screening of 520,000 compounds in the ChemBridge chemical database using the pharmacophore model they developed [15]. Regrettably, Sindhu et al. did not conduct a further insecticidal bioassay for those “hit” molecules, that is, the experimental validation of their molecular design results from the pharmacophore-based virtual screening. Enlightened by this, one of the “hit” molecules, 2-((3-cyano-6-methyl-4-(thiophen-2-yl)pyridin-2-yl)thio)-*N*-(3-fluorophenyl)acetamide (code-name: ChemBridge_7903714) (compound **A** in Figure 2), aroused our research interest. For one thing, compound **A** contains structural characteristics of substituted thienyl-pyridine heterocycles, thioether and acetamide moieties that are all important bioactive groups in many agrochemicals as mentioned above; a combination of them and further optimization or derivatization may lead to new discoveries. For another, there are several studies related to pyridylthioether-containing acetamides been reported; however, they mainly focus on the synthesis and the pharmaceutical applications, such as anti-influenza agents [16], anti-HIV agents [17] and antibacterial agents [18]; it is worth noting that this kind of structure has never been reported to have pesticidal activities. In addition, to our best knowledge, the synthetic procedure and the physicochemical and biological properties of compound **A** have not yet been reported. Therefore, compound **A** was selected as a reference structure to carry out the synthesis, pesticidal evaluations and the structure–activity relationship investigations of itself and some newly designed/derivative compounds in this paper.

## 2. Results and Discussion

### 2.1. Chemistry

**Scheme 1:**
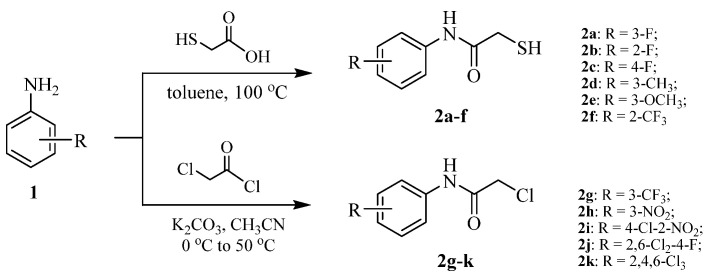
Synthesis of the intermediates **2a–f** and **2g–k**.

**Scheme 2:**
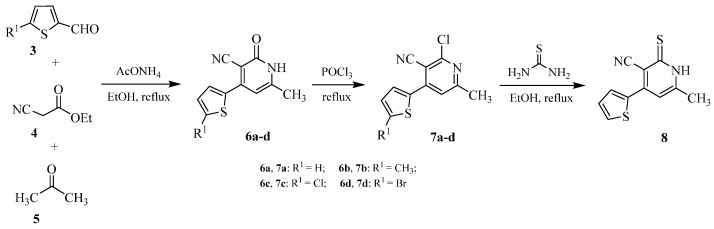
Synthesis of the intermediates **6a–d**, **7a–d** and **8**.

According to the structural feature of the target molecules designed, there are two strategies to generate the pyridylthioacetamides―the nucleophilic substitution of pyridine halide with mercaptoacetamide or pyridine thiol with chloroacetamide. Both methods for preparing the similar skeleton have been proven to be effective under alkali promotion [16,19]. In view of the possible influence of different substituents in various involved intermediates on the reactivity, intermediates 2-mercapto-*N*-arylacetamide **2a**–**f** and 2-chloro-*N*-arylacetamide **2g**–**k**, were prepared using their appropriate method, respectively, from the starting material of substituted aniline **1** with satisfying yields (Scheme 1). Correspondingly, as their counterparts for the nucleophilic substitution, intermediates 2-chloro-4-(5-chlorothiophen-2-yl)-6-methylnicotinonitrile (**7**) and 6-methyl-4-(thiophen-2-yl)-2-thioxo-1,2-dihydropyridine-3-carbonitrile (**8**) were prepared, as shown in Scheme 2, in which the latter (**8**) was generated through a thiourea-based sulfhydrylation of the former (pyridine chloride **7**, R^1^ = H) in 88% yield. Compounds **7** were derived from the POCl_3_-chlorination of thienyl-containing pyridinone derivatives (**6**), which can be very efficiently obtained via a similar four-component reaction of 5-substitutedthiophene-2-carbaldehyde (**3**), ethyl 2-cyanoacetate (**4**), acetone (**5**) and ammonium acetate in refluxing ethanol, as reported by Elsaman et al. [20].

**Scheme 3:**
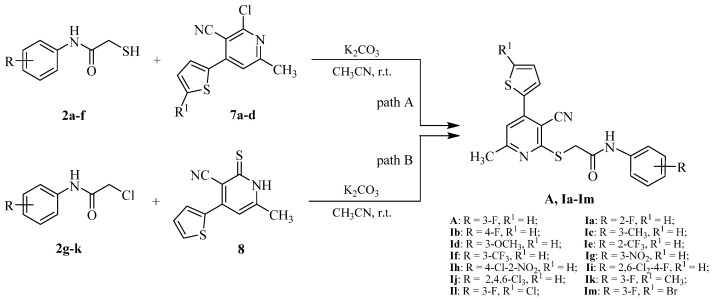
Synthesis of the title compounds **A** and **Ia–Im**.

As shown in Scheme 3, both the nucleophilic substitution reactions of mercaptoacetamide (**2a–f**) with pyridine halide (**7a–d**) and chloroacetamide (**2g–k**) with pyridine thiol (**8**) (i.e., by path A and path B, respectively, in Scheme 3) under conditions of K_2_CO_3_/CH_3_CN and room temperature successfully gave rise to the target compounds **A** and **Ia–Im** in good yields.

**Scheme 4:**
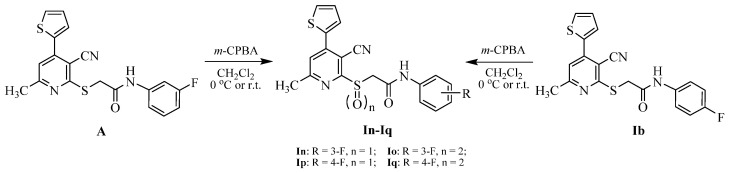
Synthesis of the title compounds **In–Iq**.

For the synthesis of the derivatives **In–Iq** from their thioether precursors **A** and **Ib**, initially, hydrogen peroxide (30%) was used as a common oxidant [21] to conduct such an oxidation reaction. Regrettably, it was found that some oxidation systems, including H_2_O_2_/CH_2_Cl_2_, H_2_O_2_/AcOH and H_2_O_2_/CF_3_CO_2_H, at room temperature could not apparently afford the desired sulfone or sulfoxide derivatives in our experiments. Pleasingly, *m*-chloroperoxybenzoic acid (*m*-CPBA) was found to be effective in the subsequent attempts. As a result, by means of the reaction of compound **A** or **Ib** and *m*-CPBA in CH_2_Cl_2_ at 0 °C or room temperature, oxidation products, that is, the sulfoxide derivatives (**In**, **Ip**) and the sulfone derivatives (**Io**, **Iq**), were smoothly achieved, respectively, in good yields (Scheme 4).

The target compounds **A** and **Ia–Iq** were identified by melting points, ^1^H NMR and ^13^C NMR spectra, and the HRMS results also showed satisfactory consistency between the measured and calculated ones. In the ^1^H NMR of **A** and **Ia–Iq**, the active proton (NH) signal was observed at downfield with the chemical shift of 8.76–10.81 ppm due to the deshielding effects of its neighbouring carbonyl and phenyl groups. The CH_2_ proton signals between carbonyl and the sulphide group mostly appeared at a chemical shift of 3.98–4.85 ppm. Among them, the protons chemical shift (CH_2_) of **In–Iq** is larger than that of **A** and **Ia–Im** due to the stronger deshielding effect of the sulfoxide group and the sulfone group. For compounds **In** and **Ip**, the proton signals in this CH_2_ group appeared in “m” peaks, possibly derived from the influence of the neighbouring asymmetric sulphur group (S=O). In the ^13^C NMR of the target compounds, the carbon signals for the corresponding CH_2_ were observed at chemical shifts of 34.01–35.67 ppm for compound **A** and **Ia–Im** and 58.50–60.37 ppm for compounds **In–Iq**. In addition, the typical characteristic absorption peaks of carbon in the carbonyl group (C=O) and the cyano group (CN) for all the target compounds were found at chemical shifts of 162.31–167.85 ppm and 113.24–118.97 ppm, respectively.

**Figure 3:**
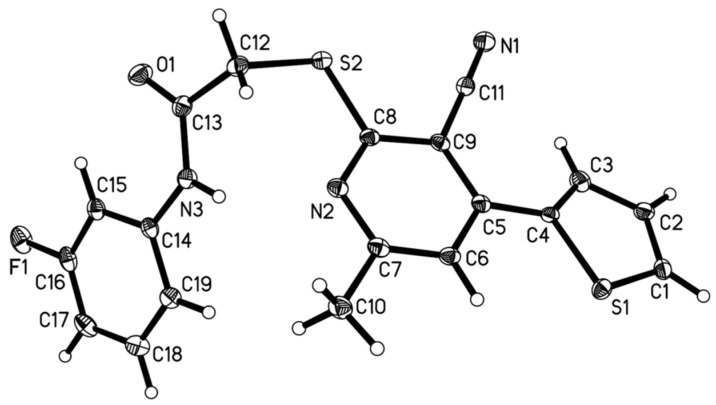
The single crystal structure of compound **A** (CCDC 2102104).

The structure of compound **A** (CCDC No.: 2102104) was further confirmed through single-crystal X-ray diffraction analysis. The Crystal Data of compound **A** for C_19_H_14_FN_3_OS_2_ (M = 383.45 g/mol): monoclinic, space group P2_1_/n, *a* = 13.8820(5) Å, *b* = 8.7554(3) Å, *c* = 14.5268(6) Å, *β* = 100.698(4)°, *V* = 1734.93(11) Å^3^, *Z* = 4, *T* = 113.15 K, *μ*(MoK*α*) = 0.331 mm^−1^, *D*_calc_ = 1.468 g/cm^3^, 14,637 reflections measured (3.728° ≤ 2θ ≤ 52.742°), 3517 unique (*R*_int_ = 0.0423, *R*_sigma_ = 0.0323), which were used in all calculations. The final *R*_1_ was 0.0345 (I > 2σ(I)), and *wR*_2_ was 0.0869 (all data). The molecular structure of **A** is shown in Figure 3. The crystallographic parameters and collected diffraction data are provided in the Supplementary Materials.

### 2.2. Biological Activities and Structure-Activity Relationship

#### 2.2.1. Insecticidal Activity

Initially, as the “hit” molecule of Sindhu’s research [15], compound **A** was successfully prepared in our lab, and we further evaluated the insecticidal effect for the first time. At a test concentration of 200 mg/L, **A** was found to have good larvicidal activity towards the oriental armyworm (*Mythimna separata* Walker) and diamondback moth (*Plutella xylostella* L.), with a lethality rate of 60% and 100% (100% means total kill), respectively (Table 1). Therefore, this indicates that Sindhu’s pharmacophore model was of rationality to a great degree under the validation of our experiments. On the other hand, this result also provided us with an available clue for further structural optimization according to this novel structure, which contains thienylpyridine heterocycle, thioether and acetamide moieties. Subsequently, compounds **Ia–Im** as new analogues of **A** were further synthesized for biological activity evaluation (Figure 2). Furthermore, based on the optimization result analysis of these compounds, several thioether compounds with the best insecticidal potential were further conducted for derivatization of transforming into sulfoxides and sulfones (compounds **In–Iq**).

From Table 1, it can be seen that most of the target compounds showed obvious insecticidal activity towards the oriental armyworm at a test concentration of 200 mg/L. Among which, compounds **A**, **Ik**, **Il**, **In** and **Ip** possessed a lethality rate of 50–70%. Interestingly, all the compounds exhibited good, even excellent, larvicidal activity (40–100%) against the diamondback moth at an initial concentration of 200 mg/L. When concentrations for the tested compounds were decreased to 100 mg/L and 10 mg/L, most of the compounds can still maintain good activity (>50%). Compounds **A**, **Ib**, **Ig**, **Io** and **Iq,** which had lethality rate of 50–100% at 1 mg/L, were found to be superior to that of the control insecticide cartap (40% at 10 mg/L) and equal to even higher than that of control insecticide triflumuron (50% at 1 mg/L). The insecticidal activity of compounds **Ib** (thioether, R = 4-F, R^1^ = H) and **Iq** (sulfone, R = 4-F, R^1^ = H, *n* = 2) at lower concentration of 0.1 mg/L can reach to 70% and 100%, respectively. In particular, compound **Iq** possessed a 43% lethality rate against diamondback moth at 0.001 mg/L, close to that of the best control chlorantraniliprole (83%) under the same test condition. As shown in Table 2, the further LC_50_ determination against the diamondback moth indicated that the best **Iq** in this series of compounds had an LC_50_ value of 0.0016 mg/L. Although less active than the commercial chlorantraniliprole (LC_50_ = 0.0001 mg/L), **Iq** was found to be more potent than triflumuron (LC_50_ = 0.3627 mg/L).

Through an analysis of the insecticidal data against the diamondback moth of the target compounds along with their structures, the following structure–activity relationship could be summarized. Firstly, a combination of thienylpyridine heterocycle, thioether and acetamide motif would generate a very high insecticidal effect. Secondly, for the target compounds **A** and **Ia–Im**, when the R^1^ group was fixed as H, the R group in the benzene ring of the corresponding compounds showed an activity trend: 4-F > 3-F > 3-NO_2_ > 3-OCH_3_ > 2-F > 4-Cl-2-NO_2_ > 3-CF_3_ > 2,6-Cl_2_-4-F > 2-CF_3_ > 3-CH_3_ > 2,4,6-Cl_3_; among them, mono-substituent compounds generally exhibited more favourable influence on the insecticidal activity than that of tri-substituent or di-substituent, while for the same mono-substituent at the *meta*-position, the F group (i.e., 3-F) was the best one (compound **A**); then the further investigation on the position effect based on compound **A** (R = 3-F) led to the activity sequence of 4-F > 3-F > 2-F. Thirdly, when R = 3-F, the activity trend for the R^1^ group was H > Cl > Br > CH_3_ (i.e., **A** > **Il** > **Im** > **Ik**). Therefore, it could be concluded that the optimal substituent combination in these thioether-type compounds (**A** and **Ia–Im**) for the insecticidal activity promotion was R = 4-F and R^1^ = H. In addition, the further S-derivatization for the best bioactive thioether compounds **A** and **Ib** gave rise to their sulfoxide and sulfone derivatives **In–Iq**, and the results exhibited the activity sequence of sulfone > thioether > sulfoxide and 4-F > 3-F for the R group (i.e., **Iq** > **Ib** > **Ip**; **Io** > **A** > **In**; **Iq** > **Io**).

**Figure 4:**
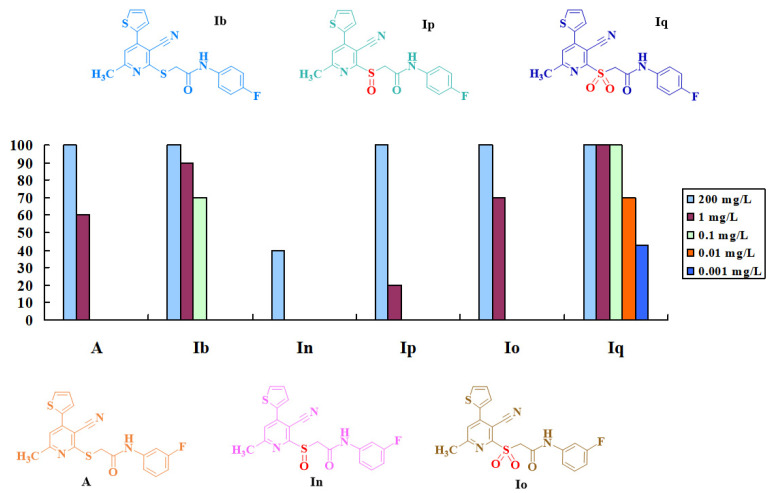
The larvicidal activity contrast diagram of compounds **A**, **Ib**, **In**, **Ip**, **Io** and **Iq** against diamondback moth (*Plutella xylostella* L.) at different test concentrations.

These results indicate that the high-insecticidal agents have been found through a step-by-step structural optimization based on Sindhu’s research and our further validation, design, synthesis and biological evaluations. The structure―activity trend for partial compounds could be well reflected from the illustration in Figure 4. Among the synthesized compounds, thienylpyridyl- and sulfone/thioether-containing acetamides **Iq**, **Io**, **Ib** and **A** can serve as novel lead structures for innovative research on new insecticides in the future.

#### 2.2.2. Fungicidal Activity

Taking into account their novel structures and the research gap of these kinds of structures in pesticide area, besides insecticidal activity, these synthesized compounds were also investigated for the fungicidal activity towards a variety of common agricultural pathogenic fungi, including *Sclerotinia sclerotiorum*, *Botrytis cinerea*, *Pellicularia sasakii*, *Fusarium oxysporum*, *Physalospora piricola* and *Rhizoctonia cerealis*. As shown in Table 3, most of the compounds exhibited apparent in vitro fungicidal activities at a test concentration of 50 μg/mL. Comparatively, these compounds had more favourable fungicidal activities against *Physalospora piricola*, *Rhizoctonia cerealis* and *Sclerotinia sclerotiorum* on the whole. For example, compounds **A** and **Ih** against *Rhizoctonia cerealis* held an inhibition rate of 69.2% and 67.7%, respectively; compounds **Ic**, **Id** and **Ip,** which possessed 71.1–81.6% activity towards *Physalospora piricola,* showed a better fungicidal effect than that of the control triadimefon (65.8%) and were close to that of chlorothalonil; compounds **A** and **Io–Iq** had a 60.7–91.1% inhibition rate against *Sclerotinia sclerotiorum*, and in particular, **Io** (91.1%) displayed an approximate fungicidal level similar to the control fungicides chlorothalonil (98.2%), azoxystrobin (92.9%) and triadimefon (98.2%).

Due to the novel structures and favourable fungicidal potentials, some of these compounds can provide useful guidance and reference for the design and development of new fungicides.

## 3. Experimental Section

### 3.1. General Information

The melting points were determined using an X-4 binocular microscope apparatus and were uncorrected. ^1^HNMR spectra and ^13^CNMR spectra were recorded at 400 MHz and 101 MHz, respectively, using a Bruker AV 400 spectrometer in CDCl_3_ or acetone-*d*_6_ or DMSO-*d*_6_ solvent with tetramethylsilane as the internal standard, and chemical shift values (*δ*) were given in parts per million (ppm). High-resolution mass spectrometry (HRMS) was determined on Varian QFT-ESI and Thermo Q Exactive Focus-ESI instruments. The single-crystal structure determination was conducted using a Rigaku Saturn 70 CCD diffractometer. All reagents and solvents were purchased from commercial suppliers and were used directly without further purification. Column chromatography was conducted using 200–300 mesh silica gel.

### 3.2. Chemistry

#### 3.2.1. Synthetic Procedure of the Intermediates 2-Mercapto-*N*-arylacetamide (**2a–f**)

A solution of substituted aniline (10 mmol) and mercapto acetic acid (1.105 g, 12 mmol) in toluene (4 mL) was stirred under a nitrogen atmosphere and warmed to 100 °C for 24 h. After cooling down, the reaction solution was diluted with dichloromethane (15 mL), and the mixture was washed with saturated aqueous sodium bicarbonate, water, 1 M HCl and brine, successively. The organic phase was then dried over anhydrous Na_2_SO_4_. After removal of the desiccant by filtration, the organic solution was condensed under reduced pressure to afford the intermediate **2**(**a–f**), which was pure enough and could be directly used for the reaction in the next step.

*N*-(3-fluorophenyl)-2-mercaptoacetamide (**2a**): white solid, yield 78%, m.p. 88–89 °C; ^1^H NMR (CDCl_3_, 400 MHz) *δ*: 8.60 (s, 1H, NH), 7.52 (d, *J* = 11.3 Hz, 1H, Ph-H), 7.29 (d, *J* = 7.7 Hz, 1H, Ph-H), 7.19 (d, *J* = 8.2 Hz, 1H, Ph-H), 6.85 (t, *J* = 8.3 Hz, 1H, Ph-H), 3.41 (d, *J* = 9.3 Hz, 2H, CH_2_), 2.04 (t, *J* = 9.2 Hz, 1H, SH).

*N*-(2-fluorophenyl)-2-mercaptoacetamide (**2b**): white solid, yield 51%, m.p. 101–103 °C; ^1^H NMR (CDCl_3_, 400 MHz) *δ*: 8.72 (s, 1H, NH), 8.17 (t, *J* = 7.8 Hz, 1H, Ph-H), 6.98–7.06 (m, 3H, Ph-H), 3.34 (s, 2H, CH_2_), 2.00 (s, 1H, SH).

*N*-(4-fluorophenyl)-2-mercaptoacetamide (**2c**): white solid, yield 59%, m.p. 167–168 °C; ^1^H NMR (CDCl_3_, 400 MHz) *δ*: 8.53 (s, 1H, NH), 7.47–7.56 (m, 2H, Ph-H), 7.01–7.08 (m, 2H, Ph-H), 3.40 (d, *J* = 9.3 Hz, 2H, CH_2_), 2.03 (t, *J* = 9.3 Hz, 1H, SH).

2-mercapto-*N*-(*m*-tolyl)acetamide (**2d**): white solid, yield 78%, m.p. 53–54 °C (lit. [22] m.p. 150 °C); ^1^H NMR (CDCl_3_, 400 MHz) *δ*: 8.48 (s, 1H, NH), 7.38–7.45 (m, 1H, Ph-H), 7.34 (d, *J* = 8.1 Hz, 1H, Ph-H), 7.23 (t, *J* = 7.8 Hz, 1H, Ph-H), 6.96 (d, *J* = 7.4 Hz, 1H, Ph-H), 3.39 (d, *J* = 9.2 Hz, 2H, CH_2_), 2.35 (s, 3H, CH_3_).

2-mercapto-*N*-(3-methoxyphenyl)acetamide (**2e**): white solid, yield 66%, m.p. 91–92 °C; ^1^H NMR (CDCl_3_, 400 MHz) *δ*: 8.54 (s, 1H, NH), 7.32 (t, *J* = 2.0 Hz, 1H, Ph-H), 7.26 (t, *J* = 8.1 Hz, 1H, Ph-H), 7.05 (d, *J* = 9.0 Hz, 1H, Ph-H), 6.72 (dd, *J* = 8.1, 2.2 Hz, 1H, Ph-H), 3.83 (s, 3H, CH_3_), 3.41 (d, *J* = 9.2 Hz, 2H, CH_2_).

2-mercapto-*N*-(2-(trifluoromethyl)phenyl)acetamide (**2f**): white solid, yield 73%, m.p. 93–95 °C; ^1^H NMR (CDCl_3_, 400 MHz) *δ*: 9.06 (s, 1H, NH), 8.22 (d, *J* = 8.3 Hz, 1H, Ph-H), 7.64 (d, *J* = 7.9 Hz, 1H, Ph-H), 7.58 (t, *J* = 7.8 Hz, 1H, Ph-H), 7.27 (d, *J* = 8.7 Hz, 1H, Ph-H), 3.46 (d, *J* = 9.4 Hz, 2H, CH_2_).

#### 3.2.2. Synthetic Procedure of the Intermediates 2-Chloro-*N*-arylacetamide (**2g–k**)

To a mixture of substituted aniline (5 mmol) and anhydrous K_2_CO_3_ (1.036 g, 7.5 mmol) in CH_3_CN (10 mL), a solution of chloroacetyl chloride (0.60 mL, 7.5 mmol) in CH_3_CN (5 mL) was added dropwise at 0 °C. After that, the reaction system was warmed to room temperature naturally, then was kept at 50 °C with stirring for 2 h and cooled down. The mixture was concentrated under reduced pressure. The residue was diluted with dichloromethane (15 mL), and the mixture was washed with water, 1 M HCl and brine, successively. The organic phase was dried over anhydrous Na_2_SO_4_ and concentrated in vacuo to afford the intermediate **2**(**g–k**). The crude product was pure enough and could be directly used for the reaction in the next step.

2-chloro-*N*-(3-(trifluoromethyl)phenyl)acetamide (**2g**): white solid, yield 53%, m.p. 53–54 °C (lit. [23] m.p. 73.3–75.5 °C); ^1^H NMR (CDCl_3_, 400 MHz) *δ*: 8.45 (s, 1H, NH), 7.84 (s, 1H, Ph-H), 7.75 (d, *J* = 7.9 Hz, 1H), 7.40–7.49 (m, 2H, Ph-H), 4.20 (s, 2H, CH_2_).

2-chloro-*N*-(3-nitrophenyl)acetamide (**2h**): yellow solid, yield 51%, m.p. 85–86 °C (lit. [24] m.p. 90 °C); ^1^H NMR (CDCl_3_, 400 MHz) *δ*: 8.53 (s, 1H, NH), 8.45 (s, 1H, Ph-H), 8.02 (d, *J* = 7.3 Hz, 1H, Ph-H), 7.95 (d, *J* = 8.1 Hz, 1H, Ph-H), 7.54 (t, *J* = 8.2 Hz, 1H, Ph-H), 4.24 (s, 2H, CH_2_).

2-chloro-*N*-(4-chloro-2-nitrophenyl)acetamide (**2i**): yellow solid, yield 76%, m.p. 137–138 °C (lit. [25] m.p. 141–142 °C); ^1^H NMR (CDCl_3_, 400 MHz) *δ*: 11.32 (s, 1H, NH), 8.78 (d, *J* = 9.1 Hz, 1H, Ph-H), 8.26 (s, 1H, Ph-H), 7.65 (d, *J* = 11.6 Hz, 1H, Ph-H), 4.25 (s, 2H, CH_2_).

2-chloro-*N*-(2,6-dichloro-4-fluorophenyl)acetamide (**2j**): white solid, yield 65%, m.p. 149–151 °C; ^1^H NMR (CDCl_3_, 400 MHz) *δ*: 8.02 (s, 1H, NH), 7.42 (s, 2H, Ph-H), 4.27 (s, 2H, CH_2_).

2-chloro-*N*-(2,4,6-trichlorophenyl)acetamide (**2k**): white solid, yield 64%, m.p. 181–182 °C (lit. [26] m.p. 182 °C); ^1^H NMR (CDCl_3_, 400 MHz) *δ*: 7.99 (s, 1H, NH), 7.19 (s, 1H, Ph-H), 7.17 (s, 1H, Ph-H), 4.27 (s, 2H, CH_2_).

#### 3.2.3. Representative Synthetic Procedure of the Intermediates 4-(5-Substitutedthiophen-2-yl)-6-methyl-2-oxo-1,2-dihydropyridine-3-carbonitrile (**6a–d**)

To a stirred solution of 5-substitutedthiophene-2-carbaldehyde (40 mmol), ethyl cyanoacetate (4.524 g, 40 mmol) and acetone (2.323 g, 40 mmol) in ethanol (40 mL) and ammonium acetate (12.333 g, 160 mmol) was added. The reaction system was refluxed for 12 h and cooled down. Then the mixture was filtered, and the filter cake was washed with ethanol to afford the intermediate **6**(**a–d**) with high purity, which was directly used for the reaction in the next step. Taking 6-methyl-2-oxo-4-(thiophen-2-yl)-1,2-dihydropyridine-3-carbonitrile (**6a**) as an example for characterization: yellow solid, yield 94%, m.p. 270–271 °C (lit. [27] m.p. > 250 °C); ^1^H NMR (DMSO-*d*_6_, 400 MHz) *δ*: 12.45 (br, 1H, NH), 7.97 (d, *J* = 5.0 Hz, 1H, Py-H), 7.91–7.95 (m, 1H, Thiophene-H), 7.31 (dd, *J* = 4.9, 3.9 Hz, 1H, Thiophene-H), 6.52 (s, 1H, Thiophene-H), 2.30 (s, 3H, CH_3_).

#### 3.2.4. Representative Synthetic Procedure of the Intermediates 2-Chloro-4-(5-chlorothiophen-2-yl)-6-methylnicotinonitrile (**7a–d**)

A mixture of compound **6** (30 mmol) and phosphorus oxychloride (20 mL) was heated to reflux for 5 h and cooled down. It was slowly poured into warm water with stirring. After cooling, the mixture was added to saturated aqueous NaHCO_3_ to adjust the pH 7–8. The precipitate was collected by filtration and washed with water to give the intermediate **7**(**a–d**) with high purity, which was directly used for the reaction in the next step. Taking 2-chloro-6-methyl-4-(thiophen-2-yl)nicotinonitrile (**7a**) as an example for characterization: green solid, yield 89%, m.p. 121–122 °C; ^1^H NMR (Acetone-*d*_6_, 400 MHz) *δ*: 7.97 (s, 1H, Py-H), 7.84–7.90 (m, 1H, Thiophene-H), 7.62 (s, 1H Thiophene-H), 7.27–7.35 (m, 1H, Thiophene-H), 2.60 (s, 3H, CH_3_).

#### 3.2.5. Synthetic Procedure of Intermediate 6-Methyl-4-(thiophen-2-yl)-2-thioxo-1,2-dihydropyridine-3-carbonitrile (**8**)

Thiourea (3.045 g, 40 mmol) was added to a solution of compound **7a** (4.694 g, 20 mmol) in ethanol (50 mL). The reaction mixture was stirred at reflux for 8 h with TLC monitoring. After cooling down, the precipitate was collected by filtration and washed with ethanol to give the intermediate **8** as a yellow solid, with yield 88%, m.p. 156–157 °C; ^1^H NMR (Acetone-*d*_6_, 400 MHz) *δ*: 12.56 (br, 1H, NH), 8.06 (s, 1H, Py-H), 7.92 (d, *J* = 4.0 Hz, 1H, Thiophene-H), 7.24–7.39 (m, 1H, Thiophene-H), 6.92 (s, 1H, Thiophene-H), 2.56 (s, 3H, CH_3_).

#### 3.2.6. Synthetic Procedure of the Target Compounds **A** and **Ia–Im**

Anhydrous potassium carbonate (0.207 g, 1.5 mmol) was added to a solution of compound **2**(**a–f**) (1 mmol) and **7**(**a–d**) (1 mmol) (path A), or compound **2**(**g–k**) (1 mmol) and **8** (0.232 g, 1 mmol) (path B) in CH_3_CN (10 mL). The reaction system was stirred at room temperature for 2 h and concentrated in vacuo. The residue was diluted with dichloromethane (15 mL), and the mixture was washed with water and brine, successively. The organic layer was dried over anhydrous Na_2_SO_4_. After removing the solvent, column chromatography was conducted on the residue with petroleum ether and ethyl acetate (3:1, *v*/*v*) as eluent to afford the target compounds **A** and **Ia–Im**.

2-((3-cyano-6-methyl-4-(thiophen-2-yl)pyridin-2-yl)thio)-*N*-(3-fluorophenyl) acetamide (**A**): white solid, yield 62%, m.p. 156–157 °C; ^1^H NMR (CDCl_3_, 400 MHz) *δ*: 9.32 (s, 1H, NH), 7.87–7.94 (m, 1H, Py-H), 7.55–7.61 (m, 1H, Ph-H), 7.44 (dt, *J* = 10.8, 2.2 Hz, 1H, Thiophene-H), 7.18–7.26 (m, 3H, Thiophene-H, Ph-H), 7.06–7.10 (m, 1H, Ph-H), 6.79 (td, *J* = 8.3, 2.2 Hz, 1H, Ph-H), 4.00 (s, 2H, CH_2_), 2.69 (s, 3H, CH_3_); ^13^C NMR (CDCl_3_, 101 MHz) *δ*: 166.95 (C=O), 164.24 (Py-C), 162.81 (Ph-H), 161.61, 146.23 (Py-C), 139.38 (d, *J* = 11.11 Hz, Ph-C), 136.52 (Thiophene-C), 130.13 (d, *J* = 9.09 Hz, Ph-C), 130.03, 130.01, 128.97 (Thiophene-C), 118.97 (CN), 115.50 (Py-C), 114.91 (d, *J* = 3.03 Hz, Ph-C), 111.09 (d, *J* = 21.21 Hz, Ph-C), 107.25 (d, *J* = 26.26 Hz, Ph-C), 101.10 (Py-C), 35.10 (CH_2_), 24.96 (CH_3_). HRMS (ESI) calcd for C_19_H_15_FN_3_OS_2_ (M+H)^+^ 384.0641, found 384.0636.

2-((3-cyano-6-methyl-4-(thiophen-2-yl)pyridin-2-yl)thio)-*N*-(2-fluorophenyl)acetamide (**Ia**): brown solid, yield 54%, m.p. 151–152 °C; ^1^H NMR (CDCl_3_, 400 MHz) *δ*: 9.36 (s, 1H, NH), 8.34 (t, *J* = 7.9 Hz, 1H, Ph-H), 7.91 (d, *J* = 3.3 Hz, 1H, Py-H), 7.57 (d, *J* = 4.9 Hz, 1H, Thiophene-H), 7.17–7.23 (m, 2H, Thiophene-H), 7.12 (dt, *J* = 8.4, 4.4 Hz, 1H, Ph-H), 6.97–7.05 (m, 2H, Ph-H), 4.05 (s, 2H, CH_2_), 2.68 (s, 3H, CH_3_); ^13^C NMR (CDCl_3_, 101 MHz) *δ*: 167.46 (C=O), 162.85, 162.30 (Py-C), 152.31 (d, *J* = 244.42 Hz, Ph-C), 146.10 (Py-C), 136.64, 129.96, 129.86, 128.92 (Thiophene-C), 126.48 (d, *J* = 10.10 Hz, Ph-C), 124.67 (d, *J* = 4.04 Hz, Ph-C), 124.32 (d, *J* = 8.08 Hz, Ph-C), 121.76 (Ph-C), 118.88 (CN), 115.52 (Py-C), 114.71 (d, *J* = 19.19 Hz, Ph-C), 100.77 (Py-C), 34.82 (CH_2_), 24.74 (CH_3_). HRMS(ESI) calcd for C_19_H_14_FN_3_OS_2_ (M+H)^+^ 384.0641, found 384.0638.

2-((3-cyano-6-methyl-4-(thiophen-2-yl)pyridin-2-yl)thio)-*N*-(4-fluorophenyl)acetamide (**Ib**): gray solid, yield 59%, m.p. 175–177 °C; ^1^H NMR (CDCl_3_, 400 MHz) *δ*: 9.17 (s, 1H, NH), 7.91 (s, 1H, Py-H), 7.58 (d, *J* = 4.8 Hz, 1H, Thiophene-H), 7.43 (dd, *J* = 8.5, 4.7 Hz, 2H, Ph-H), 7.21 (dd, *J* = 9.0, 4.4 Hz, 2H, Ph-H), 7.00 (t, *J* = 8.5 Hz, 2H, Thiophene-H), 4.01 (s, 2H, CH_2_), 2.67 (s, 3H, CH_3_); ^13^C NMR (CDCl_3_, 101 MHz) *δ*: 166.75 (C=O), 162.76, 161.61 (Py-C), 159.34 (d, *J* = 245.43 Hz, Ph-C), 146.14 (Py-C), 136.55 (Thiophene-C), 133.86 (d, *J* = 3.03 Hz, Ph-C), 129.96 (Ph-C), 128.93, 121.57, 121.49 (Thiophene-C), 118.86 (CN), 115.67 (d, *J* = 23.23 Hz, Ph-C), 115.55, 101.02 (Py-C), 34.94 (CH_2_), 24.93 (CH_3_). HRMS (ESI) calcd for C_20_H_15_F_3_N_3_OS_2_ (M+H)^+^ 434.0609, found 434.0605.

2-((3-cyano-6-methyl-4-(thiophen-2-yl)pyridin-2-yl)thio)-*N*-(m-tolyl)acetamide (**Ic**): yellow solid, yield 51%, m.p. 155–156 °C; ^1^H NMR (CDCl_3_, 400 MHz) *δ*: 9.07 (s, 1H, NH), 7.89–7.91 (m, 1H, Thiophene-H), 7.55–7.58 (m, 1H, Ph-H), 7.34 (s, 1H, Py-H), 7.17–7.22 (m, 4H, Ph-H, Thiophene-H), 6.91 (d, *J* = 7.2 Hz, 1H, Ph-H), 4.01 (s, 2H, CH_2_), 2.67 (s, 3H, Py-CH_3_), 2.32 (s, 3H, Ph-CH_3_); ^13^C NMR (CDCl_3_, 101 MHz) *δ*: 166.67 (C=O), 162.81, 161.66, 146.11 (Py-C), 139.02, 137.73 (Ph-C), 136.62 (Thiophene-C), 129.94 (Ph-C), 129.88, 128.93, 128.86 (Thiophene-C), 125.22, 120.44, 118.84 (Ph-C), 116.81 (CN), 115.58, 100.98 (Py-C), 35.03 (CH_2_), 24.96 (Py-CH_3_), 21.52 (Ph-CH_3_). HRMS (ESI) calcd for C_20_H_18_N_3_OS_2_ (M+H)^+^ 380.0891, found 380.0885.

2-((3-cyano-6-methyl-4-(thiophen-2-yl)pyridin-2-yl)thio)-*N*-(3-methoxyphenyl)acetamide (**Id**): brown solid, yield 43%, m.p. 149–151 °C; ^1^H NMR (CDCl_3_, 400 MHz) *δ*: 9.17 (s, 1H, NH), 7.90 (d, *J* = 3.4 Hz, 1H, Py-H), 7.57 (d, *J* = 4.9 Hz, 1H, Thiophene-H), 7.16–7.26 (m, 4H, Ph-H, Thiophene-H), 6.91 (d, *J* = 7.7 Hz, 1H, Thiophene-H), 6.65 (d, *J* = 7.9 Hz, 1H, Ph-H), 4.00 (s, 2H, CH_2_), 3.79 (s, 3H, OCH_3_), 2.68 (s, 3H, CH_3_); ^13^C NMR (CDCl_3_, 101 MHz) *δ*: 166.82 (C=O), 162.85, 161.63 (Py-C), 160.20 (Ph-C), 146.14 (Py-C), 139.05 (Ph-C), 136.58 (Thiophene-C), 129.97 (Ph-C), 129.92, 129.71, 128.94 (Thiophene-C), 118.86 (Ph-C), 115.57 (CN), 111.83, 110.14 (Ph-C), 105.59, 100.99 (Py-C), 55.33 (OCH_3_), 35.11(CH_2_), 24.97(CH_3_). HRMS (ESI) calcd for C_20_H_18_N_3_O_2_S_2_ (M+H)^+^ 396.0840, found 396.0836.

2-((3-cyano-6-methyl-4-(thiophen-2-yl)pyridin-2-yl)thio)-*N*-(2-(trifluoromethyl)phenyl) acetamide (**Ie**): yellow solid, yield 37%, m.p. 170–171 °C; ^1^H NMR (CDCl_3_, 400 MHz) *δ*: 8.76 (s, 1H, NH), 8.10 (d, *J* = 8.3 Hz, 1H, Py-H), 7.92 (d, *J* = 3.6 Hz, 1H, Thiophene-H), 7.53–7.59 (m, 3H, Ph-H), 7.14–7.25 (m, 3H, Ph-H, Thiophene-H), 4.13 (s, 2H, CH_2_), 2.56 (s, 3H, CH_3_); ^13^C NMR (CDCl_3_, 101 MHz) *δ*: 167.02 (C=O), 162.26, 161.55, 145.89 (Py-C), 137.98 (Thiophene-C), 136.77, 134.90 (Ph-C), 132.81, 129.81, 129.66 (Thiophene-C), 128.85 (Ph-C), 126.06 (q, *J* = 5.05 Hz, Ph-C), 125.24, 124.90 (Ph-C), 123.74 (d, *J* = 273.71 Hz, CF_3_), 118.76 (Py-C), 115.61 (CN), 100.73 (Py-C), 34.44 (CH_2_), 24.69 (CH_3_). HRMS (ESI) calcd for C_20_H_15_F_3_N_3_OS_2_ (M+H)^+^ 434.0609, found 434.0605.

2-((3-cyano-6-methyl-4-(thiophen-2-yl)pyridin-2-yl)thio)-*N*-(3-(trifluoromethyl)phenyl) acetamide (**If**): white solid, yield 48%, m.p. 158–159 °C; ^1^H NMR (CDCl_3_, 400 MHz) *δ*: 9.47 (s, 1H, NH), 7.90 (d, *J* = 3.6 Hz, 1H, Ph-H), 7.70–7.72 (m, 2H, Py-H, Ph-H), 7.58 (d, *J* = 5.0 Hz, 1H, Thiophene-H), 7.42 (t, *J* = 7.8 Hz, 1H, Ph-H), 7.34 (d, *J* = 7.7 Hz, 1H, Thiophene-H), 7.19–7.21 (m, 2H, Ph-H, Thiophene-H), 4.02 (s, 2H, CH_2_), 2.68 (s, 3H, CH_3_); ^13^C NMR (CDCl_3_, 101 MHz) *δ*: 167.19 (C=O), 162.74, 161.62, 146.25 (Py-C), 138.36 (Ph-C), 136.46 (Thiophene-C), 131.39 (q, *J* = 33.33 Hz, Ph-C), 130.07 (Ph-C), 130.03, 129.66, 128.95 (Thiophene-C), 123.78 (d, *J* = 273.71 Hz, CF_3_), 122.77, 122.42 (Py-C), 120.90 (q, *J* = 5.05 Hz, Ph-C), 119.04 (Py-C), 116.38 (q, *J* = 4.04 Hz, Py-C), 115.52 (CN), 101.07 (Py-C), 35.11 (CH_2_), 24.87 (CH_3_). HRMS (ESI) calcd for C_20_H_15_F_3_N_3_OS_2_ (M+H)^+^ 434.0609, found 434.0602.

2-((3-cyano-6-methyl-4-(thiophen-2-yl)pyridin-2-yl)thio)-*N*-(3-nitrophenyl)acetamide (**Ig**): yellow solid, yield 48%, m.p. 196–197 °C; ^1^H NMR (DMSO-*d*_6_, 400 MHz) *δ*: 10.81 (s, 1H, NH), 8.58 (t, *J* = 2.1 Hz, 1H, Ph-H), 7.86–7.92 (m, 3H, Py-H, Ph-H), 7.83 (dd, *J* = 3.7, 1.0 Hz, 1H, Thiophene-H), 7.58 (t, *J* = 8.2 Hz, 1H, Ph-H), 7.32 (s, 1H, Thiophene-H), 7.26 (dd, *J* = 5.0, 3.8 Hz, 1H, Thiophene-H), 4.20 (s, 2H, CH_2_), 2.40 (s, 3H, CH_3_); ^13^C NMR (DMSO-*d*_6_, 101 MHz) *δ*: 167.35 (C=O), 162.83, 162.44 (Py-C), 148.46 (Ph-C), 145.39 (Py-C), 140.55 (Ph-C), 136.71 (Thiophene-C), 131.44 (Ph-C), 130.75, 130.46 (Thiophene-C), 129.23 (Ph-C), 125.53 (Thiophene-C), 118.51, 118.38 (Ph-C), 116.43 (CN), 113.63, 99.45 (Py-C), 35.67 (CH_2_), 24.74 (CH_3_). HRMS (ESI) calcd for C_19_H_15_N_4_O_3_S_2_ (M+H)^+^ 411.0586, found 411.0575.

*N*-(4-chloro-2-nitrophenyl)-2-((3-cyano-6-methyl-4-(thiophen-2-yl)pyridin-2-yl)thio) acetamide (**Ih**): yellow solid, yield 51%, m.p. 214–215 °C; ^1^H NMR (CDCl_3_, 400 MHz) *δ*: 10.80 (s, 1H, NH), 8.69 (d, *J* = 9.1 Hz, 1H, Ph-H), 8.13 (s, 1H, Py-H), 7.82–7.97 (m, 1H, Ph-H), 7.54–7.60 (m, 2H, Thiophene-H), 7.08–7.24 (m, 2H, Ph-H, Thiophene-H), 4.18 (s, 2H, CH_2_), 2.55 (s, 3H, CH_3_); ^13^C NMR (CDCl_3_, 101 MHz) *δ*: 167.85 (C=O), 162.23, 161.17, 145.89 (Py-C), 137.16 (Ph-C), 136.77 (Thiophene-C), 135.60, 132.87, 129.80 (Ph-C), 129.66, 128.92, 128.84 (Thiophene-C), 125.31, 124.05 (Ph-C), 118.87 (Py-C), 115.64 (CN), 100.71 (Py-C), 35.13 (CH_2_), 24.82 (CH_3_). HRMS (ESI) calcd for C_19_H_14_ClN_3_O_2_S_2_ (M+H)^+^ 455.0196, found 455.0187.

2-((3-cyano-6-methyl-4-(thiophen-2-yl)pyridin-2-yl)thio)-*N*-(2,6-dichloro-4-fluorophenyl) acetamide (**Ii**): white solid, yield 49%, m.p. 209–211 °C; ^1^H NMR (CDCl_3_, 400 MHz) *δ*: 8.92 (s, 1H, NH), 7.91 (s, 1H, Py-H), 7.57 (d, *J* = 4.4 Hz, 1H, Thiophene-H), 7.19 (d, *J* = 14.3 Hz, 2H, Ph-H), 7.11 (d, *J* = 7.8 Hz, 2H, Thiophene-H), 4.09 (s, 2H, CH_2_), 2.61 (s, 3H, CH_3_); ^13^C NMR (CDCl_3_, 101 MHz) *δ*: 167.13 (C=O), 162.55, 161.96 (Py-C), 160.40 (d, *J* = 254.52 Hz, Ph-C), 146.06 (Py-C), 136.61 (Thiophene-C), 134.38 (d, *J* = 12.12 Hz, Ph-C), 129.96, 129.92, 128.94 (Thiophene-C), 128.51 (d, *J* = 5.05 Hz, Ph-C), 118.80 (Py-C), 116.19 (d, *J* = 25.25 Hz, Ph-C), 115.64 (CN), 100.84 (Py-C), 34.01 (CH_2_), 24.79 (CH_3_). HRMS (ESI) calcd for C_19_H_13_Cl_2_FN_3_OS_2_ (M+H)^+^ 451.9861, found 451.9854.

2-((3-cyano-6-methyl-4-(thiophen-2-yl)pyridin-2-yl)thio)-*N*-(2,4,6-trichlorophenyl)acetamide (**Ij**): yellow solid, yield 56%, m.p. 212–214 °C; ^1^H NMR (CDCl_3_, 400 MHz) *δ*: 10.20 (s, 1H, NH), 7.91 (d, *J* = 5.0 Hz, 1H, Thiophene-H), 7.86 (d, *J* = 3.3 Hz, 1H, Thiophene-H), 7.72 (s, 2H, Ph-H), 7.39 (s, 1H, Py-H), 7.26–7.32 (m, 1H, Thiophene-H), 4.27 (s, 2H, CH_2_), 2.54 (s, 3H, CH_3_); ^13^C NMR (CDCl_3_, 101 MHz) *δ*: 166.46 (C=O), 162.63, 162.47, 145.33 (Py-C), 136.70 (Thiophene-C), 134.81 (Ph-C), 132.91, 132.67 (Thiophene-C), 131.42, 130.42 (Ph-C), 129.22 (Thiophene-C), 128.73 (Ph-C), 118.57 (Py-C), 116.47 (CN), 99.63 (Py-C), 34.07 (CH_2_), 24.88 (CH_3_). HRMS (ESI) calcd for C_19_H_13_Cl_3_N_3_OS_2_ (M+H)^+^ 467.9566, found 467.9556.

2-((3-cyano-6-methyl-4-(5-methylthiophen-2-yl)pyridin-2-yl)thio)-*N*-(3-fluorophenyl) acetamide (**Ik**): gray solid, yield 53%, m.p. 193–195 °C; ^1^H NMR (CDCl_3_, 400 MHz) *δ*: 9.39 (s, 1H, NH), 7.75 (s, 1H, Py-H), 7.44 (d, *J* = 10.3 Hz, 1H, Ph-H), 7.18–7.26 (m, 1H, Ph-H), 7.13 (s, 1H, Ph-H), 7.08 (d, *J* = 7.0 Hz, 1H, Thiophene-H), 6.75–6.88 (m, 2H, Ph-H, Thiophene-H), 3.98 (s, 2H, CH_2_), 2.66 (s, 3H, Py-CH_3_), 2.56 (s, 3H, Thiophene-CH_3_); ^13^C NMR (CDCl_3_, 101 MHz) *δ*: 167.10 (C=O), 163.02 (d, *J* = 245.43 Hz, Ph-C), 162.78, 161.28, 146.26 (Py-C), 145.79 (Thiophene-C), 139.48, 139.37 (d, *J* = 245.43 Hz, Ph-C), 134.02, 130.50 (Thiophene-C), 130.10 (d, *J* = 10.10 Hz, Ph-C), 127.49 (Thiophene-C), 118.37 (Py-C), 115.75 (CN), 114.91, 114.88 (d, *J* = 3.03 Hz, Ph-C), 111.02 (d, *J* = 22.22 Hz, Ph-C), 107.23 (d, *J* = 26.26 Hz, Ph-C), 100.34 (Py-C), 35.10 (CH_2_), 24.93 (Py-CH_3_), 15.60 (Thiophene-CH_3_). HRMS (ESI) calcd for C_20_H_17_FN_3_OS_2_ (M+H)^+^ 398.0797, found 398.0792.

2-((4-(5-chlorothiophen-2-yl)-3-cyano-6-methylpyridin-2-yl)thio)-*N*-(3-fluorophenyl) acetamide (**Il**): gray solid, yield 53%, m.p. 181–183 °C; ^1^H NMR (CDCl_3_, 400 MHz) *δ*: 9.20 (s, 1H, NH), 7.68 (d, *J* = 3.8 Hz, 1H, Py-H), 7.43 (d, *J* = 10.6 Hz, 1H, Ph-H), 7.21–7.25 (m, 1H, Ph-H), 7.01–7.10 (m, 3H, Ph-H, Thiophene-H), 6.79 (t, *J* = 8.1 Hz, 1H, Ph-H), 4.00 (s, 2H, CH_2_), 2.67 (s, 3H, CH_3_); ^13^C NMR (CDCl_3_, 101 MHz) *δ*: 166.79 (C=O), 163.01 (d, *J* = 246.44 Hz, Ph-C), 163.00, 161.87, 145.11 (Py-C), 139.30 (d, *J* = 11.11 Hz, Ph-C), 135.24, 134.82 (Thiophene-C), 130.14 (d, *J* = 9.09 Hz, Ph-C), 129.65, 128.08 (Thiophene-C), 118.36 (Py-C), 115.26 (CN), 114.92 (d, *J* = 3.03 Hz, Ph-C), 111.15 (d, *J* = 11.11 Hz, Ph-C), 107.26 (d, *J* = 26.26 Hz, Ph-C), 100.66 (Py-H), 35.07 (CH_2_), 24.97 (CH_3_). HRMS (ESI) calcd for C_19_H_14_ClFN_3_OS_2_ (M+H)^+^ 418.0251, found 418.0247.

2-((4-(5-bromothiophen-2-yl)-3-cyano-6-methylpyridin-2-yl)thio)-*N*-(3-fluorophenyl) acetamide (**Im**): gray solid, yield 58%, m.p. 198–200 °C; ^1^H NMR (CDCl_3_, 400 MHz) *δ*: 9.21 (s, 1H, NH), 7.64 (d, *J* = 3.9 Hz, 1H, Py-H), 7.43 (d, *J* = 10.8 Hz, 1H, Ph-H), 7.23 (d, *J* = 6.9 Hz, 1H, Ph-H), 7.16 (d, *J* = 3.9 Hz, 1H, Ph-H, Thiophene-H), 7.07–7.08 (m, 2H, Ph-H, Thiophene-H), 6.79 (t, *J* = 8.1 Hz, 1H, Ph-H), 4.00 (s, 2H, CH_2_), 2.67 (s, 3H, CH_3_); ^13^C NMR (CDCl_3_, 101 MHz) *δ*: 166.80 (C=O), 163.01 (d, *J* = 245.43 Hz, Ph-C), 162.98, 161.88, 145.03 (Py-C), 139.30 (d, *J* = 10.10 Hz, Ph-C), 137.77, 131.78, 130.41 (Thiophene-C), 130.13 (d, *J* = 10.10 Hz, Ph-C), 118.44 (Thiophene-C), 117.80 (Py-C), 115.25 (CN), 114.92 (d, *J* = 3.03 Hz, Ph-C), 111.14 (d, *J* = 21.21 Hz, Ph-C), 107.26 (d, *J* = 26.26 Hz, Ph-C), 100.64 (Py-C), 35.08 (CH_2_), 24.98 (CH_3_). HRMS (ESI) calcd for C_19_H_14_BrFN_3_OS_2_ (M+H)^+^ 461.9746, found 461.9744.

#### 3.2.7. Synthetic Procedure of the Target Compounds **In–Iq**

Compound **A** or **Ib** (1 mmol) was dissolved in dichloromethane (10 mL), and the solution was cooled to 0 °C, then 3-chloroperbenzoic acid (*m*-CPBA) (0.190 g, 1.1 mmol) was added in with stirring. The mixture was further reacted at 0 °C for 3–4 h with TLC monitoring. After removing the solvent, the residue was purified using column chromatography with petroleum ether and ethyl acetate (1:1, *v*/*v*) as eluent to afford the target compound **In** or **Ip**.

To a stirred solution of compound **A** or **Ib** (1 mmol) in dichloromethane (10 mL), *m*-CPBA (0.518 g, 3 mmol) was added at room temperature. The mixture was further reacted for 8 h at room temperature with TLC monitoring. After removing the solvent, the residue was purified using column chromatography with petroleum ether and ethyl acetate (1:1, *v*/*v*) as eluent to afford the target compound **Io** or **Iq**.

2-((3-cyano-6-methyl-4-(thiophen-2-yl)pyridin-2-yl)sulfinyl)-*N*-(3-fluorophenyl)acetamide (**In**): yellow solid, yield 60%, m.p. 177–179 °C; ^1^H NMR (DMSO-*d*_6_, 400 MHz) *δ*: 10.67 (s, 1H, NH), 7.98 (d, *J* = 4.9 Hz, 1H, Ph-H), 7.92 (d, *J* = 3.5 Hz, 1H, Thiophene-H), 7.85 (s, 1H, Py-H), 7.51 (d, *J* = 11.4 Hz, 1H, Ph-H), 7.30–7.38 (m, 2H, Thiophene-H, Ph-H), 7.25 (d, *J* = 8.3 Hz, 1H, Thiophene-H), 6.86–6.94 (m, 1H, Ph-H), 4.29–4.36 (m, 2H, CH_2_), 2.64 (s, 3H, CH_3_); ^13^C NMR (DMSO-*d*_6_, 101 MHz) *δ*: 165.55 (Py-C), 163.80 (C=O), 163.72 (d, *J* = 243.41 Hz, Ph-C), 163.40, 146.14 (Py-C), 140.45 (d, *J* = 11.11 Hz, Ph-C), 136.13 (Thiophene-C), 132.19, 131.20 (Thiophene-C), 131.04 (d, *J* = 9.09 Hz, Ph-C), 129.37 (Thiophene-C), 124.72 (Py-C), 115.50 (d, *J* = 3.03 Hz, Ph-C), 114.80 (CN), 110.93 (d, *J* = 21.21 Hz, Ph-C), 106.54 (d, *J* = 26.26 Hz, Ph-C), 102.56 (Py-H), 60.37 (CH_2_), 24.66 (CH_3_). HRMS (ESI) calcd for C_19_H_15_FN_3_O_2_S_2_ (M+H)^+^ 400.0590, found 400.0587.

3-2-((3-cyano-6-methyl-4-(thiophen-2-yl)pyridin-2-yl)sulfonyl)-*N*-(3-fluorophenyl)acetamide (**Io**): yellow solid, yield 68%, m.p. 115–117 °C; ^1^H NMR (CDCl_3_, 400 MHz) *δ*: 8.81 (s, 1H, NH), 7.96 (d, *J* = 3.6 Hz, 1H, Ph-H), 7.63 (d, *J* = 5.0 Hz, 1H, Thiophene-H), 7.58 (s, 1H, Py-H), 7.46 (d, *J* = 10.6 Hz, 1H, Ph-H), 7.19–7.25 (m, 2H, Thiophene-H, Ph-H), 7.14 (d, *J* = 8.1 Hz, 1H, Thiophene-H), 6.80 (t, *J* = 7.4 Hz, 1H, Ph-H), 4.65 (s, 2H, CH_2_), 2.67 (s, 3H, CH_3_); ^13^C NMR (CDCl_3_, 101 MHz) *δ*: 162.85 (d, *J* = 245.43 Hz, Ph-C), 162.31 (C=O), 159.36, 158.53, 148.24 (Py-C), 138.52 (d, *J* = 10.10 Hz, Ph-C), 135.47, 131.18, 130.99 (Thiophene-C), 130.25 (d, *J* = 10.10 Hz, Ph-C), 129.27 (Thiophene-C), 125.96 (Py-C), 115.49 (d, *J* = 3.03 Hz, Ph-C), 113.24 (CN), 111.81 (d, *J* = 21.21 Hz, Ph-C), 107.70 (d, *J* = 26.26 Hz, Ph-C), 100.38 (Py-C), 58.50 (CH_2_), 24.52 (CH_3_). HRMS (ESI) calcd for C_19_H_15_FN_3_O_3_S_2_ (M+H)^+^ 416.0539, found 416.0534.

2-((3-cyano-6-methyl-4-(thiophen-2-yl)pyridin-2-yl)sulfinyl)-*N*-(4-fluorophenyl)acetamide (**Ip**): white solid, yield 58%, m.p. 183–184 °C; ^1^H NMR (DMSO-*d*_6_, 400 MHz) *δ*: 10.55 (s, 1H, NH), 8.00–8.04 (m, 1H, Thiophene-H), 7.95–7.98 (m, 1H, Thiophene-H), 7.88 (s, 1H, Py-H), 7.57–7.63 (m, 2H, Ph-H), 7.37 (dd, J = 4.9, 3.9 Hz, 1H, Thiophene-H), 7.19 (t, *J* = 8.9 Hz, 2H, Ph-H), 4.30–4.42 (m, 2H, CH_2_), 2.69 (s, 3H, CH_3_); ^13^C NMR (DMSO-*d*_6_, 101 MHz) *δ*: 165.11 (C=O), 163.43, 162.41 (Py-C), 157.15 (Ph-C), 145.70 (Py-C), 135.66 (Thiophene-C), 134.69 (d, *J* = 3.03 Hz, Ph-C), 131.71, 130.75, 128.94 (Thiophene-C), 124.26 (Py-C), 121.15 (d, *J* = 8.08 Hz, Ph-C), 115.48(d, *J* = 22.22 Hz, Ph-C), 114.36 (CN), 102.07 (Py-C), 59.77 (CH_2_), 24.22 (CH_3_). HRMS (ESI) calcd for C_19_H_15_FN_3_O_2_S_2_ (M+H)^+^ 400.0590, found 400.0580.

2-((3-cyano-6-methyl-4-(thiophen-2-yl)pyridin-2-yl)sulfonyl)-*N*-(4-fluorophenyl)acetamide (**Iq)**: gray solid, yield 70%, m.p. 141–142 °C; ^1^H NMR (CDCl_3_, 400 MHz) *δ*: 10.57 (s, 1H, NH), 7.99 (s, 1H, Py-H), 7.96 (dd, *J* = 3.8, 1.1 Hz, 1H, Thiophene-H), 7.97–7.90 (m, 1H, Thiophene-H), 7.50–7.60 (m, 3H, Ph-H, Thiophene-H), 7.14–7.21 (m, 2H, Ph-H), 4.85 (s, 2H, CH_2_), 2.65 (s, 3H, CH_3_); ^13^C NMR (CDCl_3_, 101 MHz) *δ*: 166.54 (C=O), 162.80, 160.07 (Py-C), 147.75 (Py-C), 135.77 (Thiophene-C), 135.03 (d, *J* = 3.03Hz, Ph-C), 133.80, 131.79, 131.10 (Thiophene-C), 128.37, 126.59 (Py-C), 121.63 (d, *J* = 8.08 Hz, Ph-C), 116.01 (d, *J* = 22.22 Hz, Ph-C), 114.10 (CN), 100.58 (Py-C), 58.76 (CH_2_), 24.42 (CH_3_). HRMS (ESI) calcd for C_19_H_15_FN_3_O_3_S_2_ (M+H)^+^ 416.0539, found 416.0529.

### 3.3. Biological Assay

#### 3.3.1. Insecticidal Assay against Oriental Armyworm (*Mythimna separata* Walker)

Larvicidal activity of the target compounds and the control insecticide chlorantraniliprole against the oriental armyworm were tested at 25 ± 1 °C through the reported leaf-dip method [28,29]. Leaf disks (5 × 1 cm) cut from fresh corn leaves were dipped into the test solution for 3–5 s. After air-drying, the treated leaf disks were put individually into Petri dishes (7 cm). Each dry treated leaf disk was infested with 10 fourth-instar larvae of oriental armyworm. The percentage of lethalities were evaluated four days after treatment. Leaves treated with acetone were used as blank controls. Each treatment was made three times. Assessments were made on a dead/alive basis, and lethality rates were corrected using Abbott’s formula. Evaluations were based on a percentage scale of 0–100, in which 0 = no activity and 100 = total kill. The insecticidal activity data of the tested compounds are listed in Table 1.

#### 3.3.2. Insecticidal Assay against Diamondback Moth (*Plutella xylostella* L.)

The insecticidal assay of the compounds and the control insecticides chlorantraniliprole, cartap and triflumuron against diamondback moth were conducted at 25 ± 1 °C by the reported leaf-dip method [28,29]. Leaf disks (5 × 1 cm) cut from fresh cabbage leaves were dipped into the test solution for 3 s. After being dried in air, the treated leaf disks were put individually into boxes. Each dry treated leaf disk was infested with 10 second-instar larvae of diamondback moth. The percentage of lethalities were evaluated three days after treatment. Leaves treated with water and acetone were used as blank controls. Each treatment was made three times. Assessments were made on a dead/alive basis, and lethality rates were corrected using Abbott’s formula. Evaluations were based on a percentage scale of 0–100, in which 0 = no activity and 100 = total kill. The insecticidal activity data of the tested compounds are listed in Table 1 and Table 2.

#### 3.3.3. Fungicidal Assay In Vitro

The in vitro fungicidal activity of the synthesized compounds against Sclerotinia sclerotiorum, Botrytis cinerea, Pellicularia sasakii, Fusarium oxysporum, Physalospora piricola and Rhizoctonia cerealis were tested using the reported procedure of mycelium growth rate protocol [30,31]. The commercial fungicides chlorothalonil, azoxystrobin, and triadimefon were evaluated as controls in the same conditions. The test was performed in an isolated culture. Under a sterile condition, 1 mL sample solution of the test compound in DMSO was added to the culture plates, followed by the addition of 9 mL of the culture medium. The final test concentration was 50 μg/mL. The blank assay was performed with 1 mL of sterile water. Circle mycelium with a diameter of 4 mm was cut using a drill. The culture plates were cultivated at 24 ± 1 °C. The extended diameters of the circle mycelium were measured after 72 h. The relative inhibition rate of the circle mycelium compared to the blank assay was calculated through the following equation:

Relative inhibition rate (%) = ((CK − PT)/CK) × 100%

where CK is the extended diameter (mm) of the circle mycelium during the blank assay, and PT is the extended diameter (mm) of the circle mycelium during testing.

The in vitro fungicidal activity data of the tested compounds are listed in Table 3.

## 4. Conclusions

In summary, according to the structural information of the “hit” compound **A** from the reported pharmacophore-based virtual screening by Sindhu et al., a series of novel thienylpyridyl- and thioether/sulfoxide/sulfone-containing acetamide derivatives have been designed and synthesized via different routes in this paper. The structures of new compounds were confirmed by ^1^H NMR, ^13^C NMR and HRMS. The single-crystal structure of **A** was firstly reported. In addition to the first validation of compound **A** for insecticidal activities in our lab, comprehensive insecticidal evaluations of the newly synthesized compounds on *Mythimna separata* Walker and *Plutella xylostella* L. were conducted. Through a step-by-step structural optimization, the high-insecticidal agents, especially towards *Plutella xylostella* L., have been found, and thienylpyridyl- and sulfone/thioether-containing acetamides **Iq**, **Io**, **Ib** and **A** can serve as novel lead structures for innovative research on new insecticides. Moreover, some of the compounds, e.g., **A**, **Ih**, **Id**, **Io** and **Iq**, were also found to have favourable fungicidal activities against *Physalospora piricola*, *Rhizoctonia cerealis* and *Sclerotinia sclerotiorum* and would provide useful guidance for the design and development of new fungicides.

## Figures and Tables

**Table 1:** Insecticidal activities of the target compounds **A** and **Ia–q**.

Compd.	Lethality Rate (%) against Mythimna Separata Walker at 200 mg/L	Lethality Rate (%) against *Plutella xylostella* L. at Conc. (mg/L)						
		200	100	10	1	0.1	0.01	0.001
**A**	60	100	100	90	60	n.t. ^a^	n.t.	n.t.
**Ia**	20	100	90	75	n.t.	n.t.	n.t.	n.t.
**Ib**	30	100	100	100	90	70	n.t.	n.t.
**Ic**	20	85	60	37	n.t.	n.t.	n.t.	n.t.
**Id**	40	100	93	80	n.t.	n.t.	n.t.	n.t.
**Ie**	30	93	71	55	n.t.	n.t.	n.t.	n.t.
**If**	20	92	77	47	n.t.	n.t.	n.t.	n.t.
**Ig**	0	100	100	85	50	n.t.	n.t.	n.t.
**Ih**	35	100	88	63	20	n.t.	n.t.	n.t.
**Ii**	40	90	73	40	n.t.	n.t.	n.t.	n.t.
**Ij**	30	80	47	n.t.	n.t.	n.t.	n.t.	n.t.
**Ik**	70	50	n.t.	n.t.	n.t.	n.t.	n.t.	n.t.
**Il**	50	74	55	n.t.	n.t.	n.t.	n.t.	n.t.
**Im**	30	65	n.t.	n.t.	n.t.	n.t.	n.t.	n.t.
**In**	50	40	n.t.	n.t.	n.t.	n.t.	n.t.	n.t.
**Io**	30	100	100	88	70	n.t.	n.t.	n.t.
**Ip**	50	100	90	57	20	n.t.	n.t.	n.t.
**Iq**	30	100	100	100	100	100	70	43
chlorantraniliprole	100	100	100	100	100	100	100	83 (37 ^b^)
cartap	n.t.	93	67	40	n.t.	n.t.	n.t.	n.t.
triflumuron	n.t.	100	100	77	50	n.t.	n.t.	n.t.

^a^ n.t. = not test; ^b^ at a test conc. of 0.0001 mg/L.

**Table 2:** LC_50_ values of compounds **Iq**, triflumuron and chlorantraniliprole against the diamondback moth (*Plutella xylostella* L.).

Compd.	*y* = a + b*x*	LC_50_ (mg/L)	*R*
**Iq**	*y* = 8.47 + 1.24*x*	0.0016	0.9394
triflumuron	*y* = 5.31 + 0.71*x*	0.3627	0.9697
chlorantraniliprole	*y* = 9.75 + 1.13*x*	0.0001	0.9570

**Table 3:** In vitro fungicidal activities of the target compounds **A** and **Ia–q** at 50 μg/mL.

Compd.	% Growth Inhibition					
	*Sclerotinia sclerotiorum*	*Botrytis cinerea*	*Pellicularia sasakii*	*Fusarium oxysporum*	*Physalospora piricola*	*Rhizoctonia cerealis*
**A**	60.7	32.3	36.1	10.8	44.7	69.2
**Ia**	8.9	25.8	11.1	13.5	44.7	47.7
**Ib**	17.9	29.0	13.9	10.8	39.5	32.3
**Ic**	14.3	12.9	5.6	10.8	71.1	13.8
**Id**	53.6	6.5	27.8	13.5	81.6	33.8
**Ie**	17.9	25.8	13.9	10.8	55.3	52.3
**If**	17.9	16.1	5.6	13.5	44.7	36.9
**Ig**	25.0	22.6	13.9	8.1	7.9	29.2
**Ih**	8.9	25.8	27.8	16.2	44.7	67.7
**Ii**	17.9	6.5	25.0	10.8	44.7	38.5
**Ij**	17.9	16.1	27.8	8.1	21.1	29.2
**Ik**	7.1	9.7	5.6	2.7	44.7	38.5
**Il**	17.9	29.0	13.9	8.1	57.9	52.3
**Im**	14.3	16.1	11.1	8.1	23.7	21.5
**In**	44.6	16.1	19.4	8.1	31.6	43.1
**Io**	91.1	41.9	27.8	16.2	57.9	35.4
**Ip**	80.4	35.5	27.8	10.8	71.1	21.5
**Iq**	85.7	45.2	11.1	16.2	44.7	36.9
chlorothalonil	98.2	74.2	100.0	73.0	84.2	89.2
azoxystrobin	92.9	29.0	66.7	59.5	92.1	96.9
triadimefon	98.2	64.5	83.3	70.3	65.8	96.9

## Data Availability

Not applicable.

## Supplementary Materials

The following are available online, Figures S1–S14: ^1^H NMR spectra of compounds **2a–2k**, **6a**, **7a** and **8**. Figures S15–S50: ^1^H NMR and ^13^C NMR spectra of compounds **A** and **Ia–Iq**. Figures S51–S68: HRMS of compounds **A** and **Ia–Iq**. Table S1: Crystal data and structure refinement for compound **A**. Table S2: Fractional atomic coordinates (×10^4^) and equivalent isotropic displacement parameters (Å^2^ × 10^3^) for compound **A** (U(eq) is defined as one-third of the trace of the orthogonalised Uij tensor). Table S3: Bond lengths (Å) and angles (°) for compound **A**. Table S4: Torsion angles (°) for compound **A**.

## Sample Availability

No applicable.
