# Supplementary material for: Design, Synthesis and Biological Evaluation of Novel Thienylpyridyl- and Thioether-Containing Acetamides and Their Derivatives as Pesticidal Agents
Source: Molecules. 2021 Sep 17;26(18):5649. doi: 10.3390/molecules26185649 (PMC8466124; doi:10.3390/molecules26185649)
Supplement: Supplementary file 1 [file molecules-26-05649-s001.zip › molecules-1372823-supplementary.pdf]

Supplementary Materials:

Design, Synthesis and Biological Evaluation of Novel Thienylpyridyl- and Thioether-Containing Acetamides and Their Derivatives As Pesticidal Agents

Huan Li, Na Yang, Lixia Xiong and Baolei Wang *

State Key Laboratory of Elemento-Organic Chemistry, College of Chemistry, Nankai University, Tianjin 300071, China; 2120180790@mail.nankai.edu.cn (H.L.); flyna2010@nankai.edu.cn (N.Y.); xionglixia@nankai.edu.cn (L.X.)
* Correspondence: nkwbl@nankai.edu.cn (B.W.)

1. **The NMR spectrogram of the intermediates and title compounds…..….….... S2**
2. **The HRMS of compounds A and Ia-Iq…………..…………………...…....... S27**
3. **Crystal structure determination……………………..……………………….S45**

1. The NMR spectrogram of the intermediates and title compounds

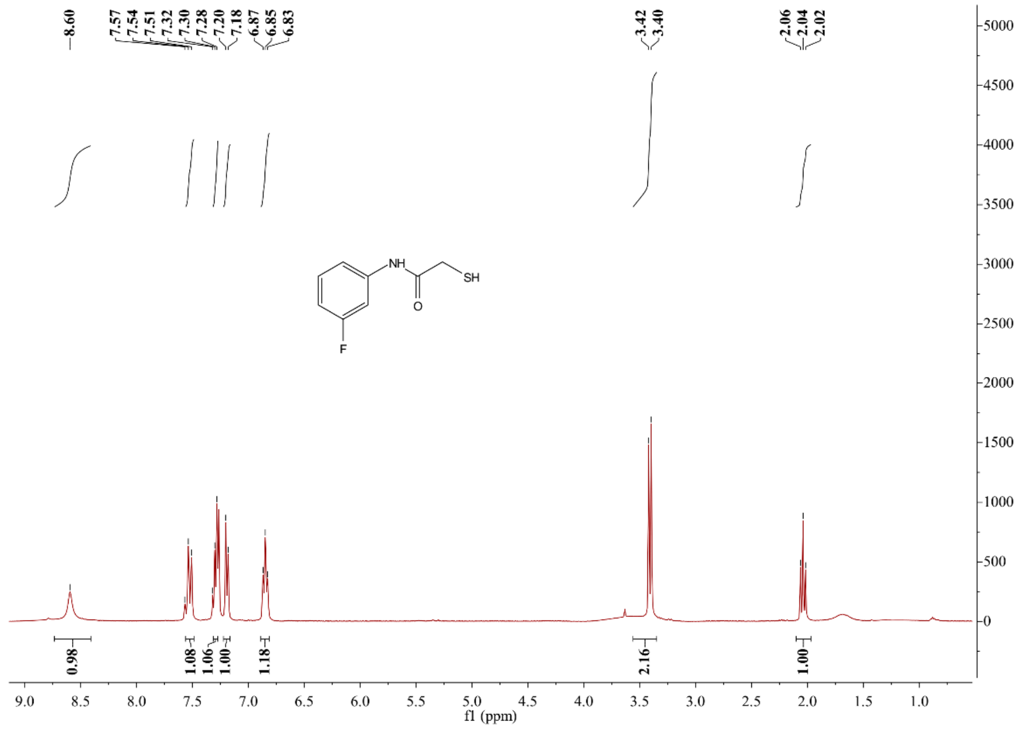

Figure S1. ^{1}H NMR spectrum of compound **2a**.

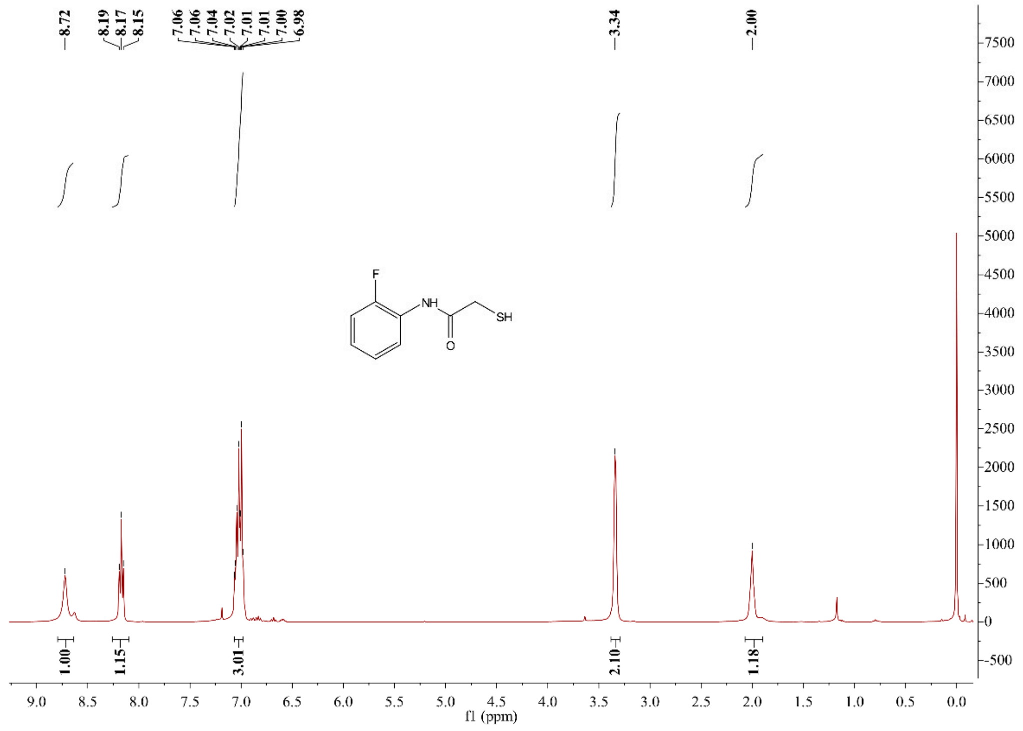

Figure S2. ^{1}H NMR spectrum of compound **2b**.

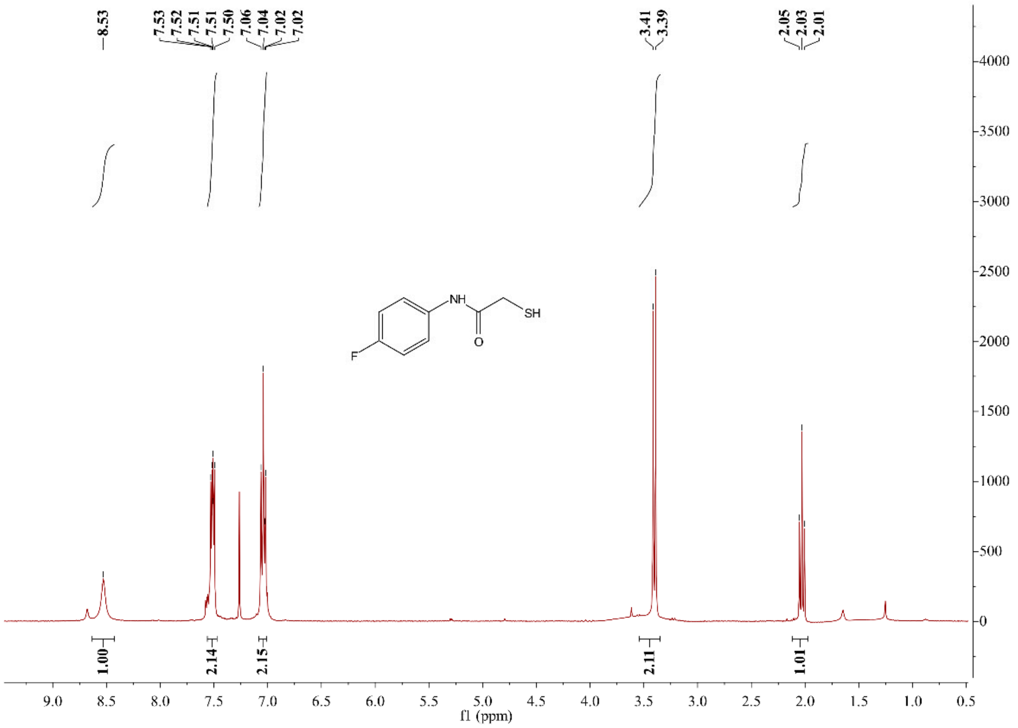

Figure S3. ^{1}H NMR spectrum of compound **2c**.

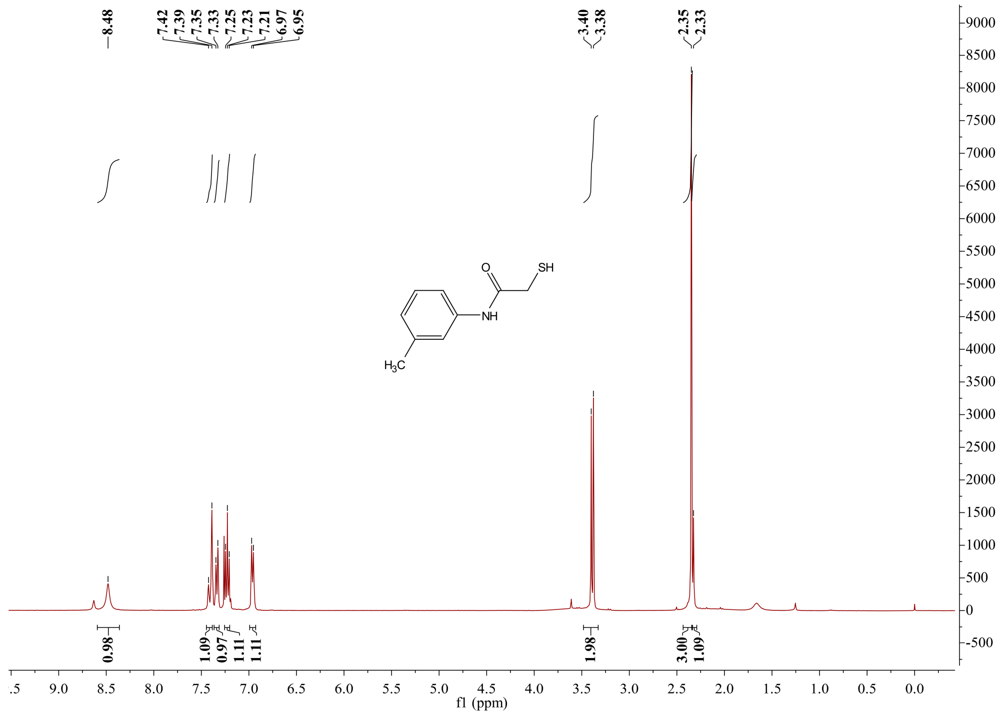

Figure S4. ^{1}H NMR spectrum of compound **2d**.

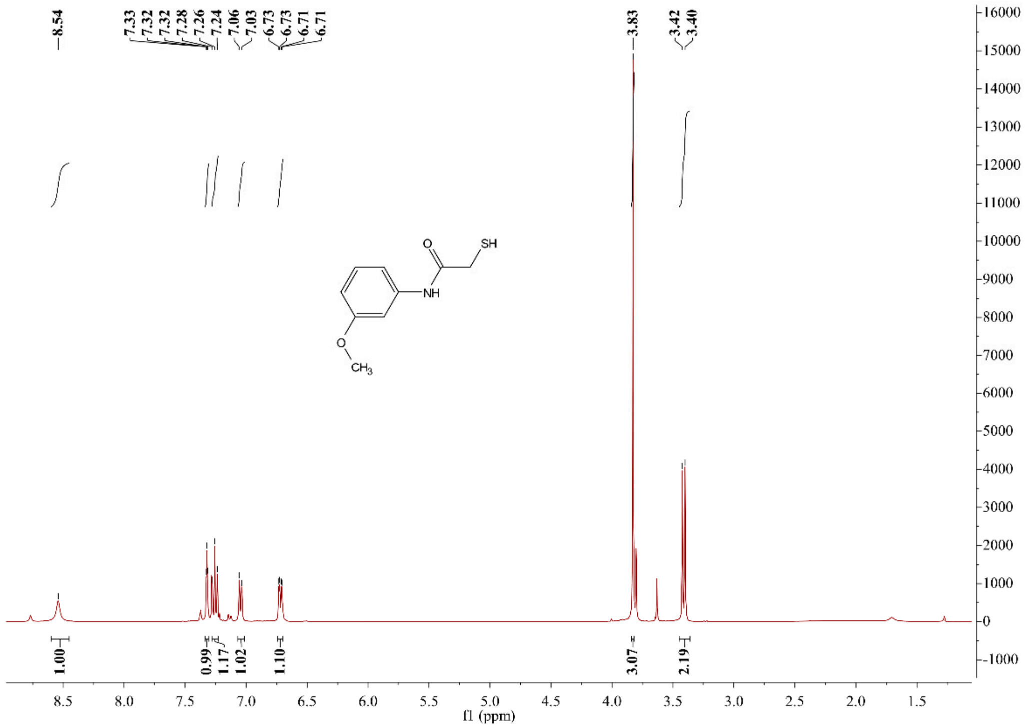

Figure S5. ^{1}H NMR spectrum of compound **2e**.

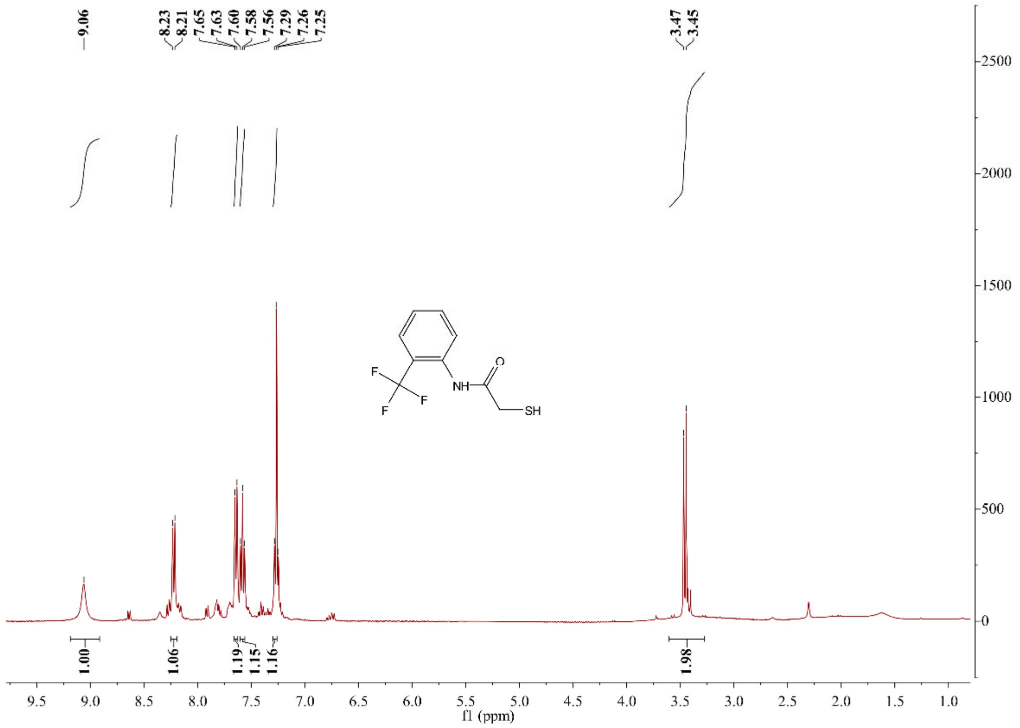

Figure S6. ^{1}H NMR spectrum of compound **2f**.

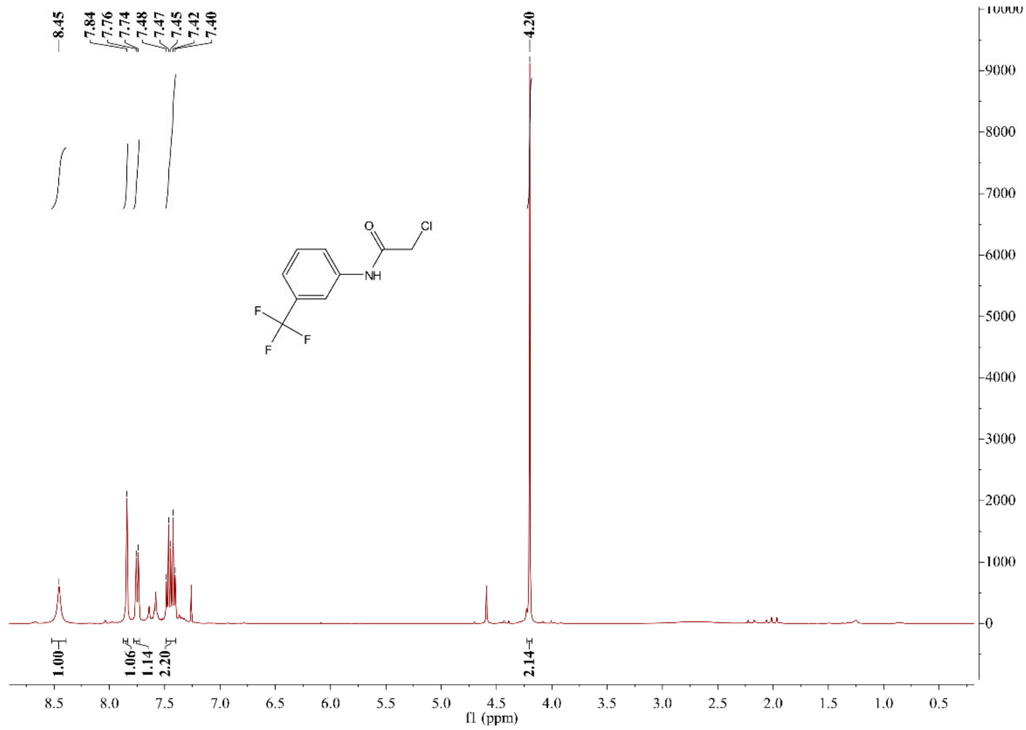

Figure S7. ^{1}H NMR spectrum of compound **2g**.

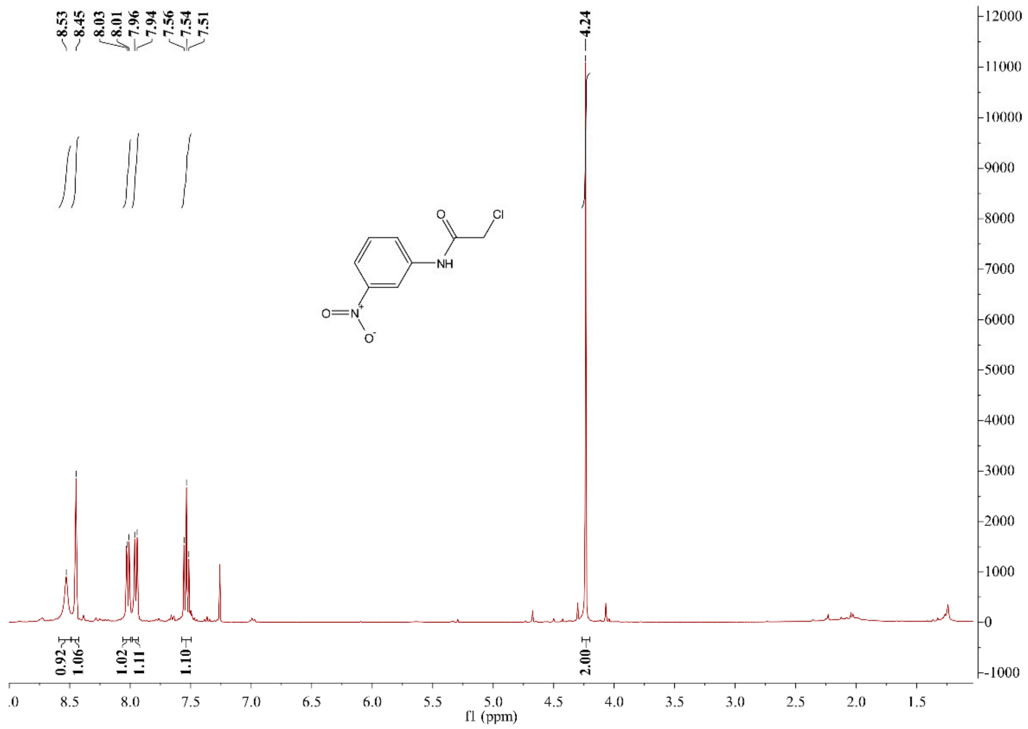

Figure S8. ^{1}H NMR spectrum of compound **2h**.

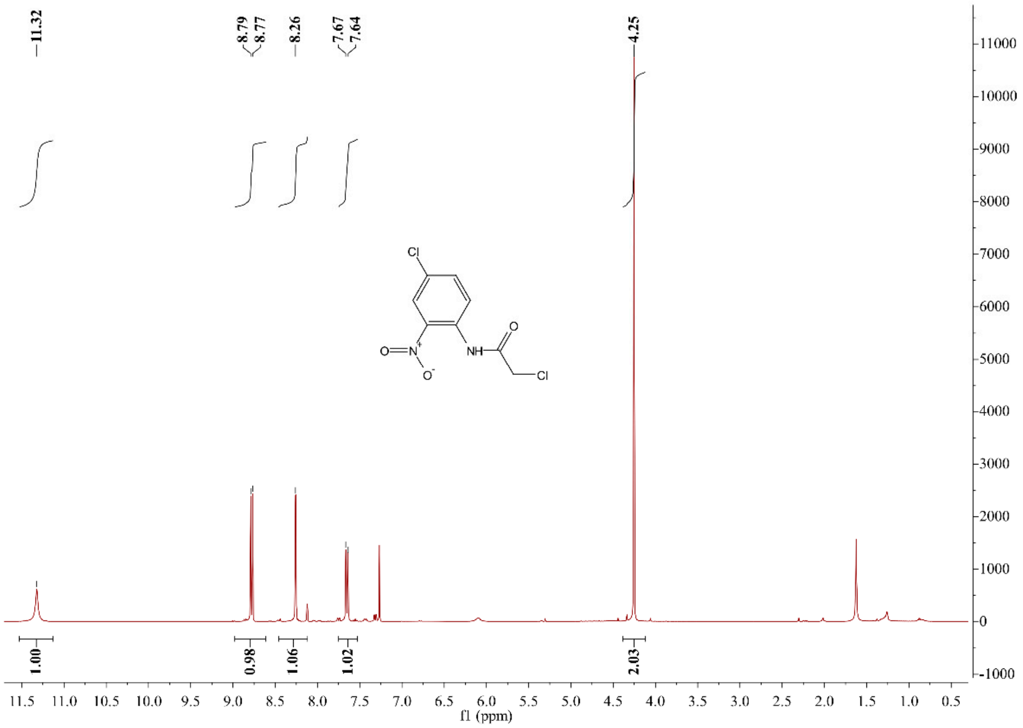

Figure S9. ^{1}H NMR spectrum of compound **2i**.

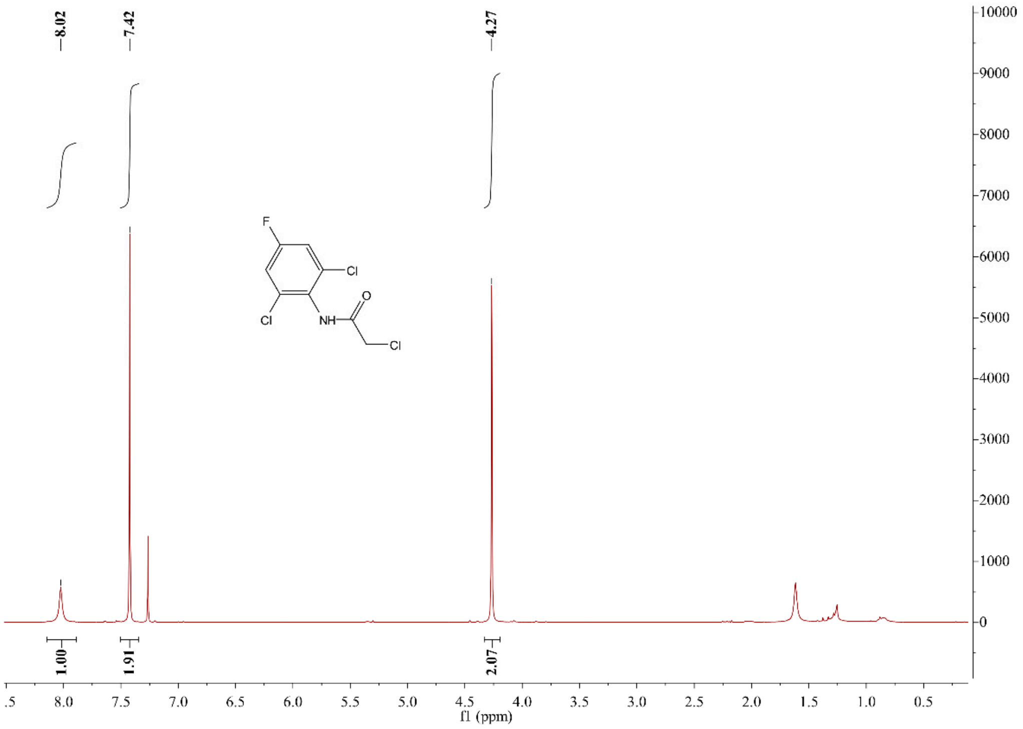

Figure S10. ^{1}H NMR spectrum of compound **2j**.

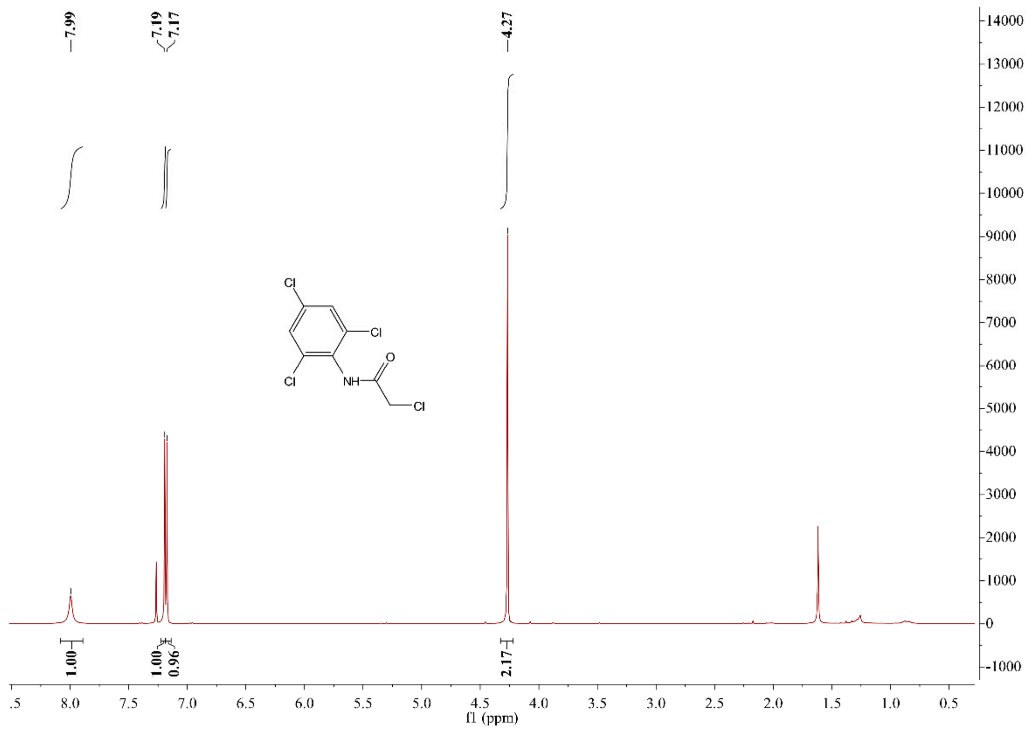

Figure S11. ^1H NMR spectrum of compound **2k**.

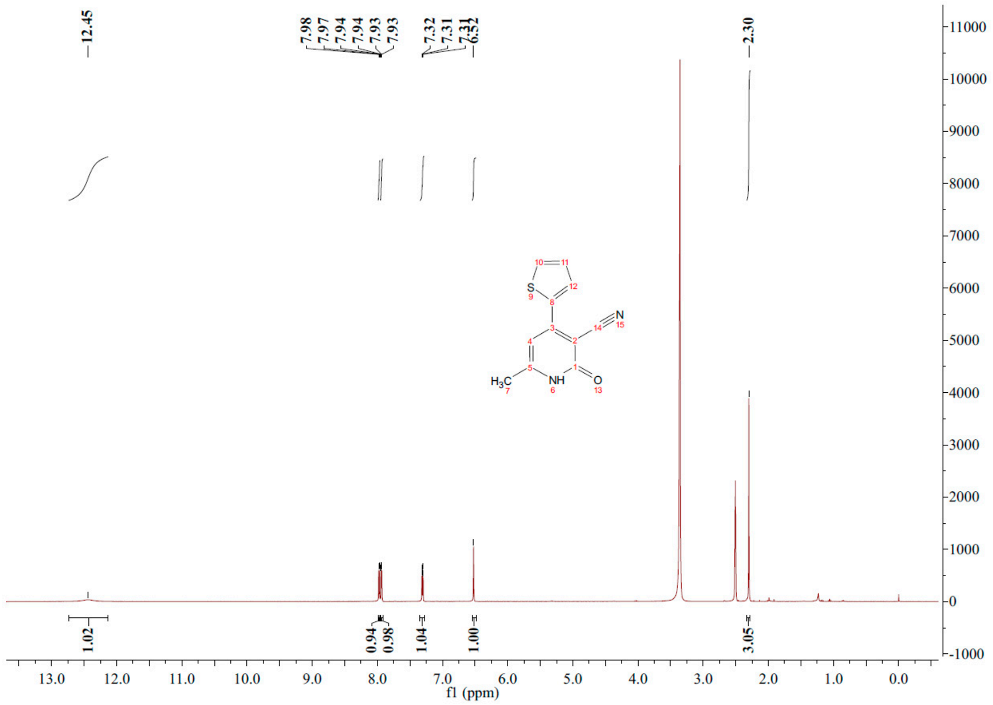

Figure S12. ^1H NMR spectrum of compound **6a**.

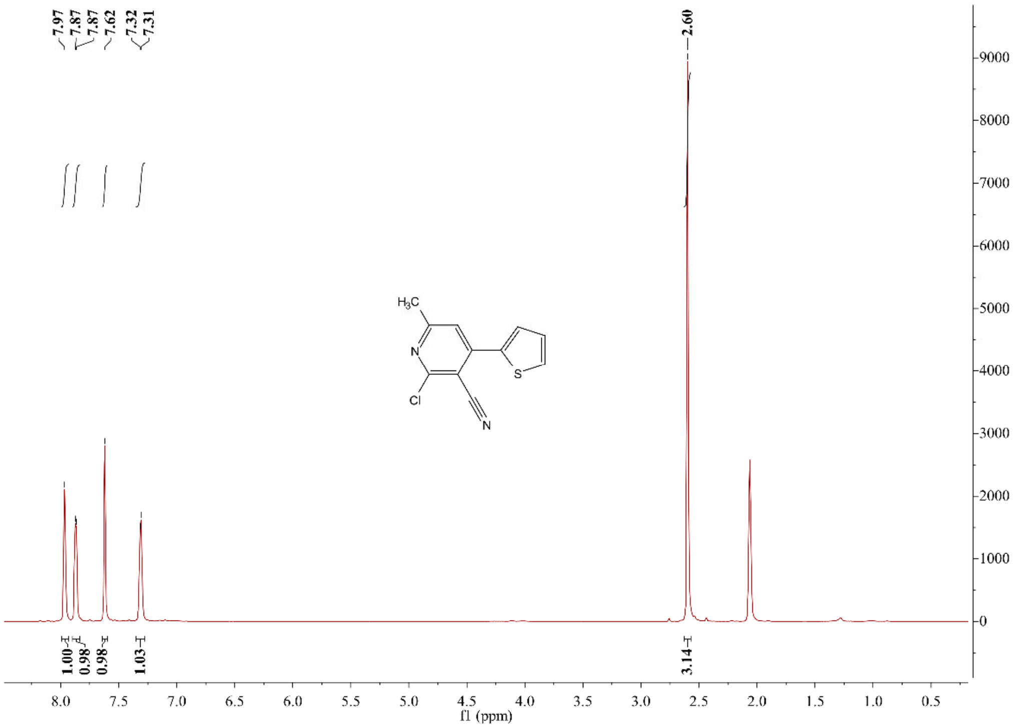

Figure S13. ^{1}H NMR spectrum of compound **7a**.

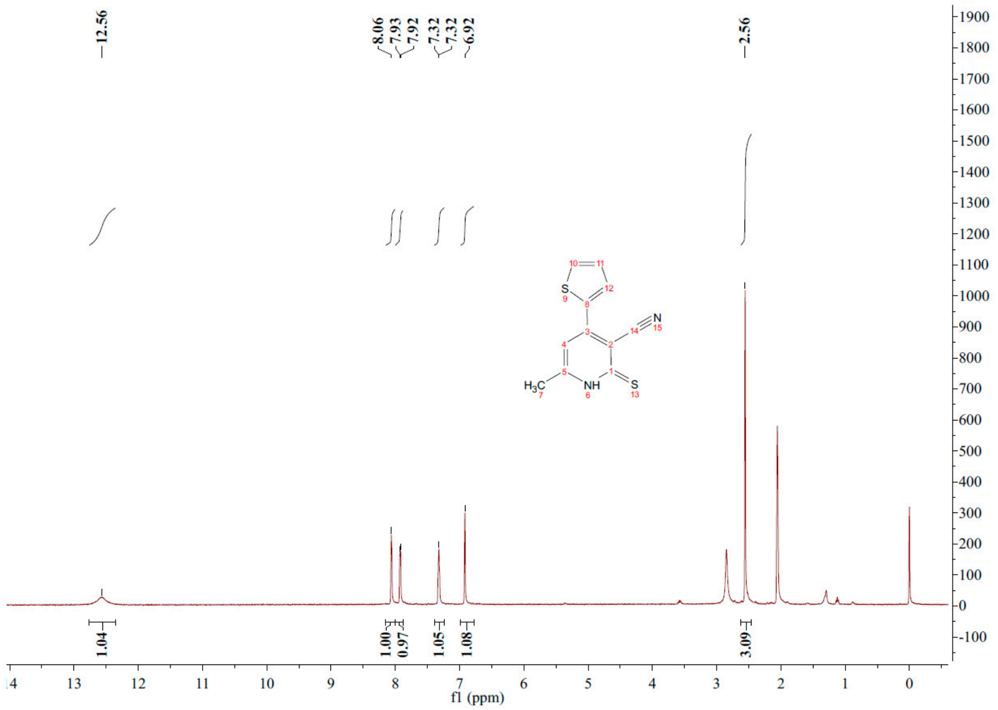

Figure S14. ^{1}H NMR spectrum of compound **8**.

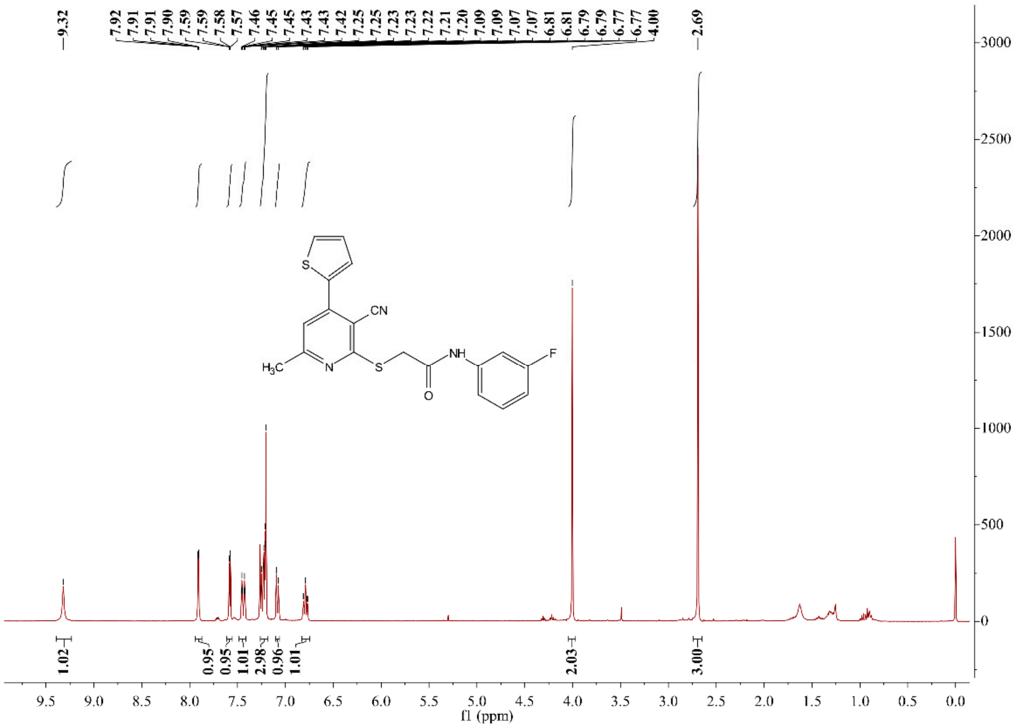

Figure S15. ^{1}H NMR spectrum of compound **A**.

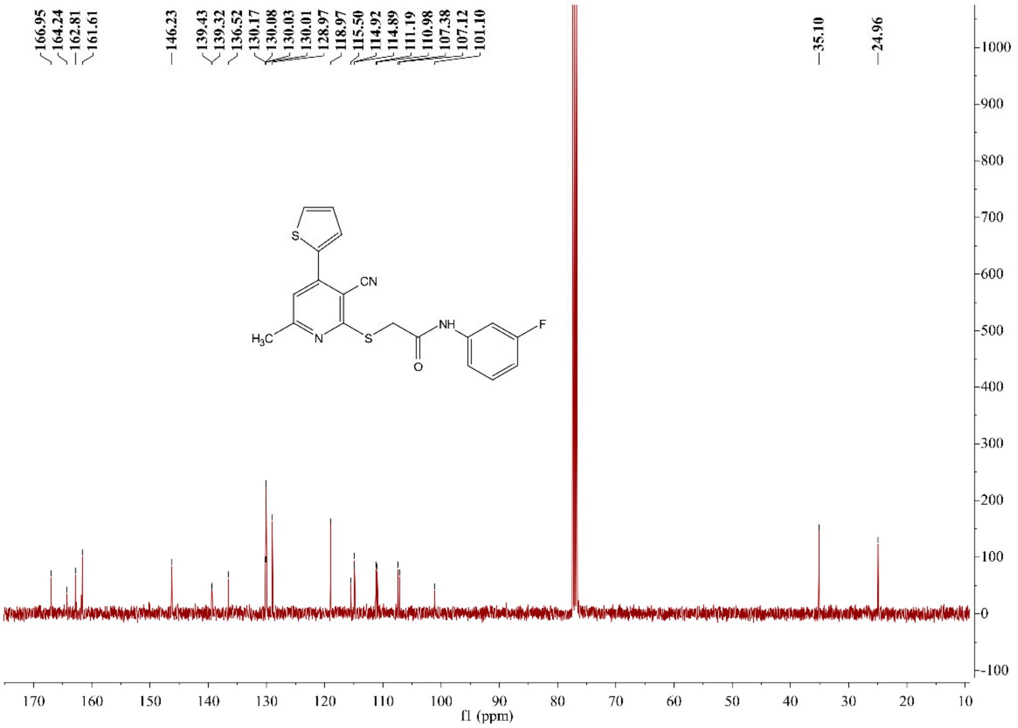

Figure S16. ^{13}C NMR spectrum of compound **A**.

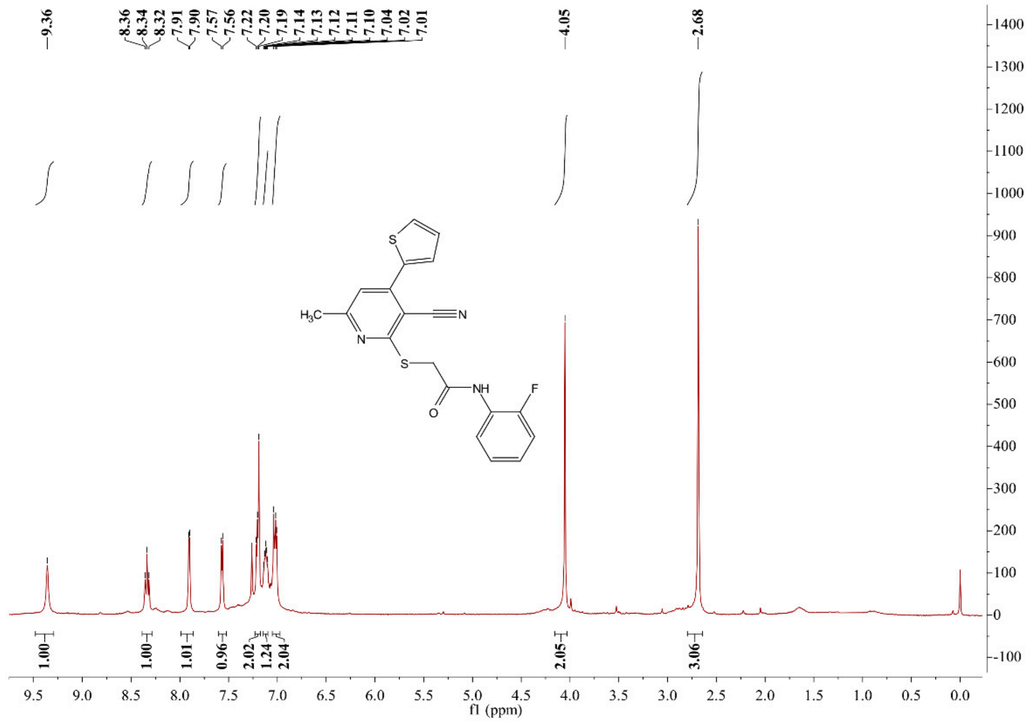

Figure S17. ^{1}H NMR spectrum of compound **Ia**.

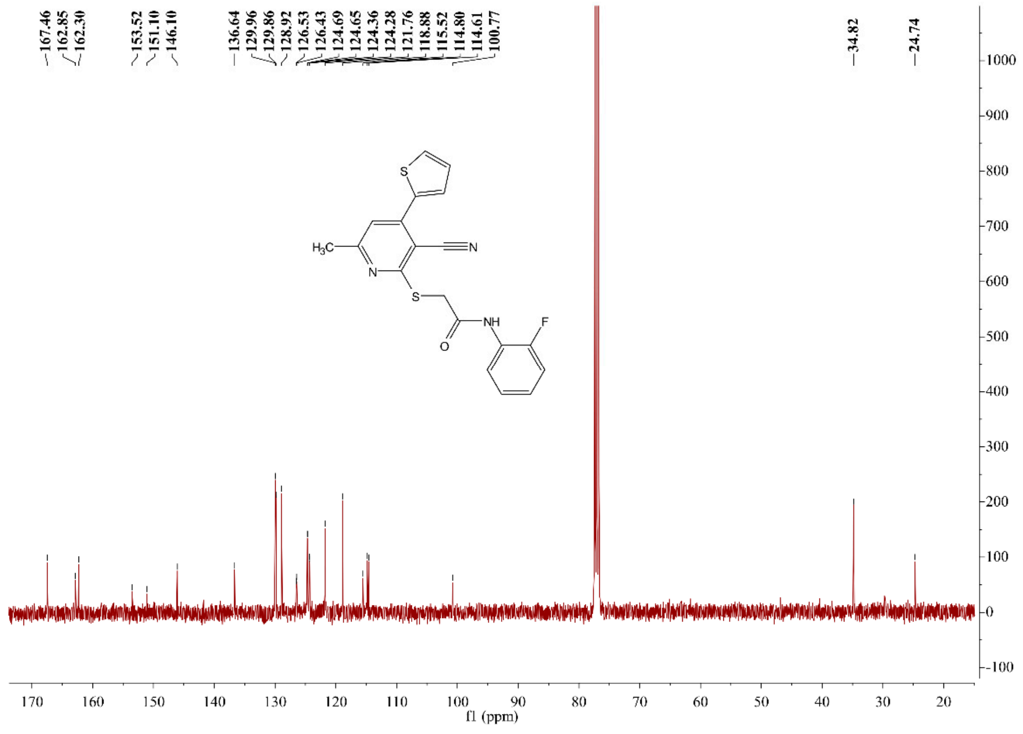

Figure S18. ^{13}C NMR spectrum of compound **Ia**.

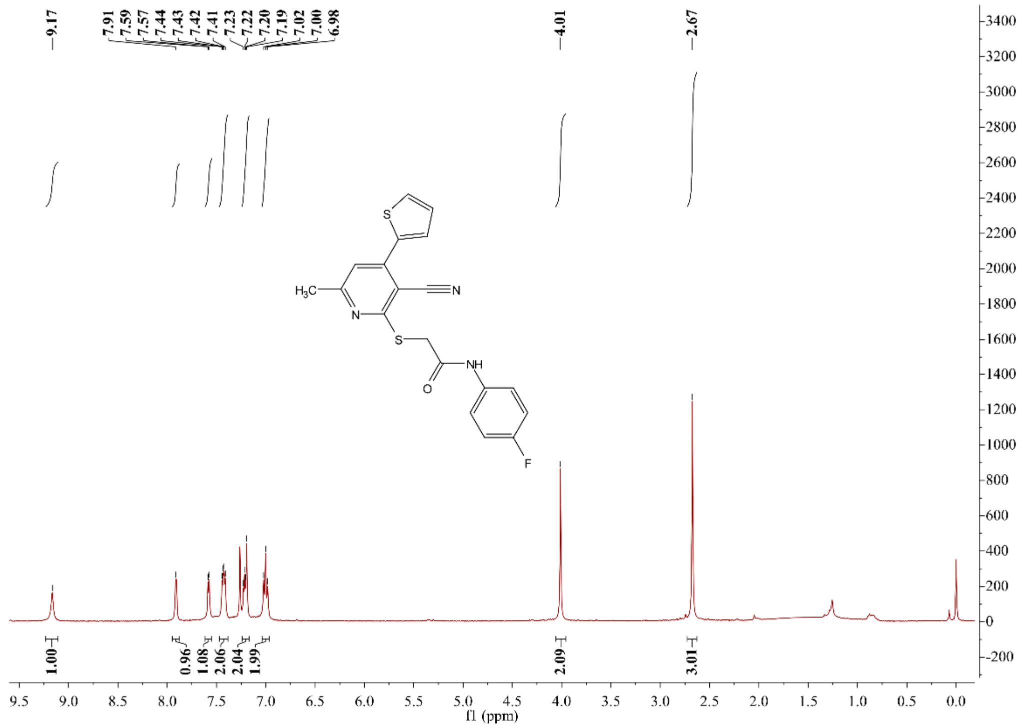

Figure S19. ^{1}H NMR spectrum of compound **Ib**.

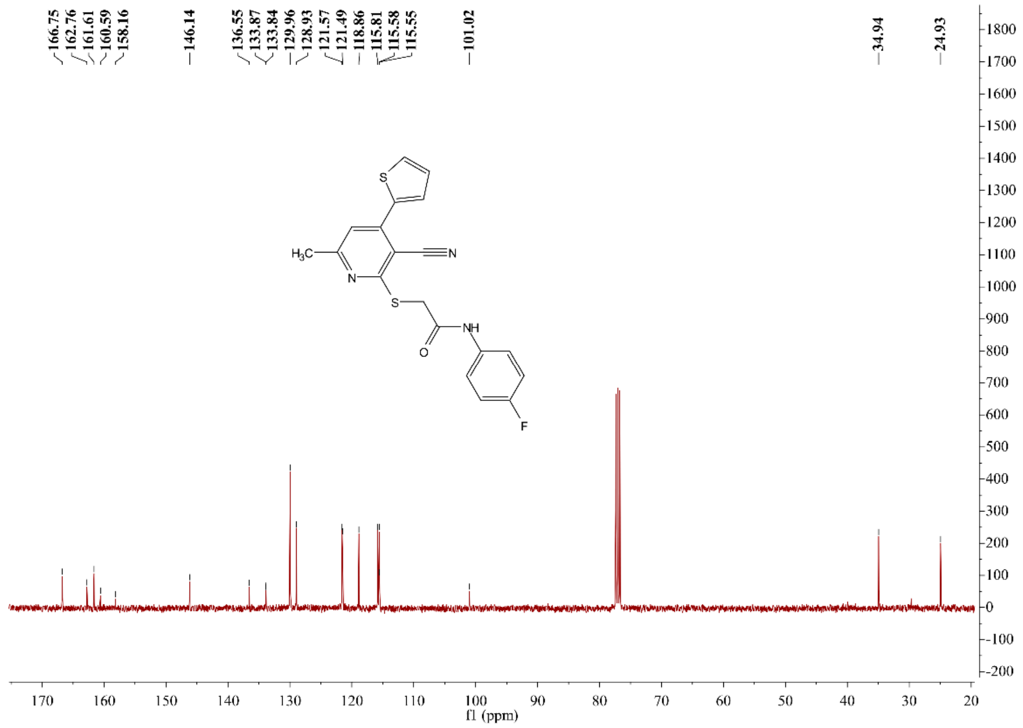

Figure S20. ^{13}C NMR spectrum of compound **Ib**.

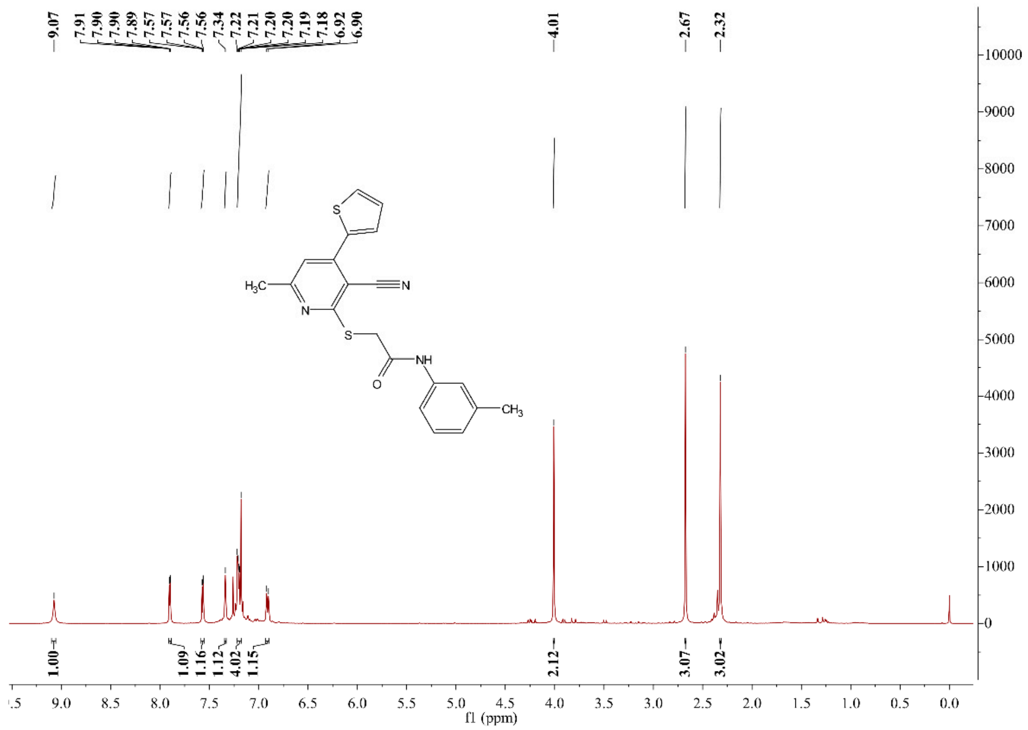

Figure S21. ^{1}H NMR spectrum of compound **Ic**.

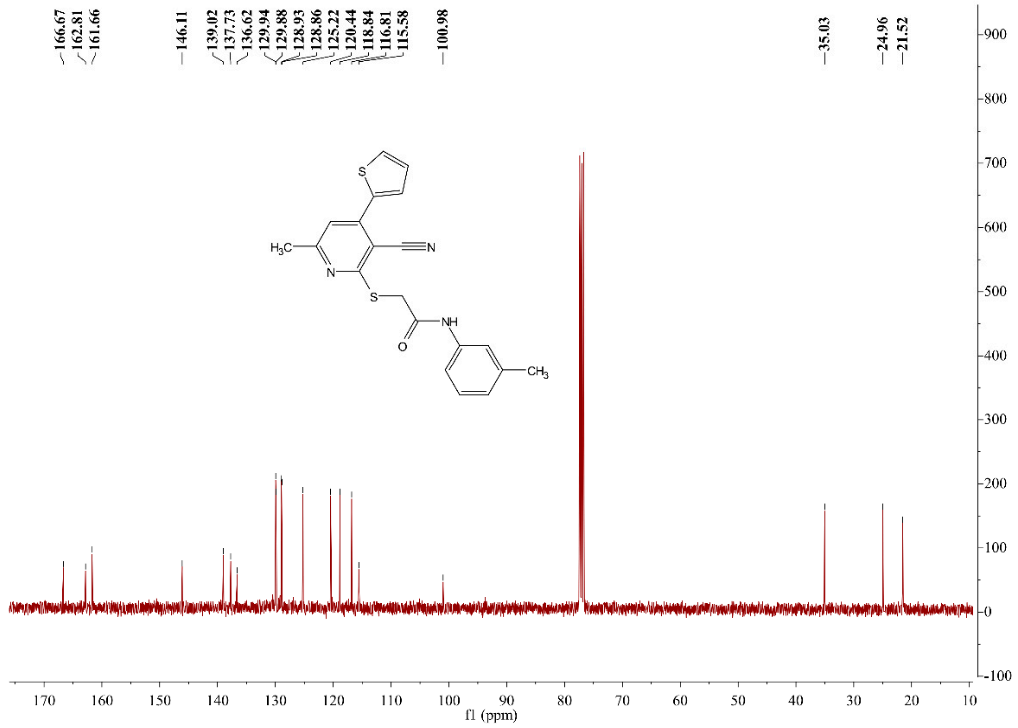

Figure S22. ^{13}C NMR spectrum of compound **Ic**.

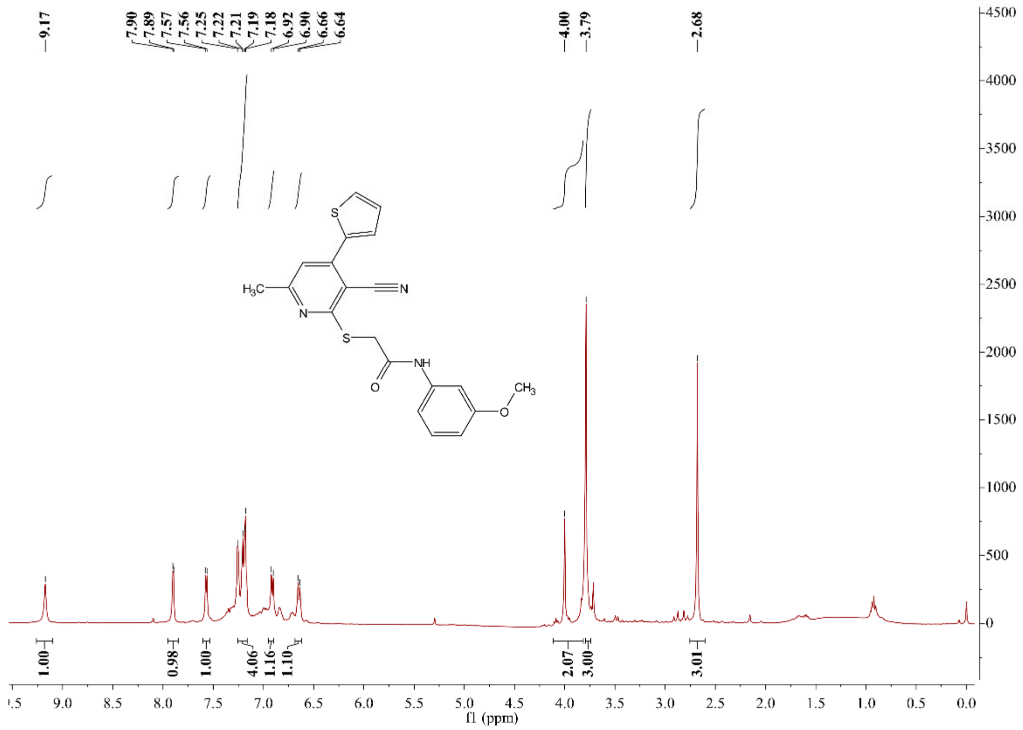

Figure S23. ^{1}H NMR spectrum of compound **Id**.

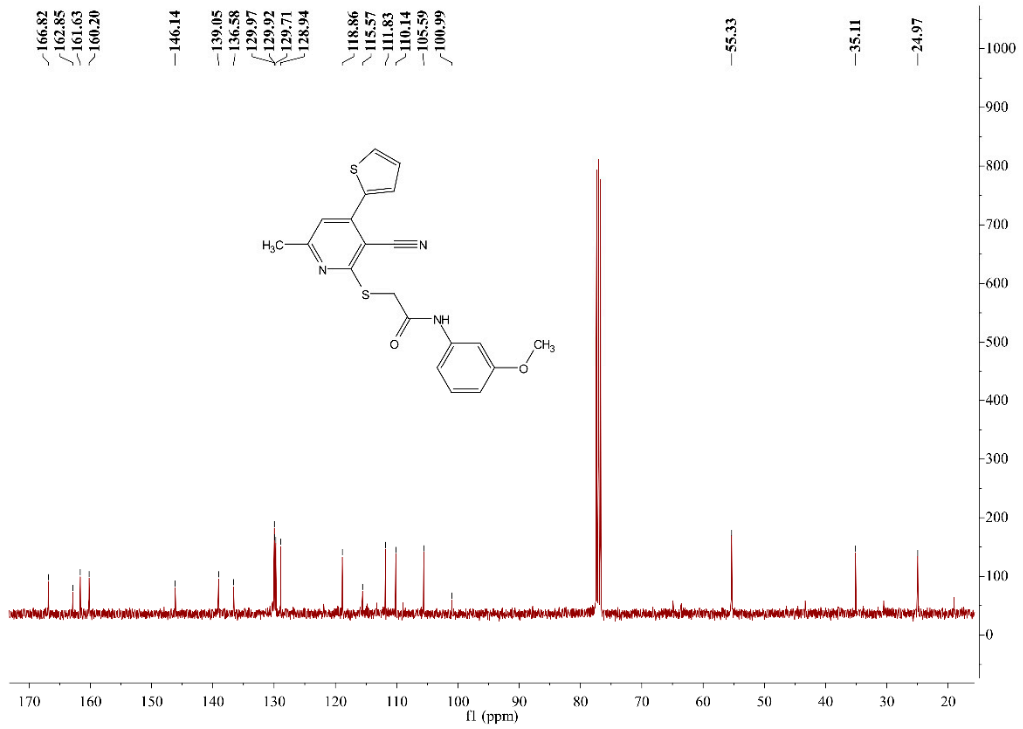

Figure S24. ^{13}C NMR spectrum of compound **Id**.

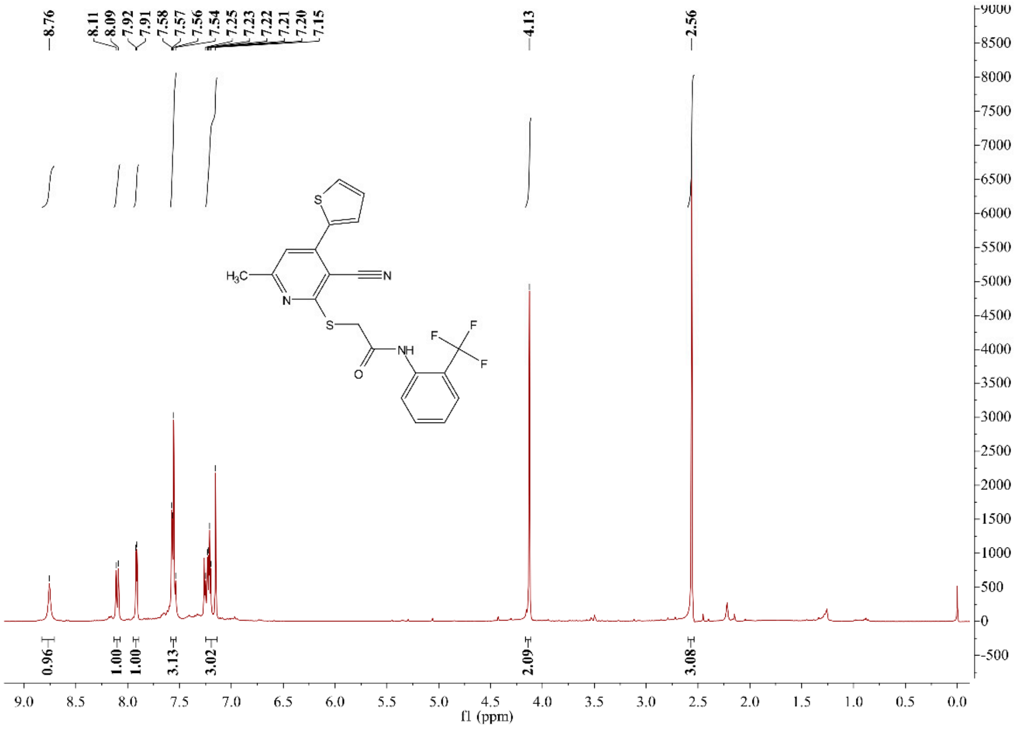

Figure S25. ^{1}H NMR spectrum of compound **Ie**.

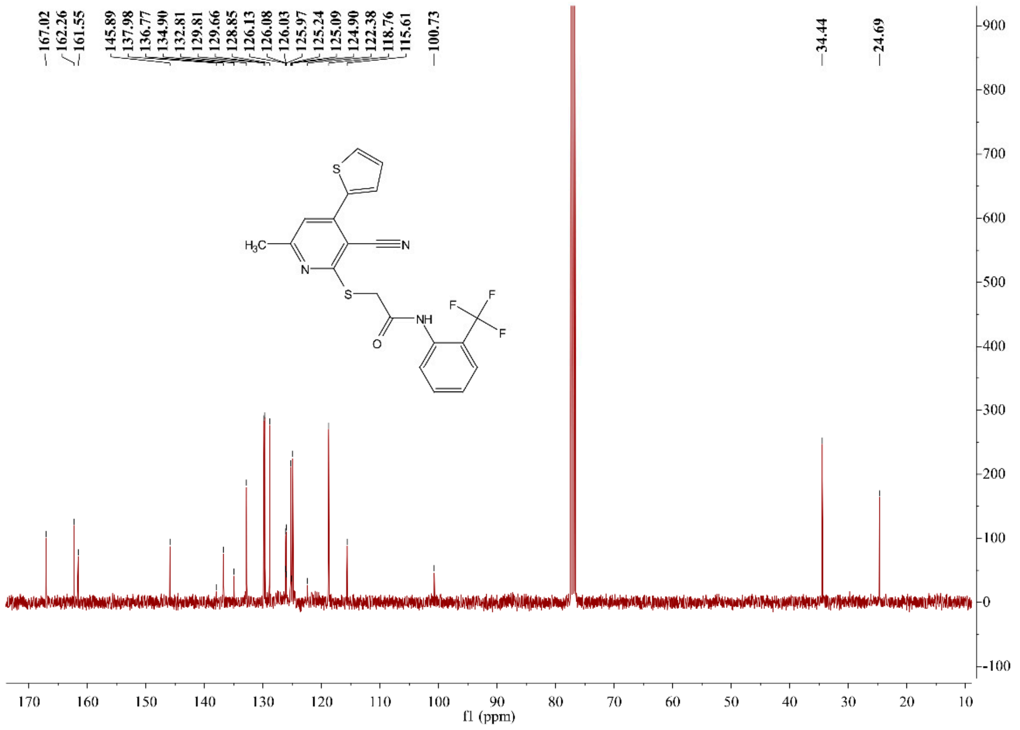

Figure S26. ^{13}C NMR spectrum of compound **Ie**.

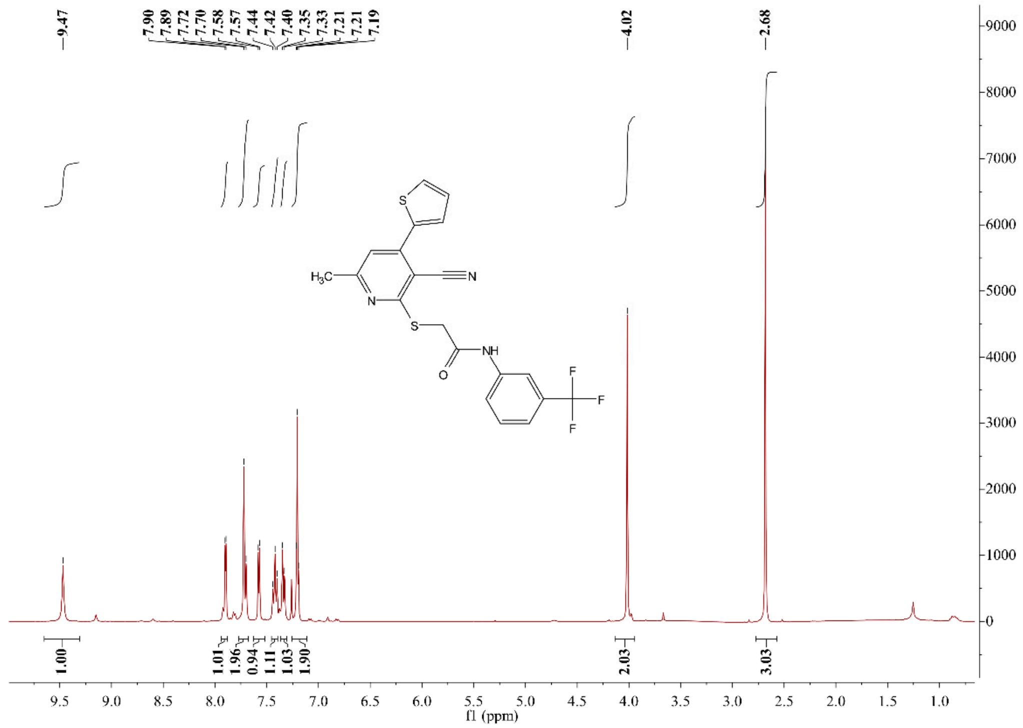

Figure S27. ^{1}H NMR spectrum of compound **If**.

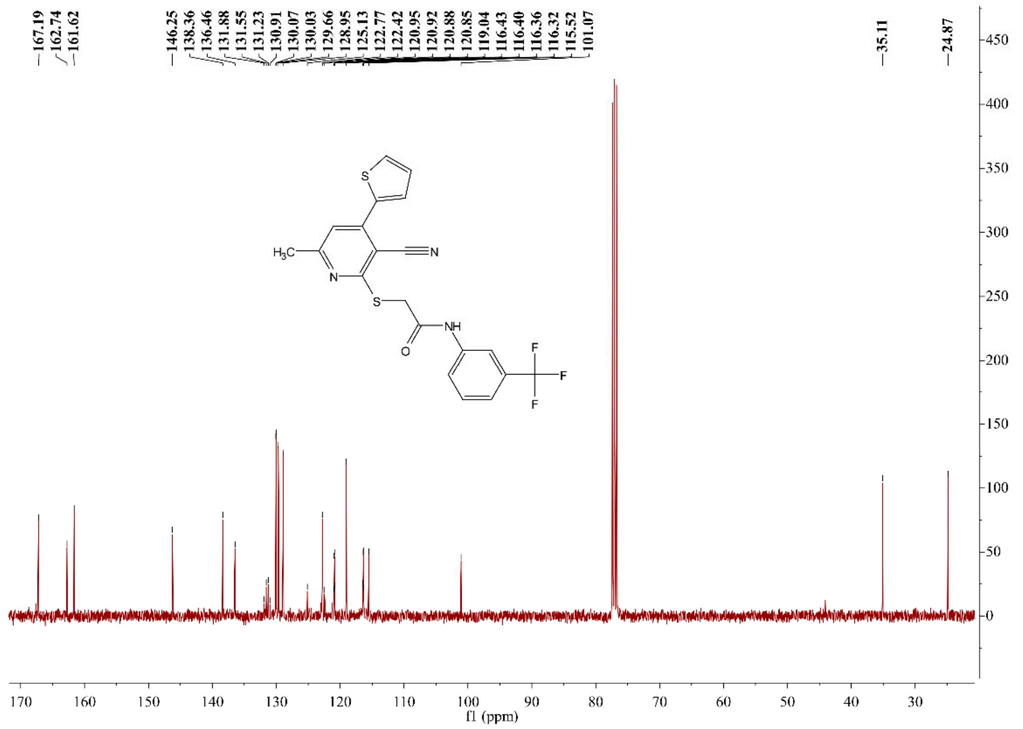

Figure S28. ^{13}C NMR spectrum of compound **If**.

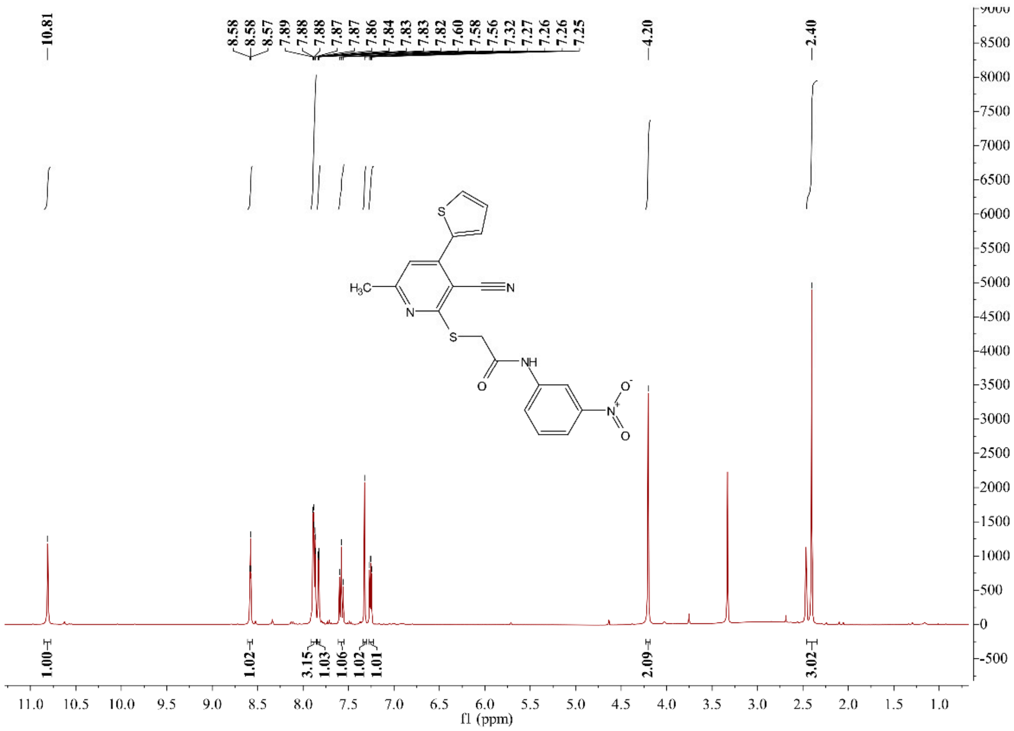

Figure S29. ^{1}H NMR spectrum of compound **Ig**.

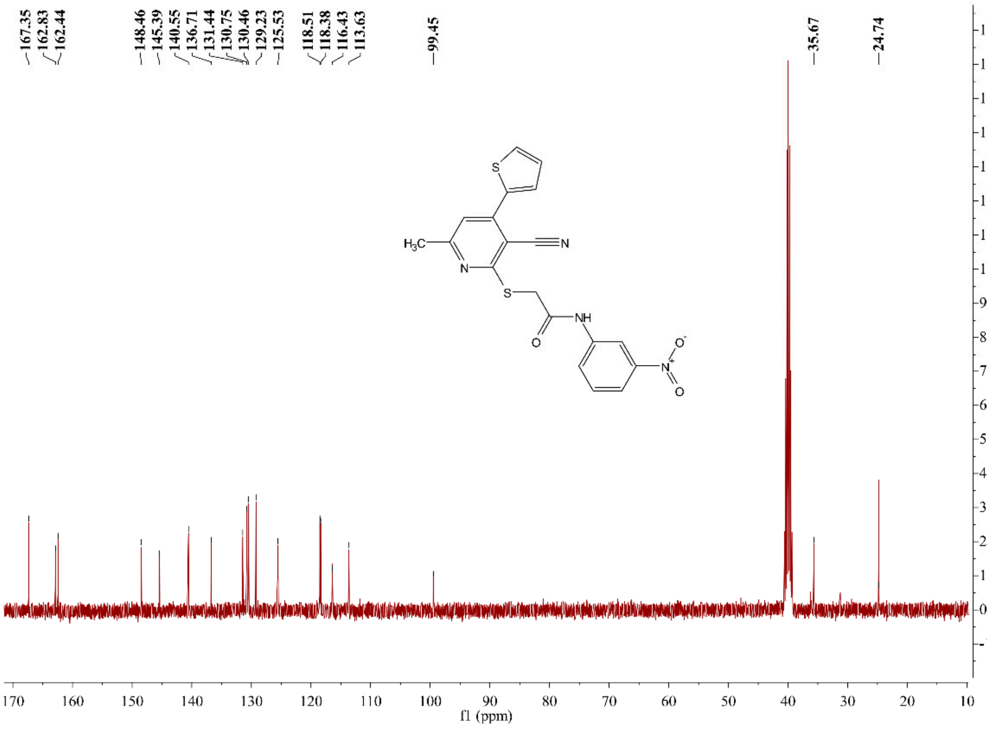

Figure S30. ^{13}C NMR spectrum of compound **Ig**.

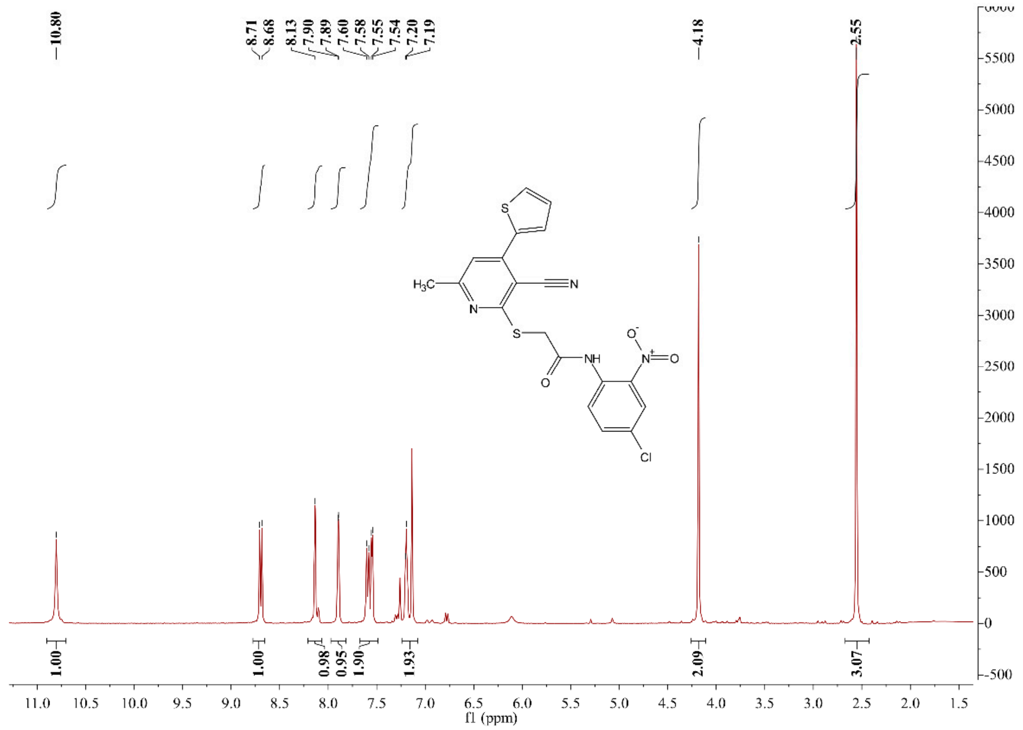

Figure S31. ^{1}H NMR spectrum of compound **Ih**.

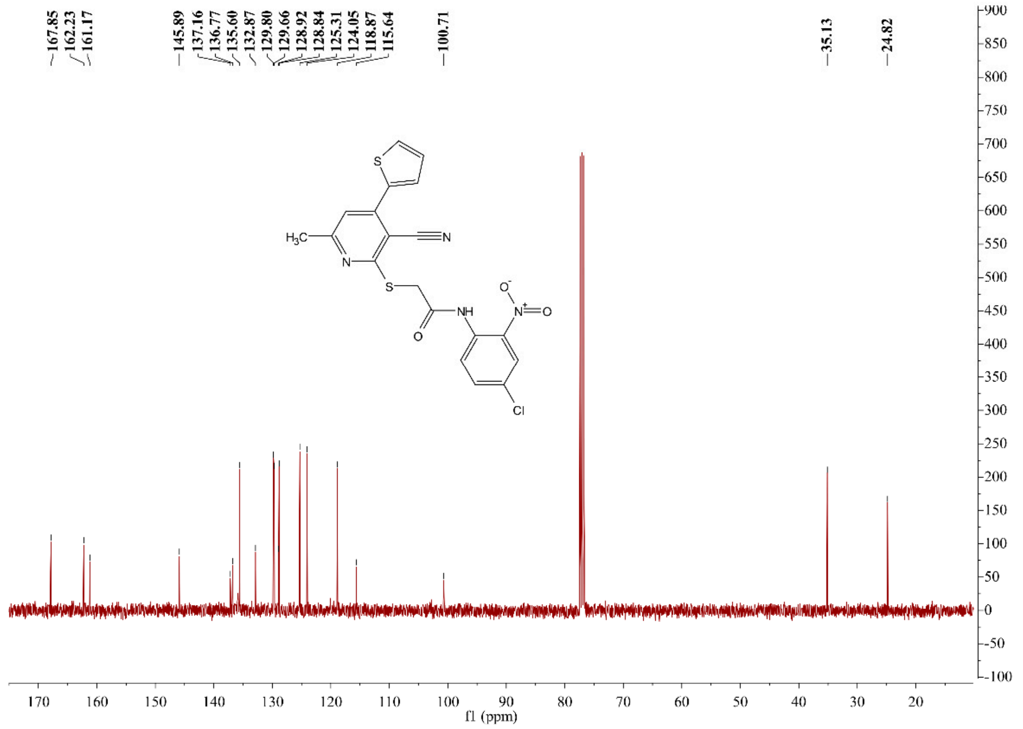

Figure S32. ^{13}C NMR spectrum of compound **Ih**.

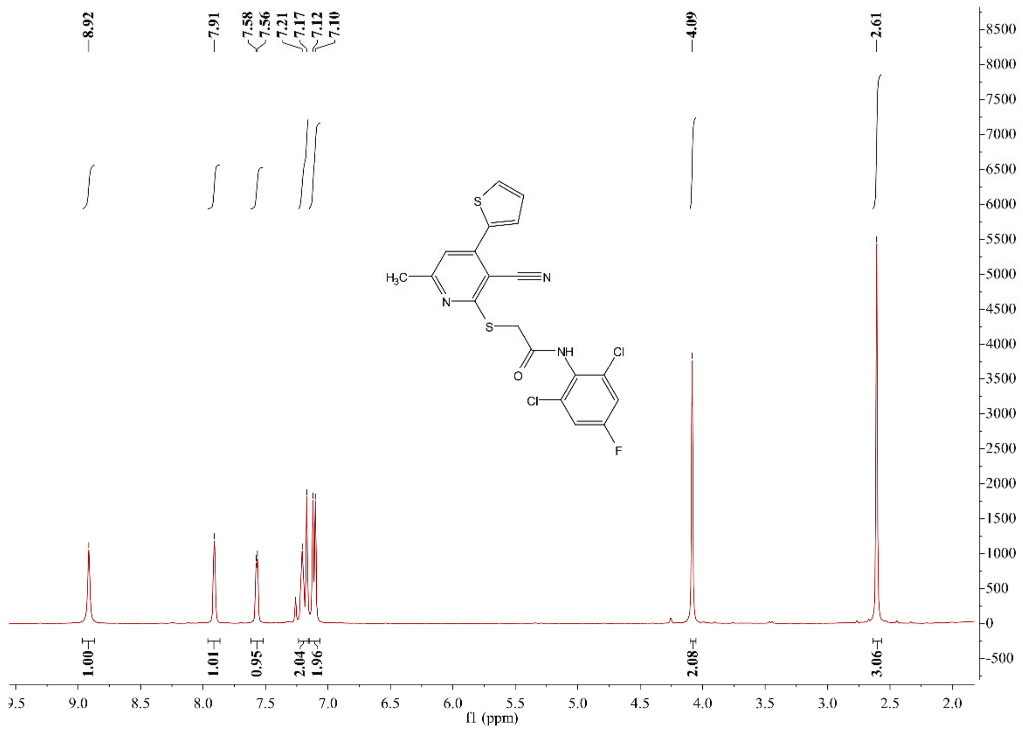

Figure S33. ^{1}H NMR spectrum of compound **Ii**.

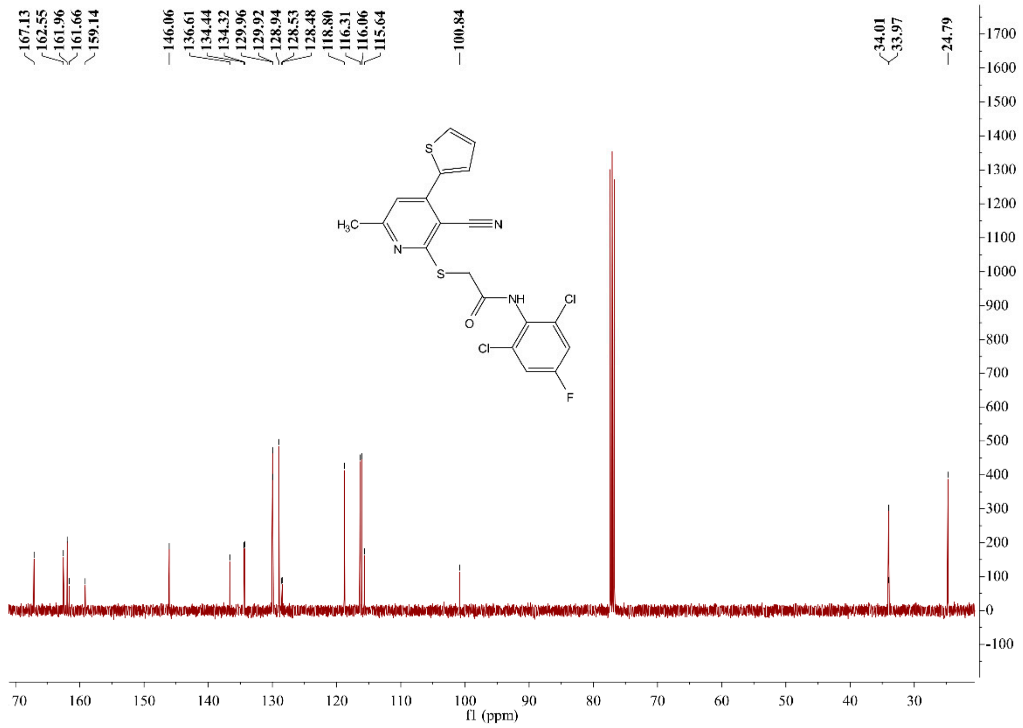

Figure S34. ^{13}C NMR spectrum of compound **Ii**.

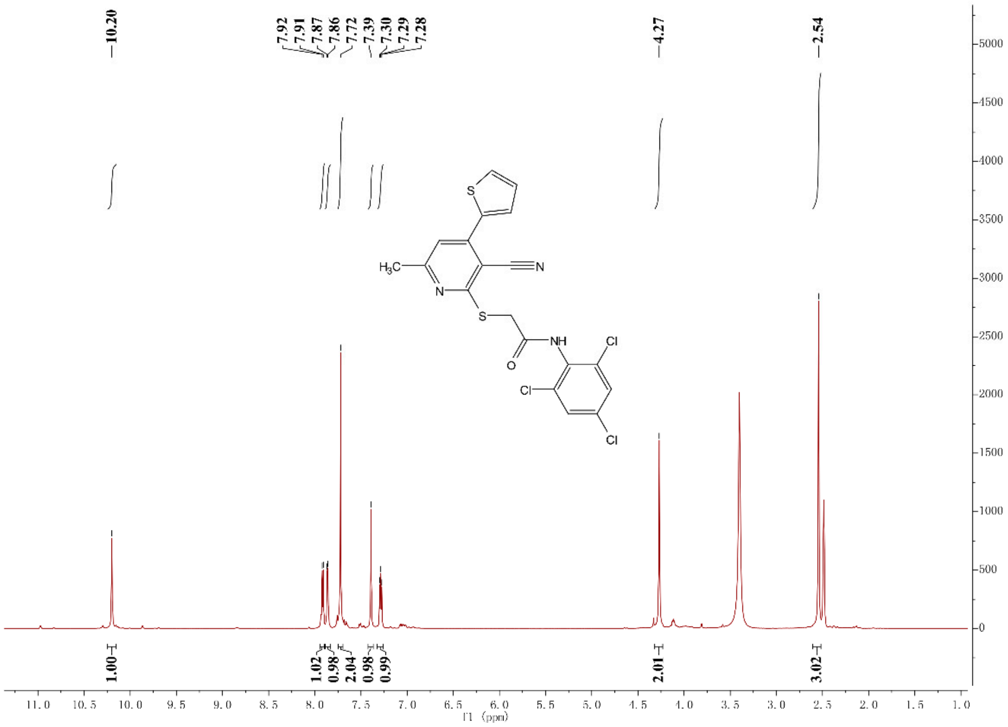

Figure S35. ^{1}H NMR spectrum of compound **Ij**.

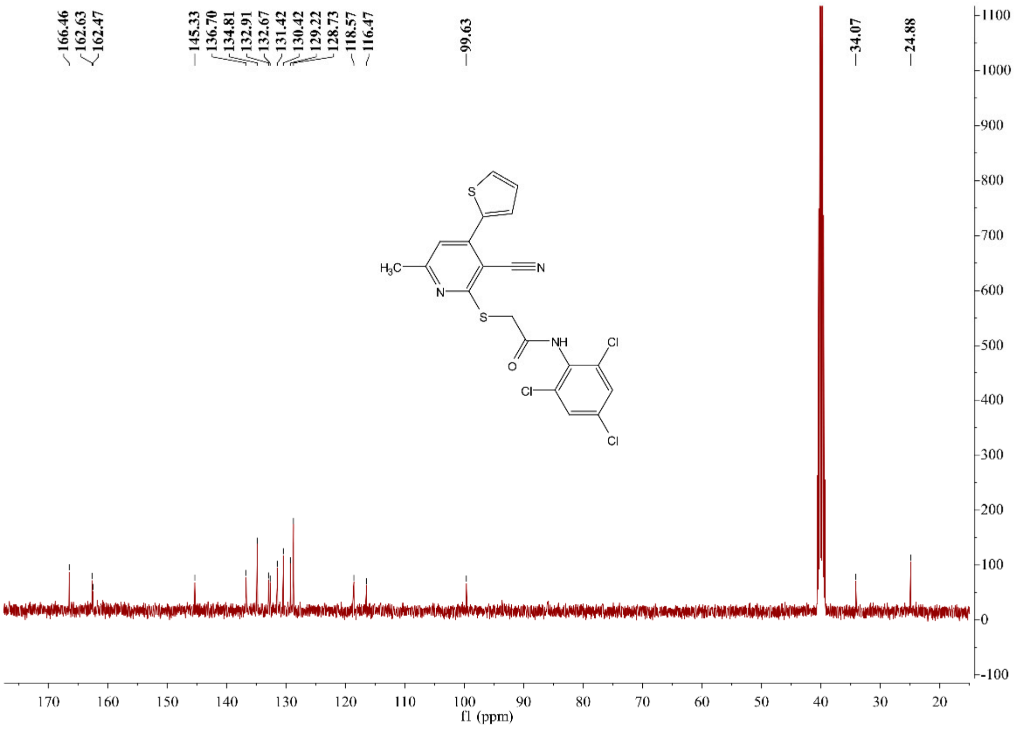

Figure S36. ^{13}C NMR spectrum of compound **Ij**.

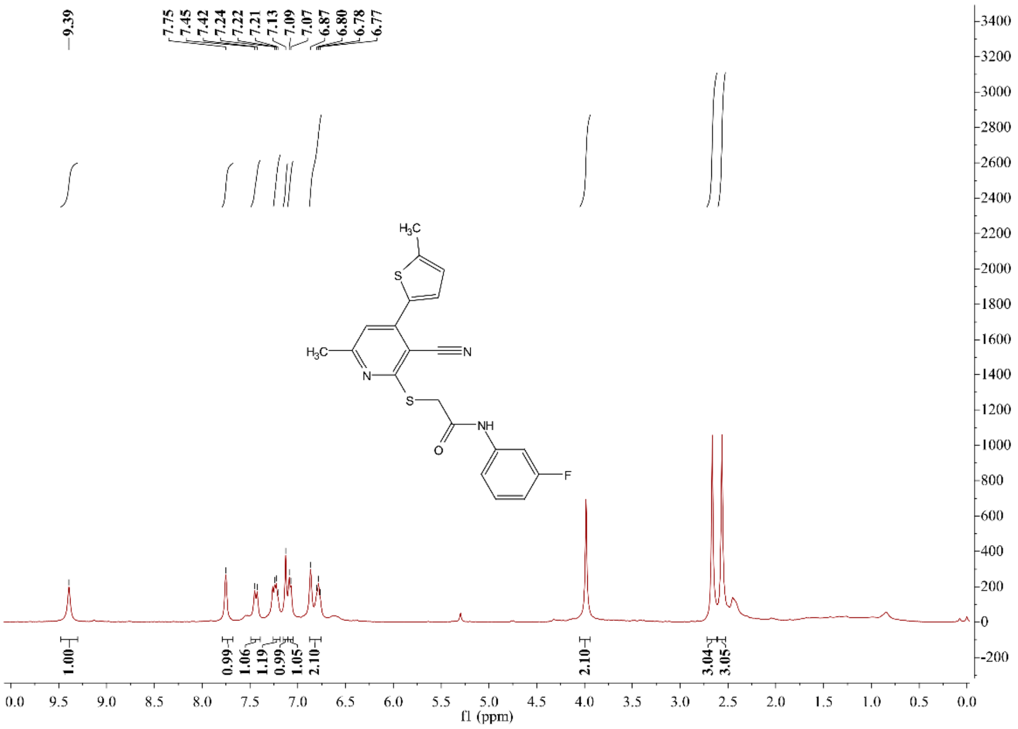

Figure S37. ^{1}H NMR spectrum of compound **Ik**.

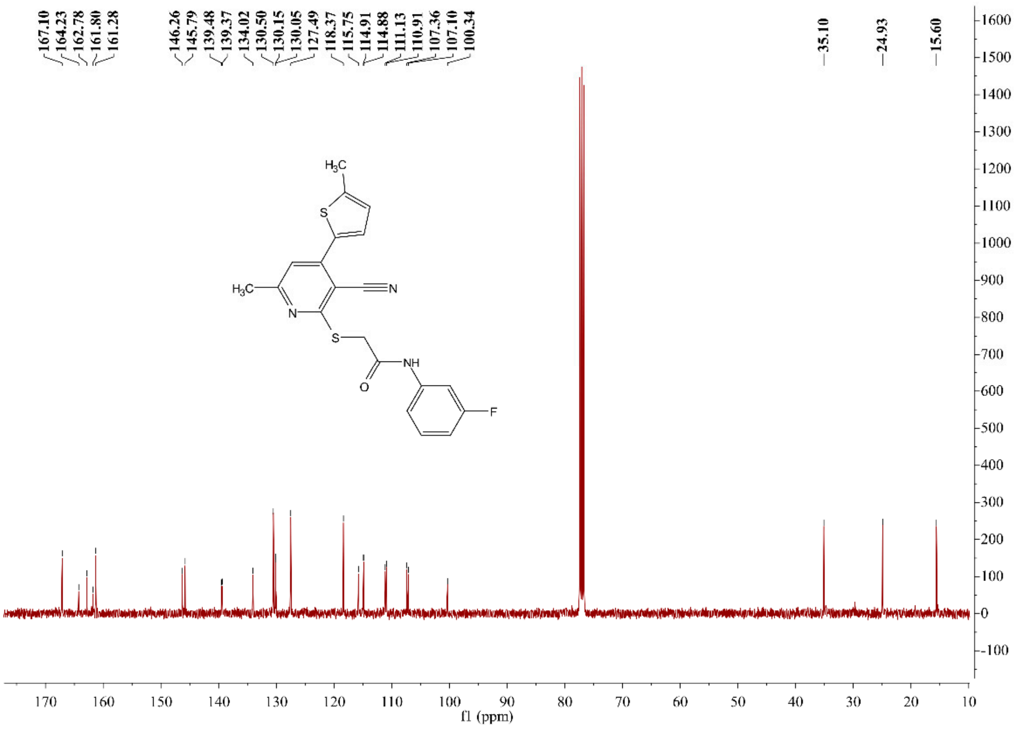

Figure S38. ^{13}C NMR spectrum of compound **Ik**.

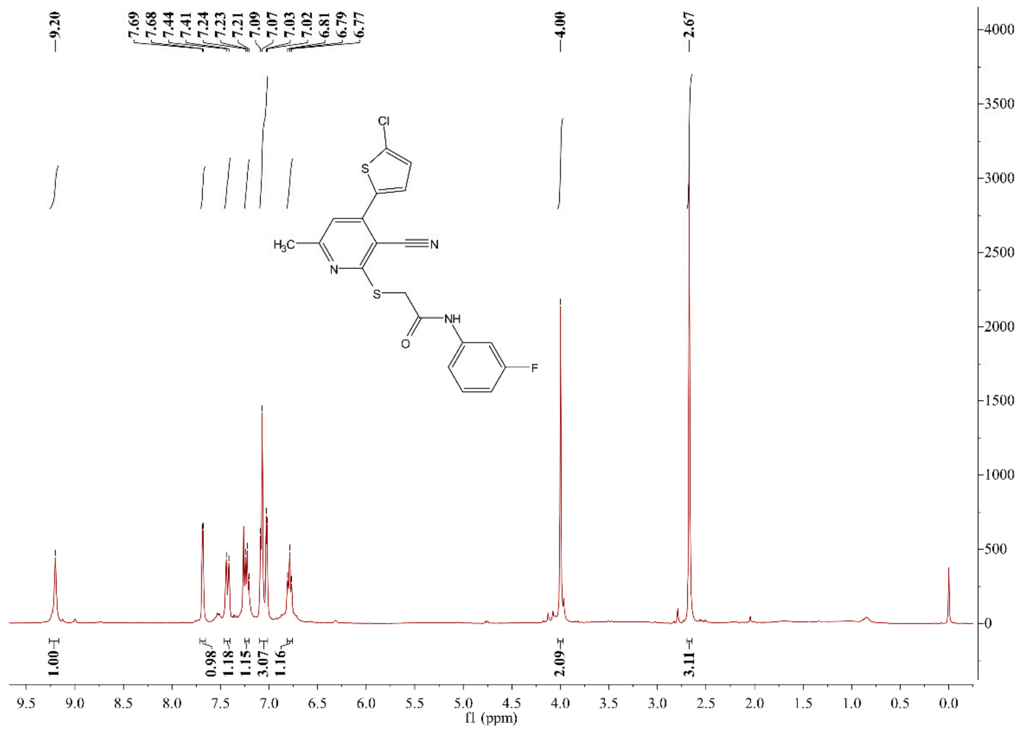

Figure S39. ^{1}H NMR spectrum of compound **Il**.

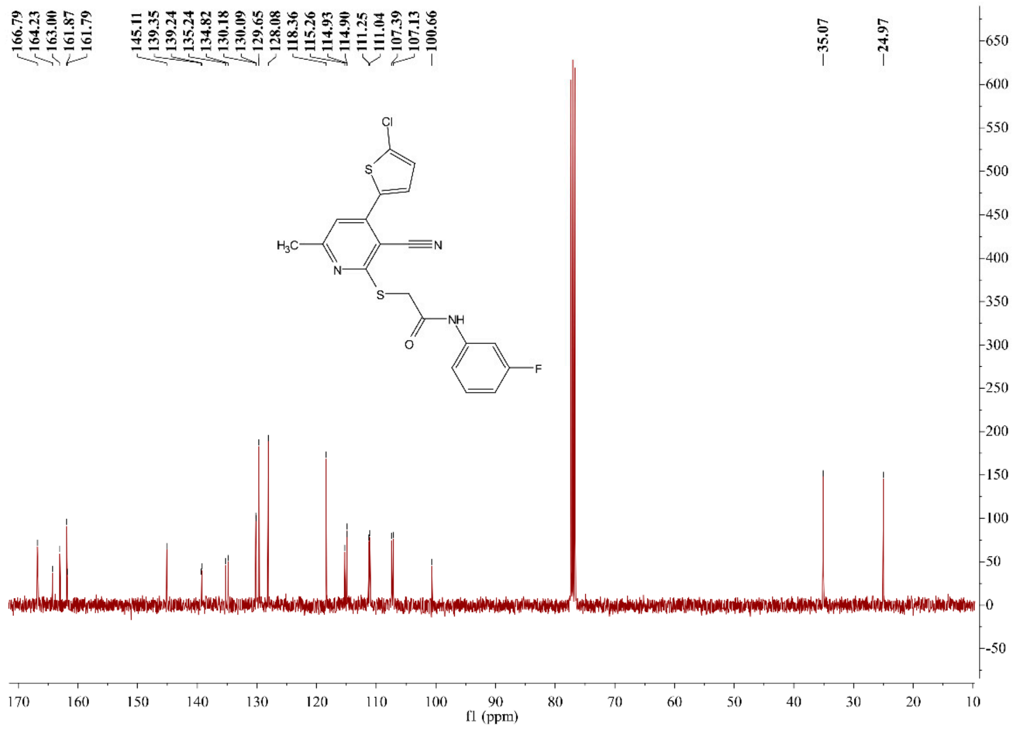

Figure S40. ^{13}C NMR spectrum of compound **Il**.

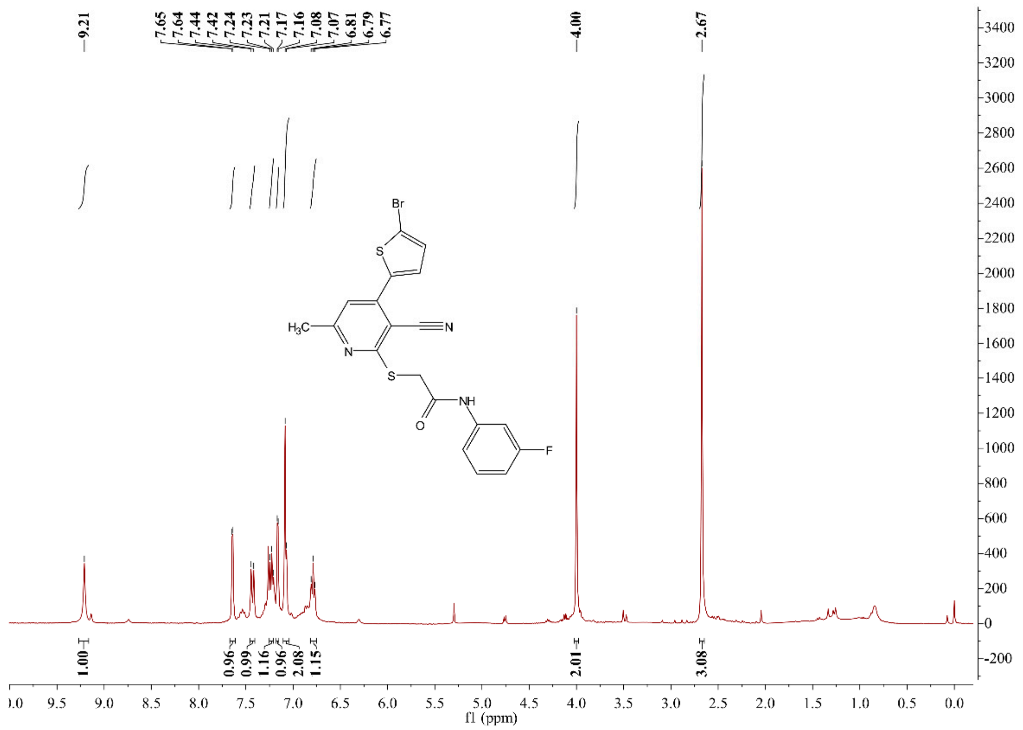

Figure S41. ^{1}H NMR spectrum of compound **Im**.

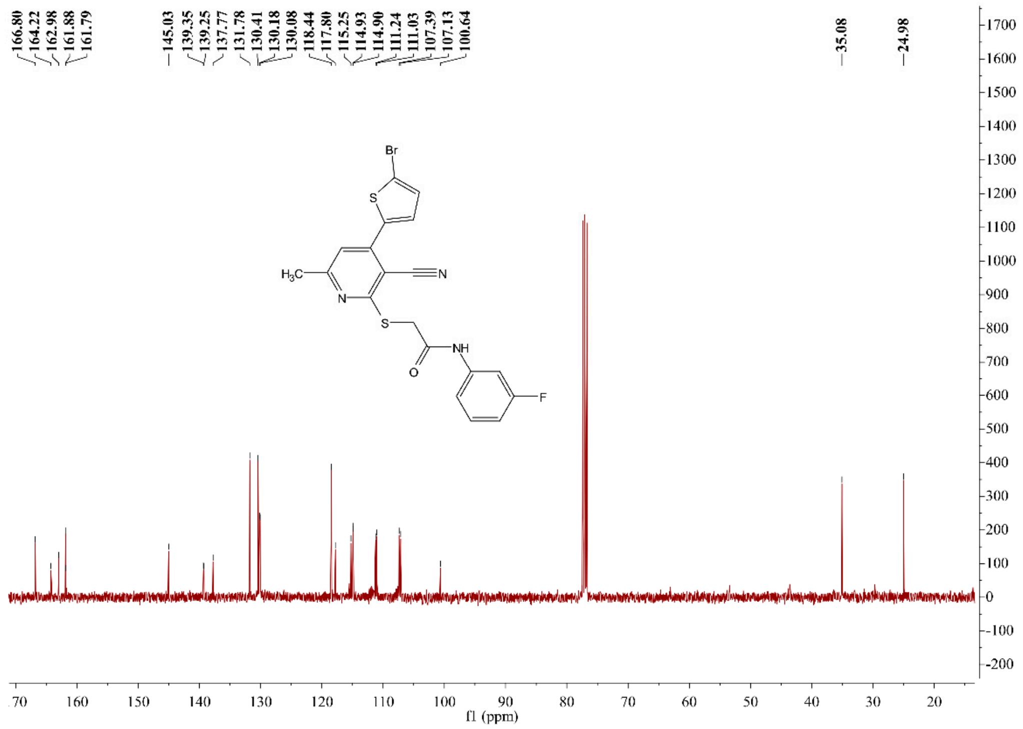

Figure S42. ^{13}C NMR spectrum of compound **Im**.

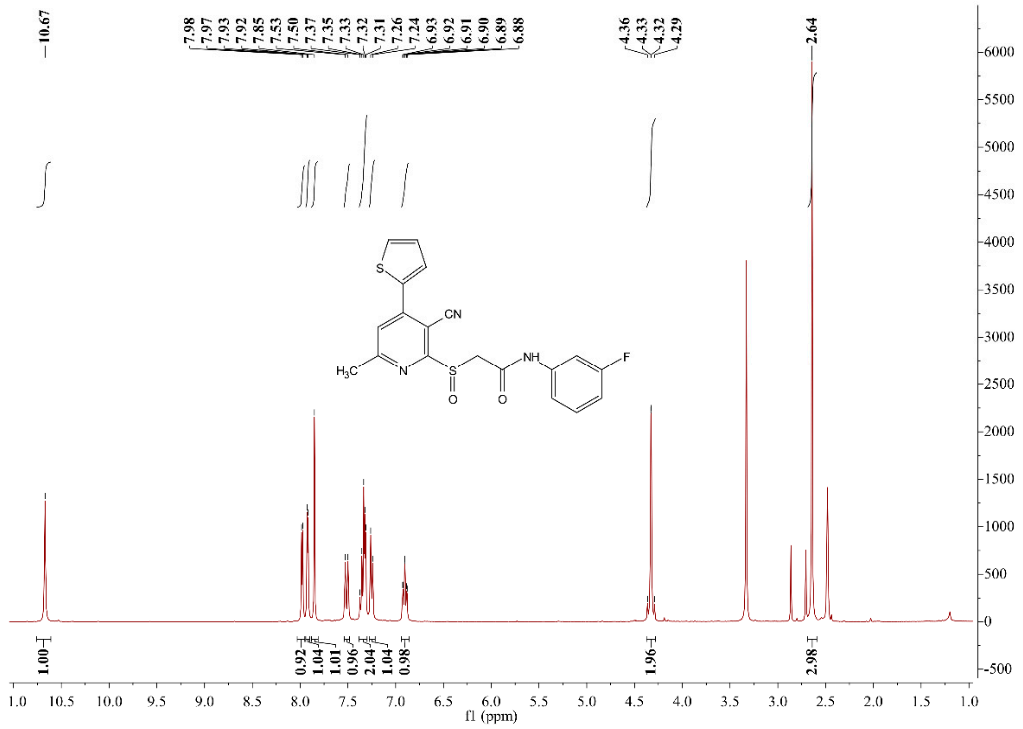

Figure S43. ^{1}H NMR spectrum of compound **In**.

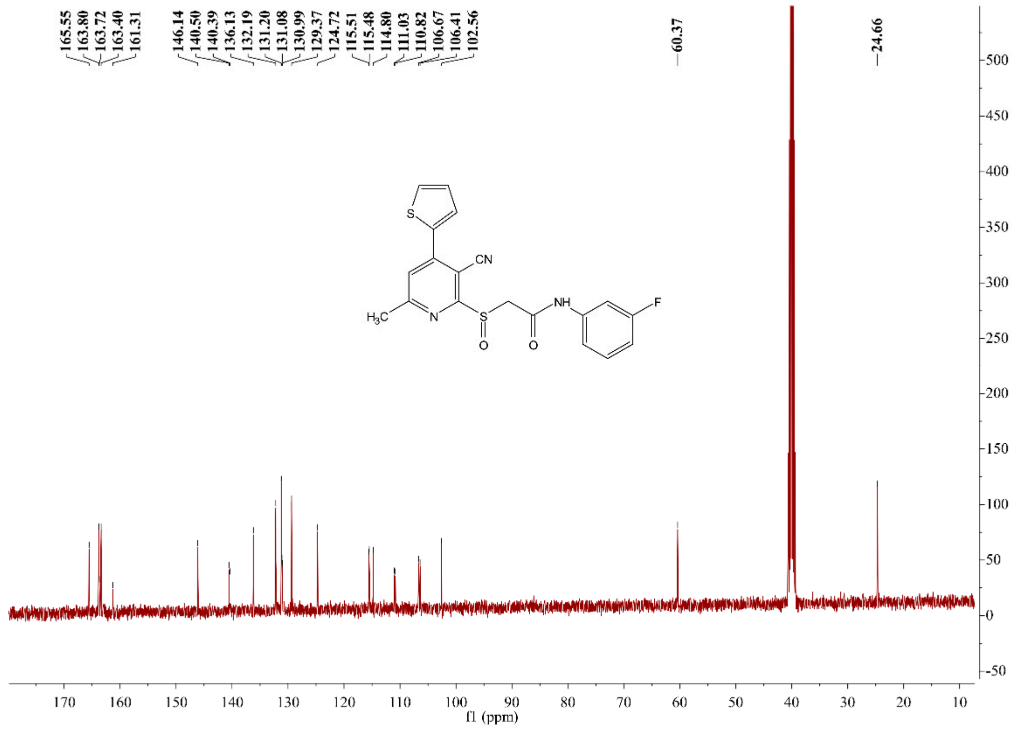

Figure S44. ^{13}C NMR spectrum of compound **In**.

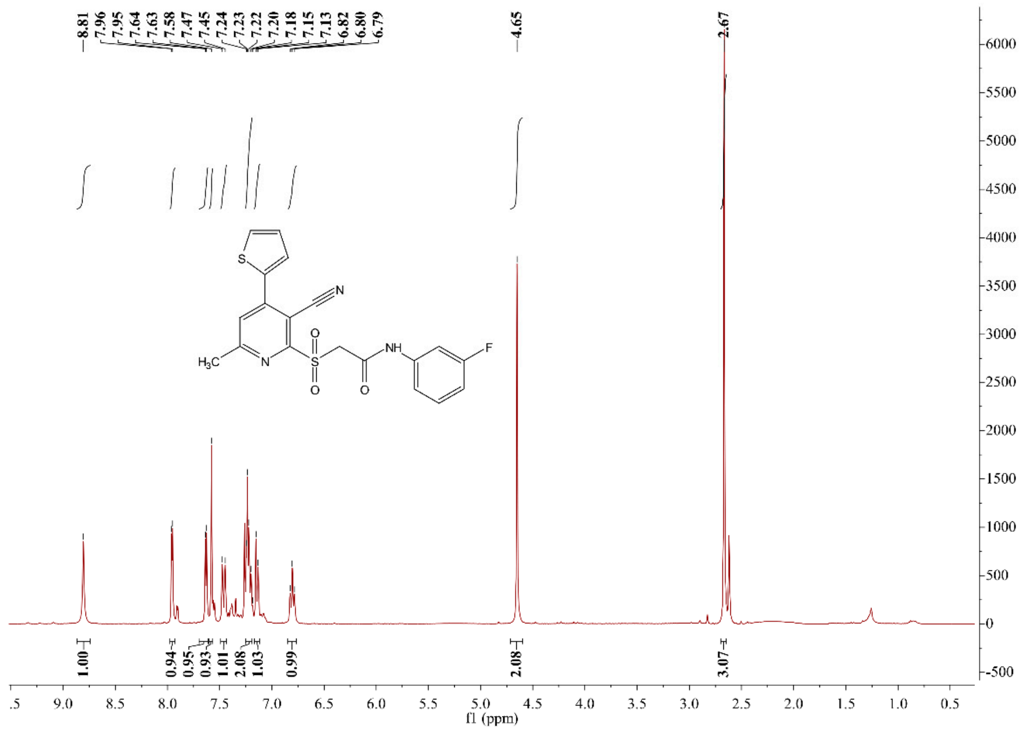

Figure S45. ^{1}H NMR spectrum of compound **Io**.

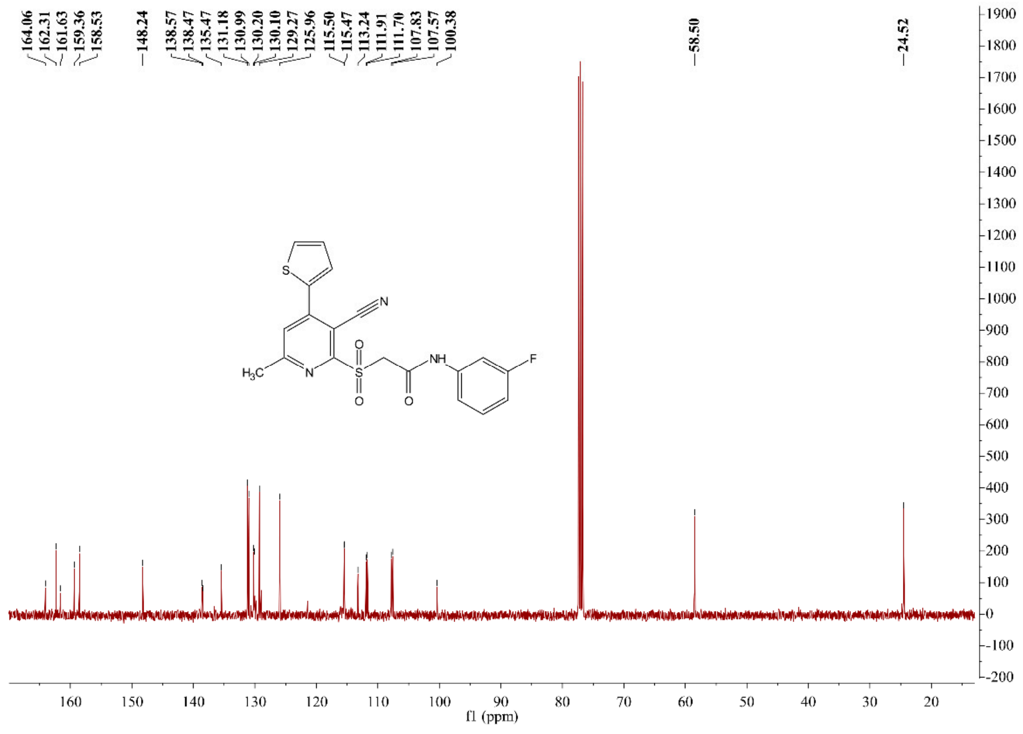

Figure S46. ^{13}C NMR spectrum of compound **Io**.

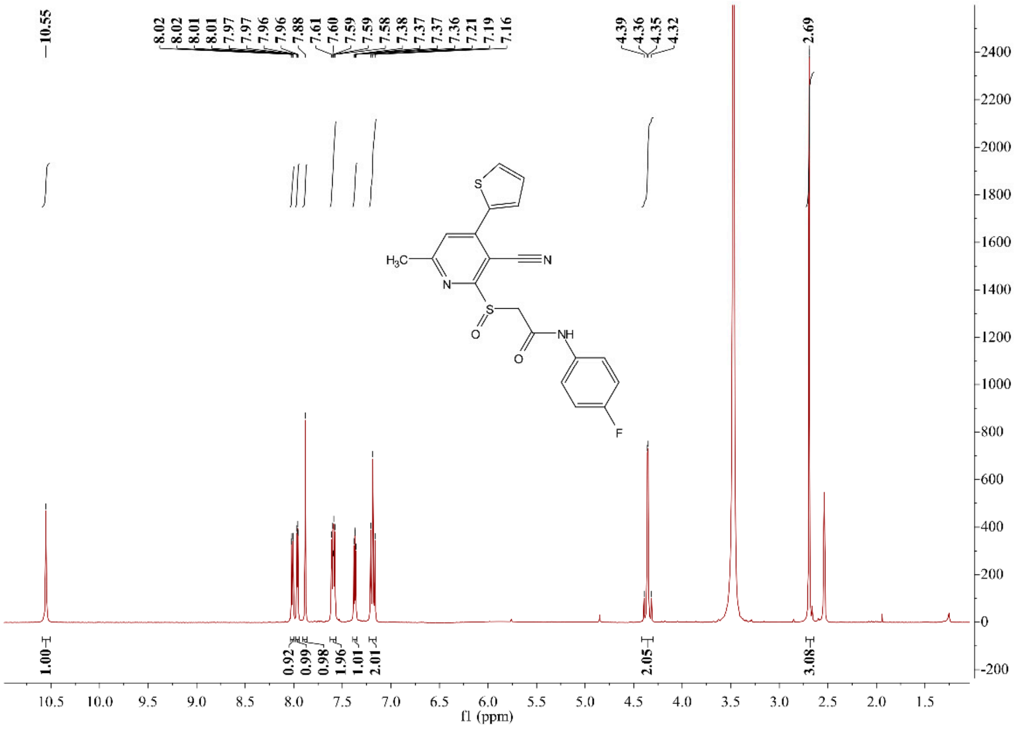

Figure S47. ^{1}H NMR spectrum of compound **Ip**.

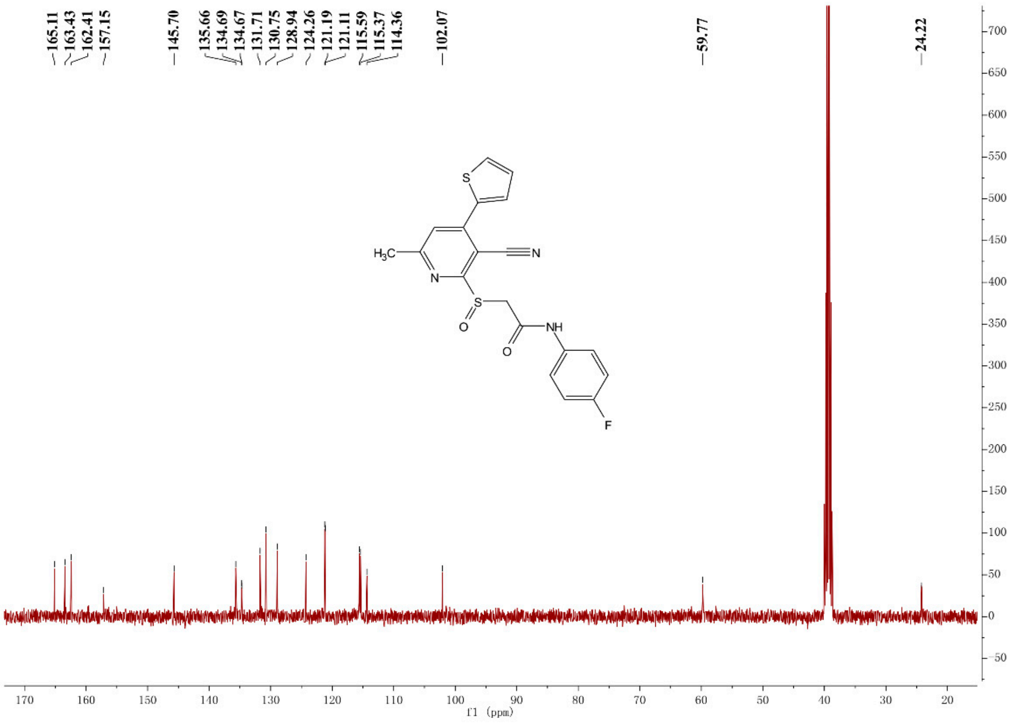

Figure S48. ^{13}C NMR spectrum of compound **Ip**.

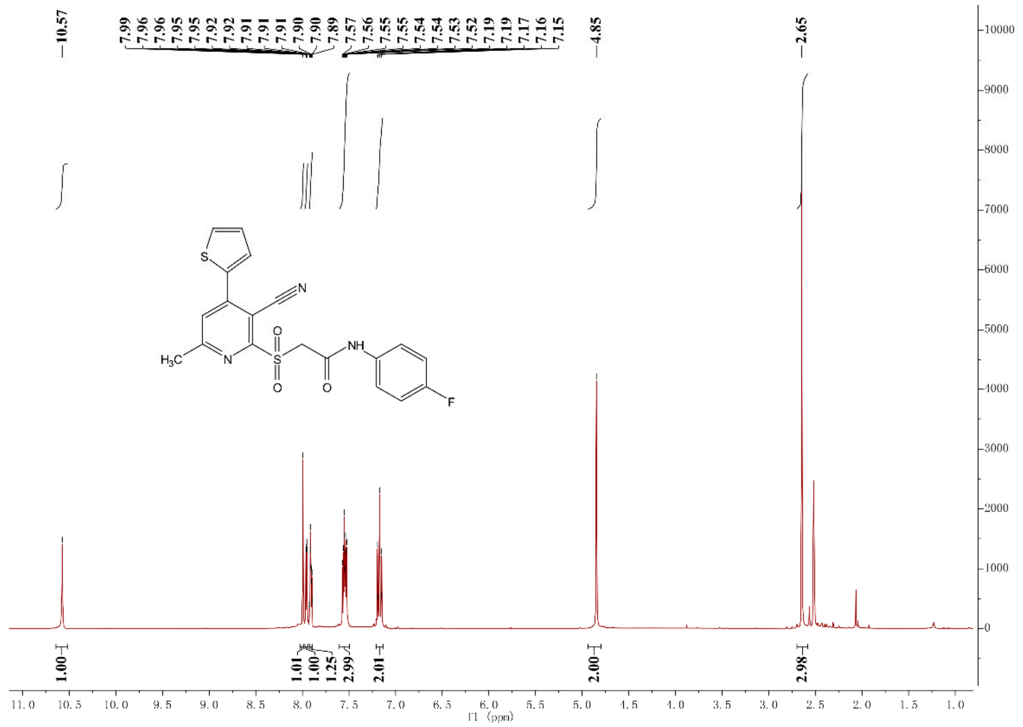

Figure S49. ^{1}H NMR spectrum of compound **Iq**.

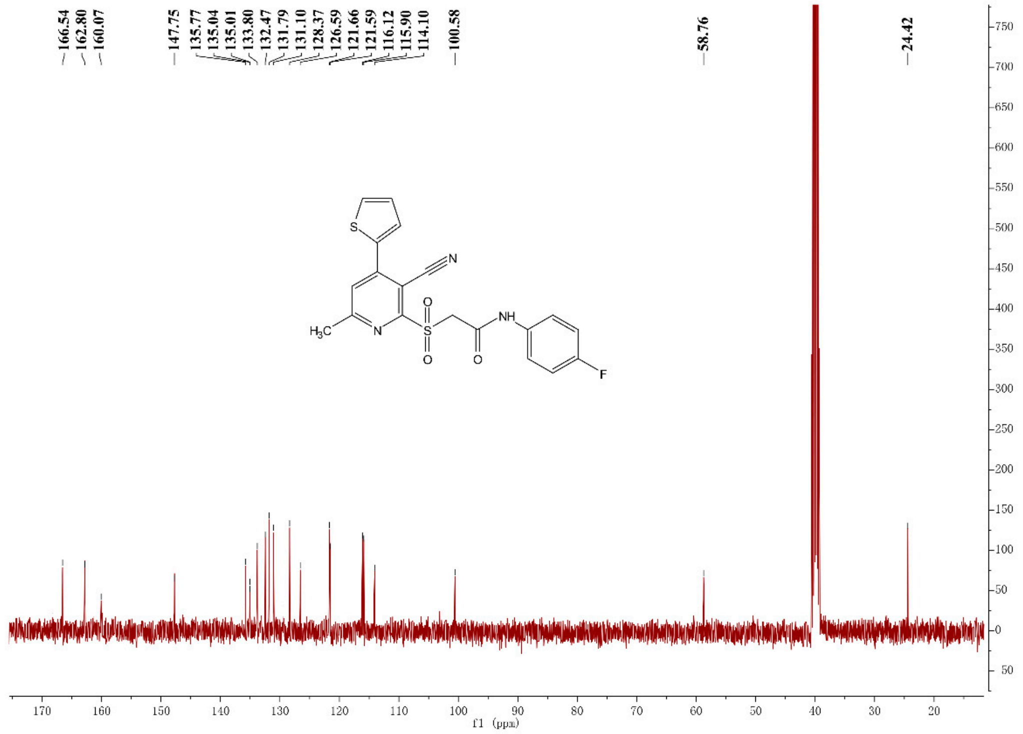

Figure S50. ^{13}C NMR spectrum of compound **Iq**.

2. The HRMS of compounds A and Ia-Iq

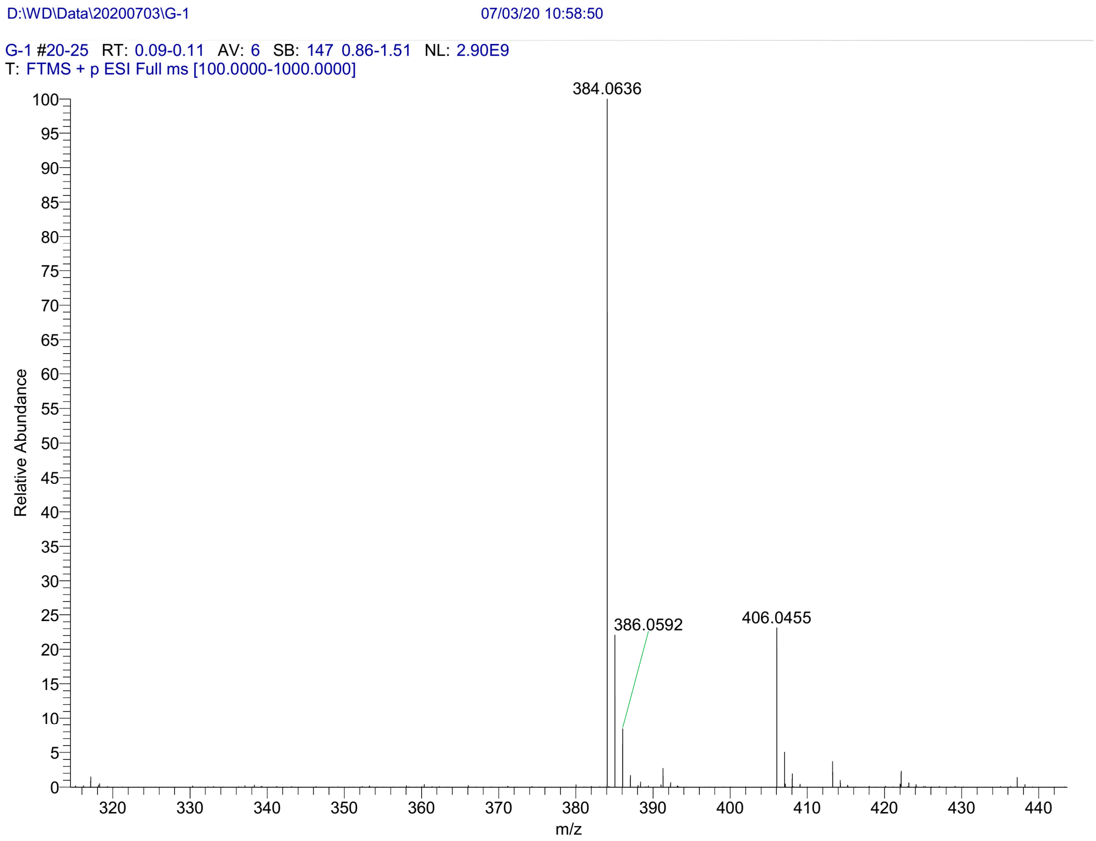

Figure S51. HRMS of compound **A**.

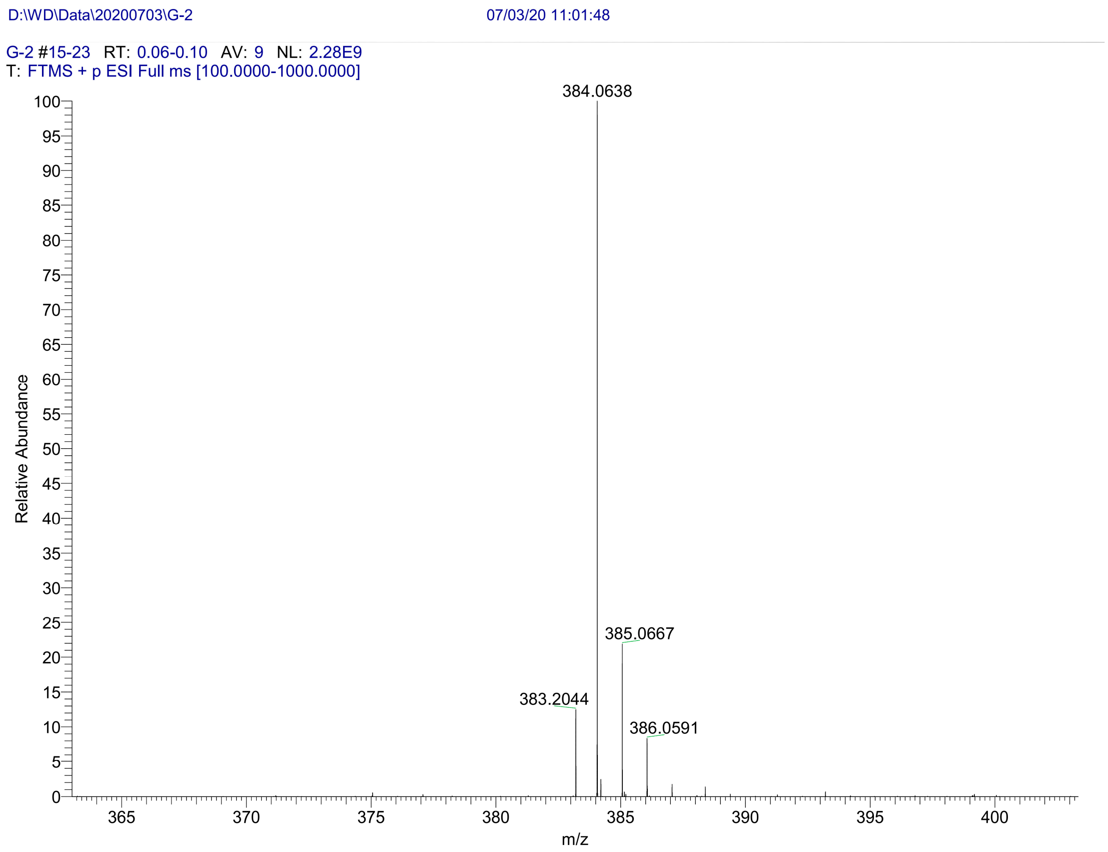

Figure S52. HRMS of compound **Ia**.

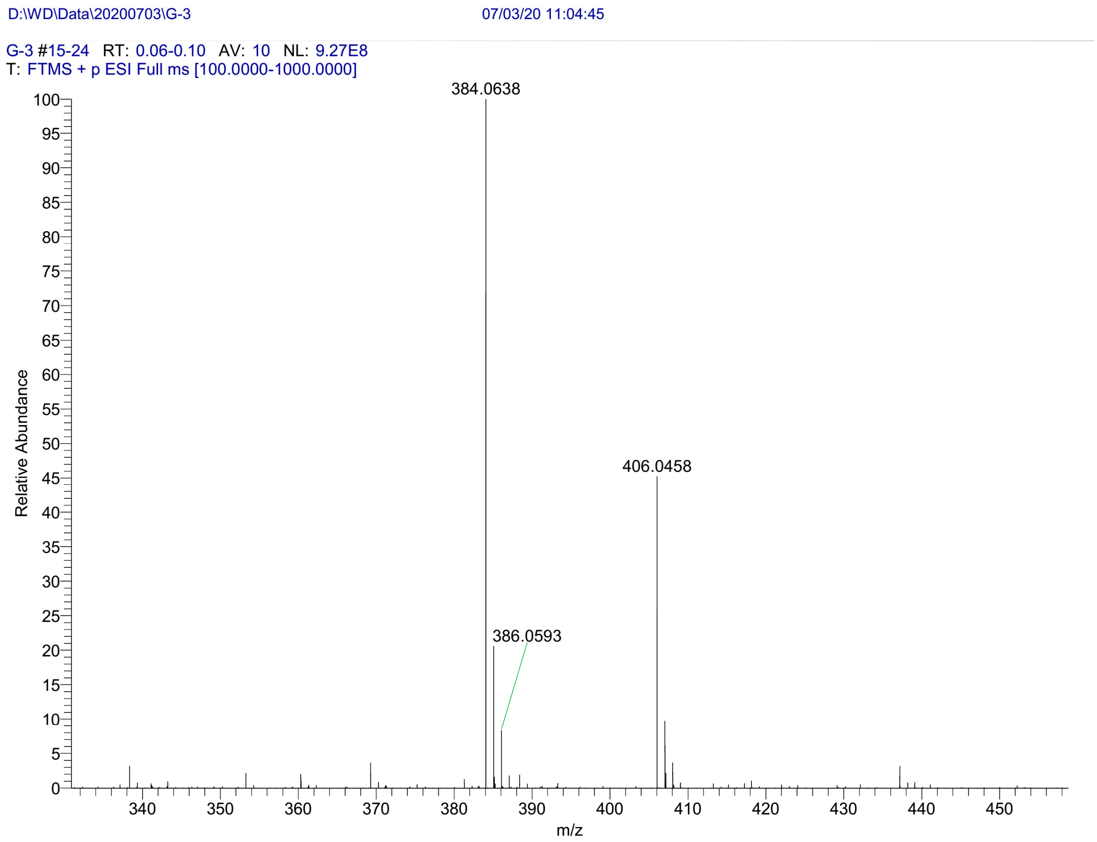

Figure S53. HRMS of compound **Ib**.

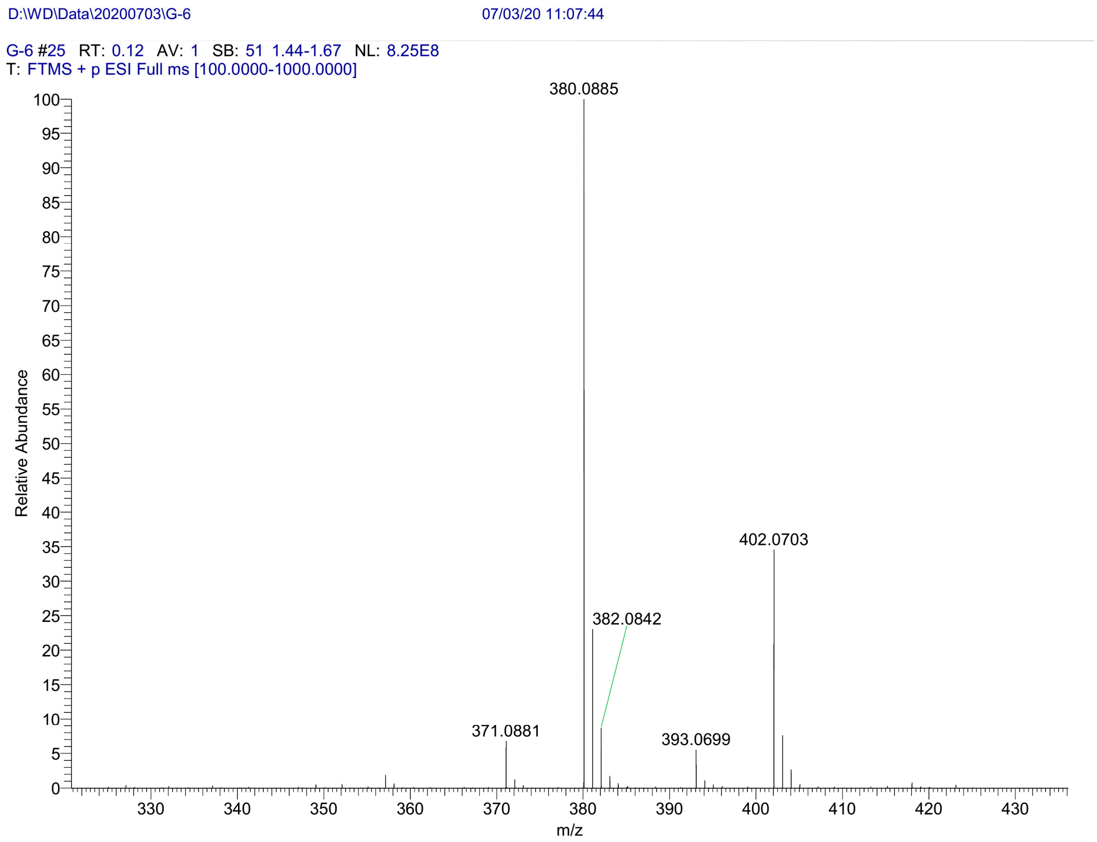

Figure S54. HRMS of compound **Ic**.

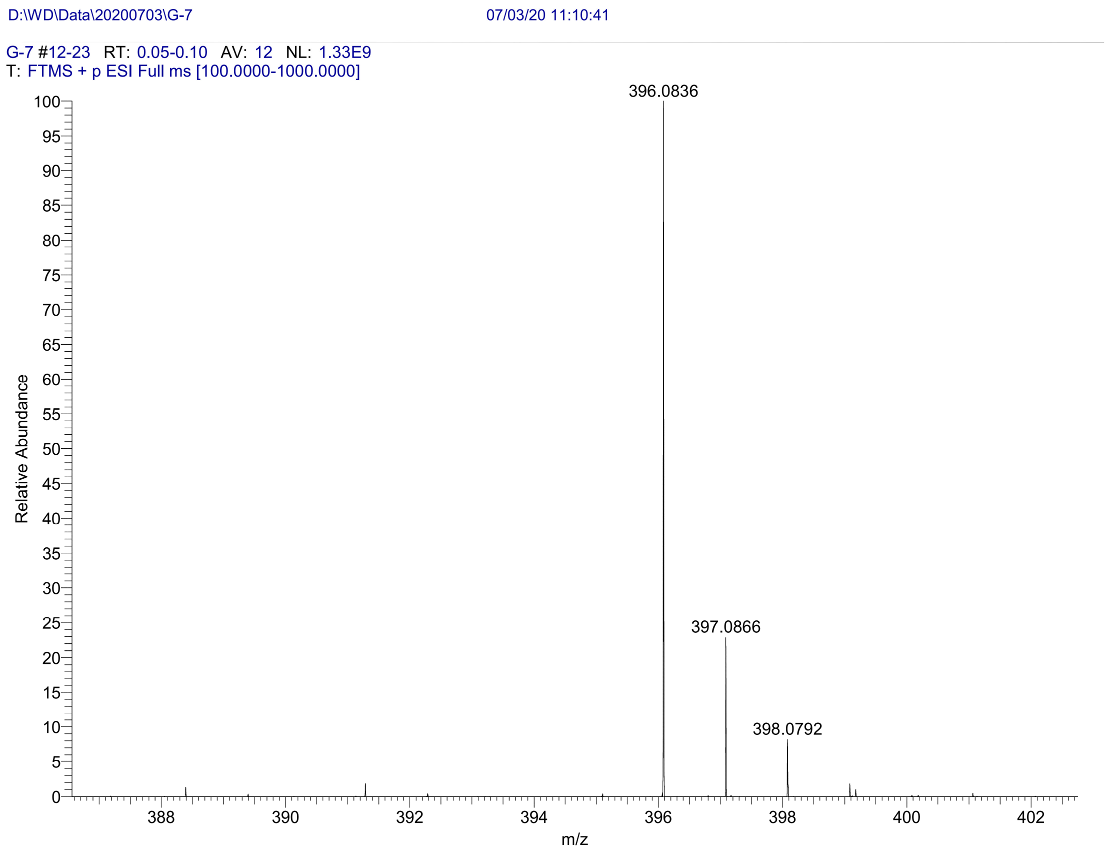

Figure S55. HRMS of compound **Id**.

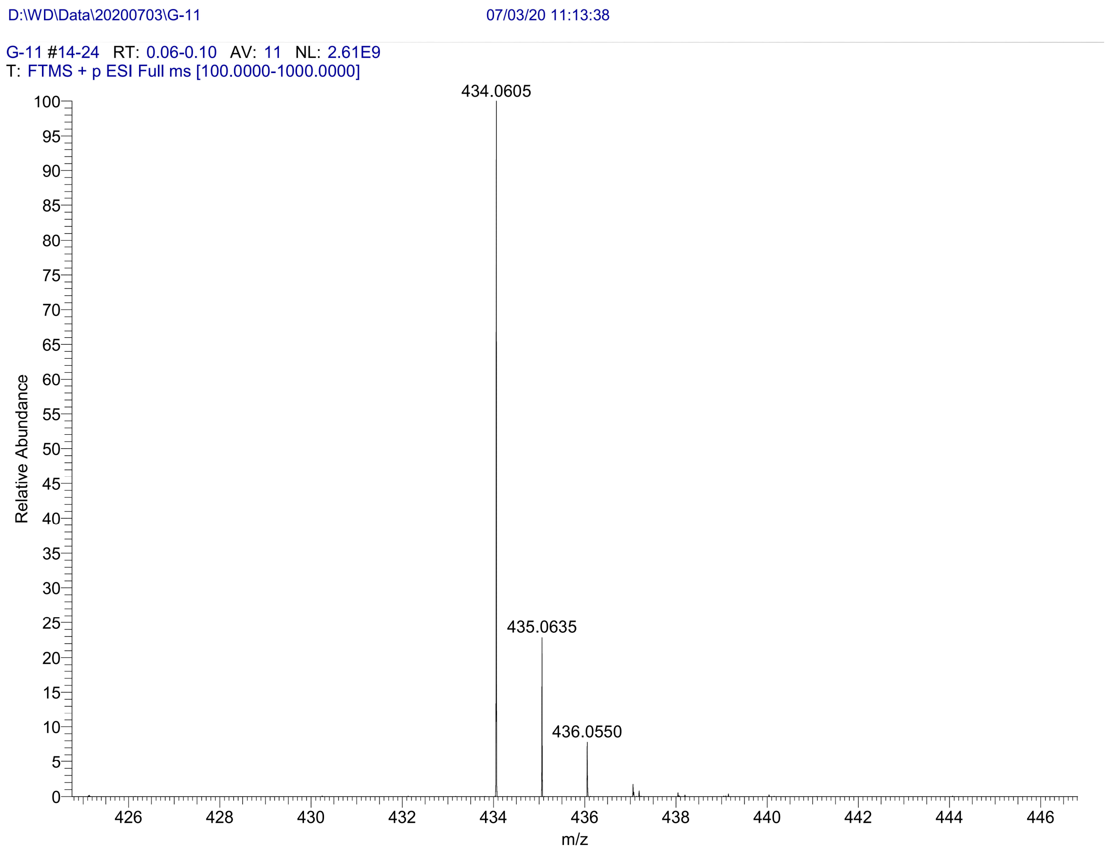

Figure S56. HRMS of compound **Ie**.

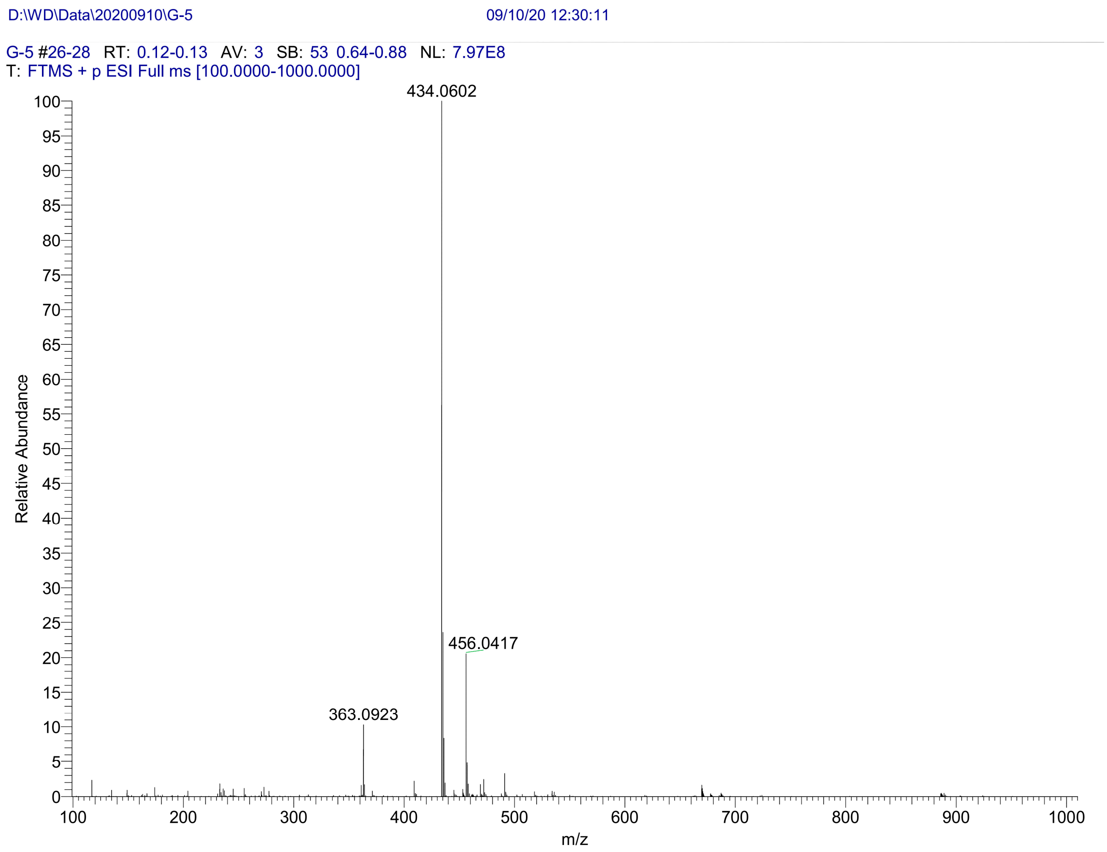

Figure S57. HRMS of compound **If**.

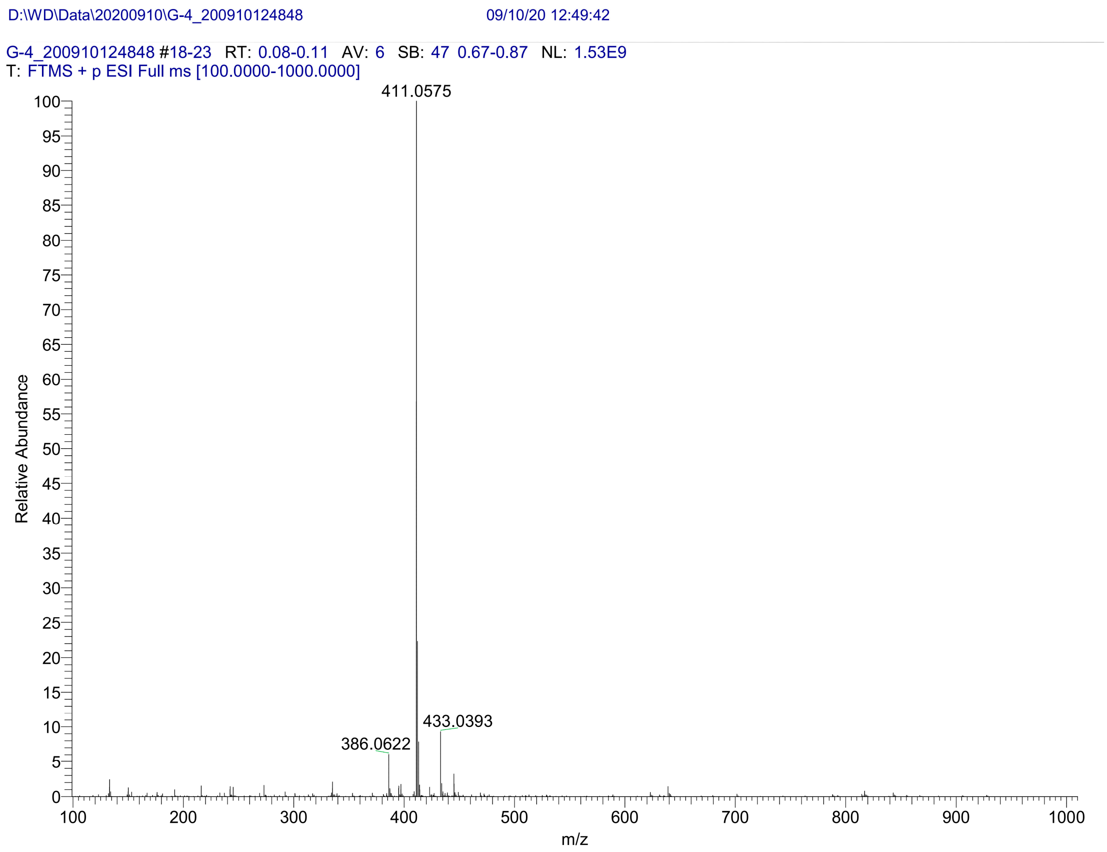

Figure S58. HRMS of compound **Ig**.

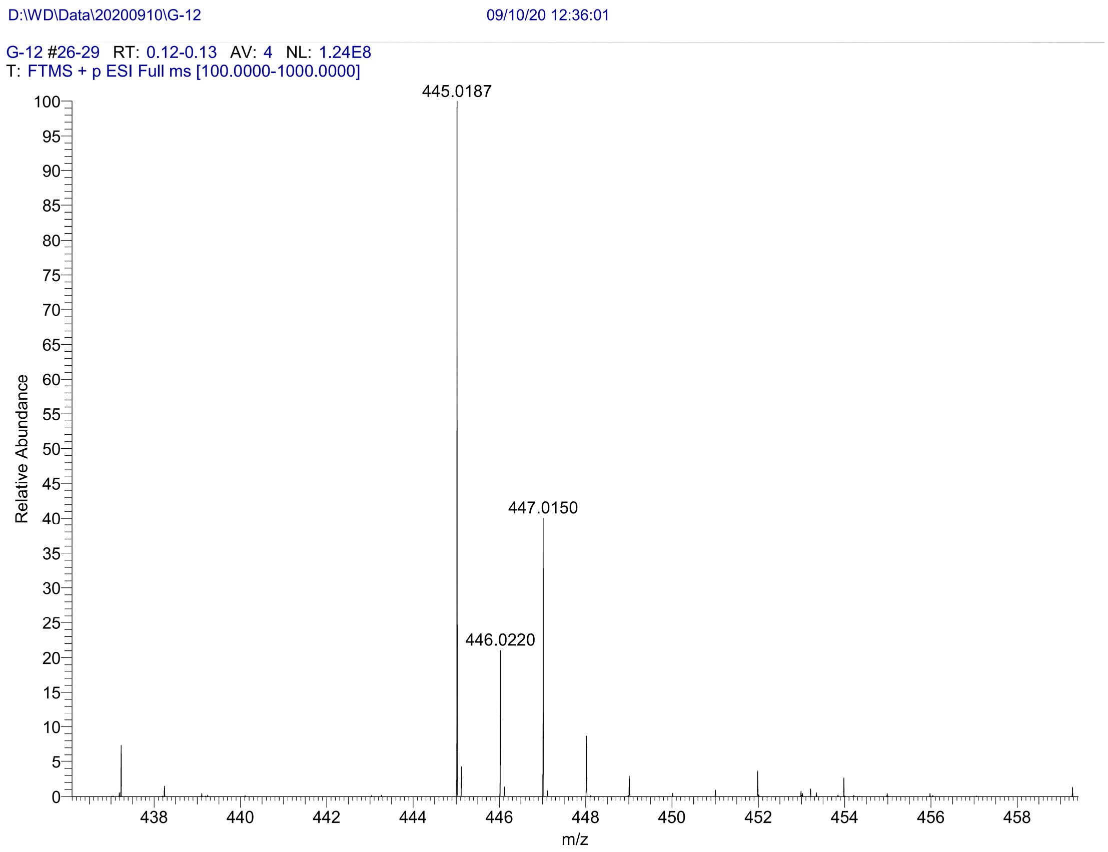

Figure S59. HRMS of compound **Ih**.

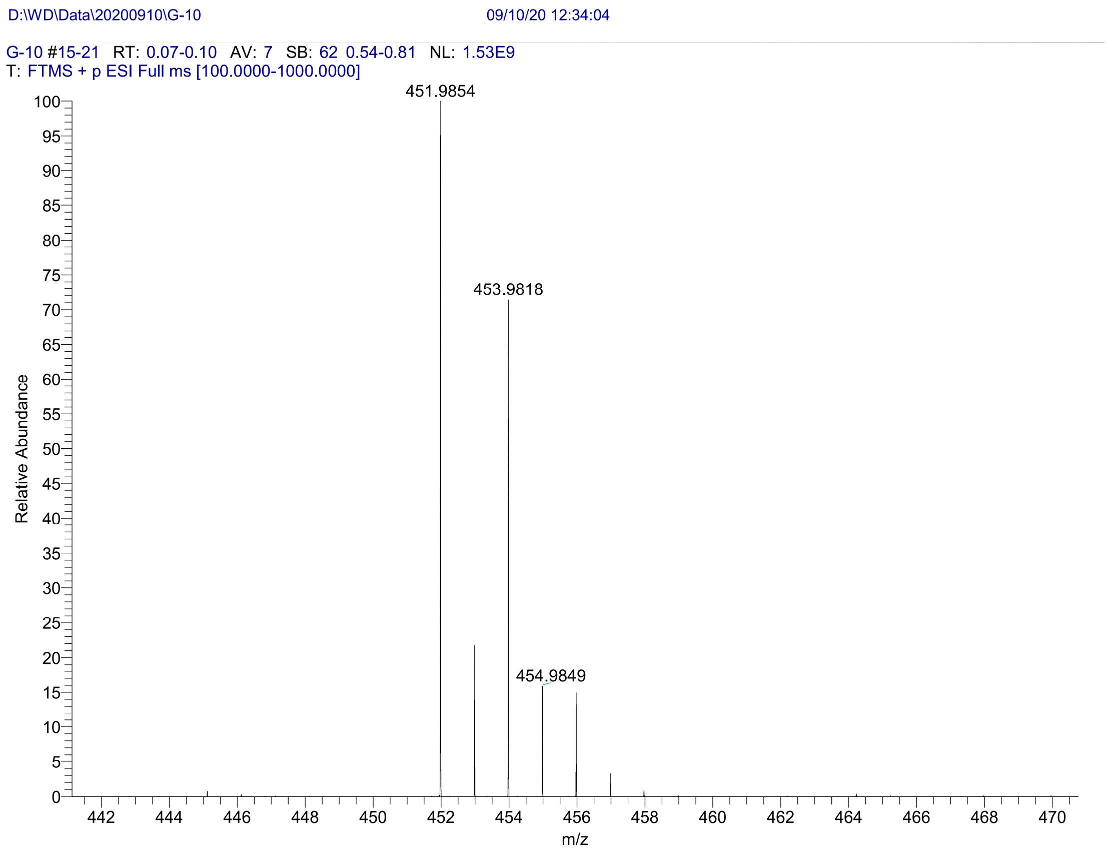

Figure S60. HRMS of compound **Ii**.

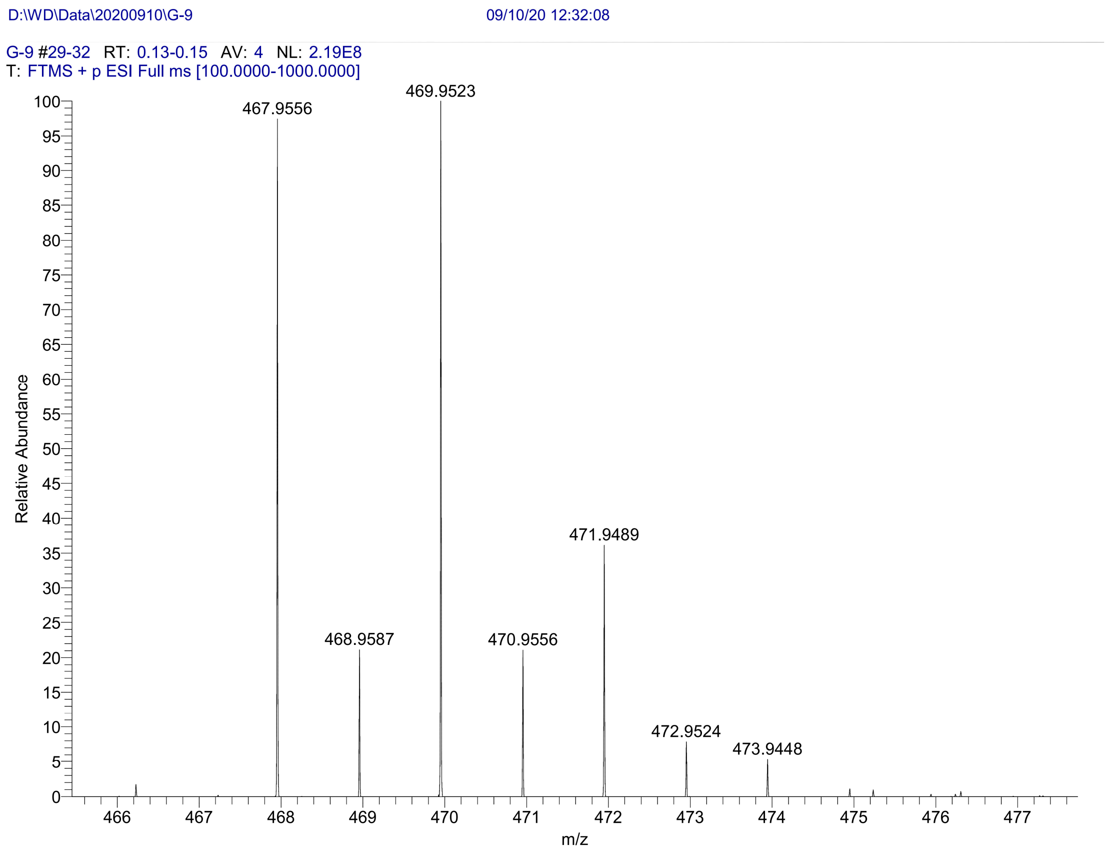

Figure S61. HRMS of compound **Ij**.

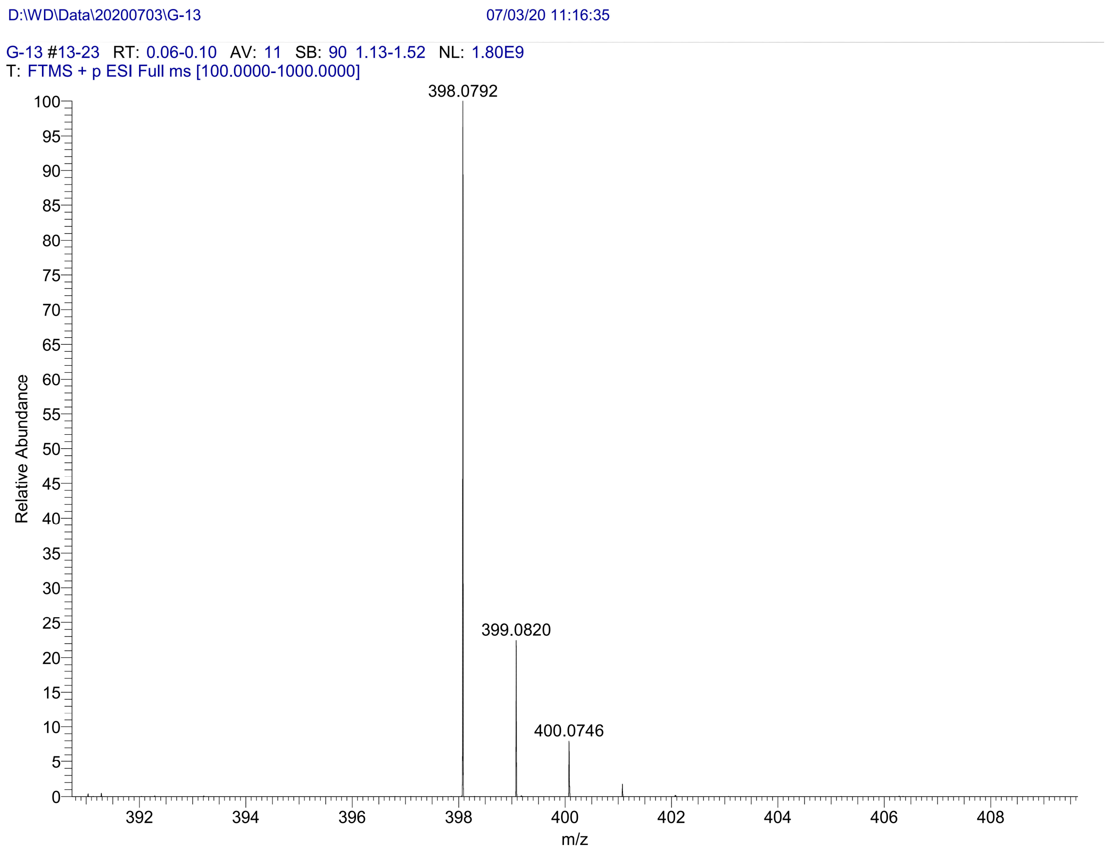

Figure S62. HRMS of compound **Ik**.

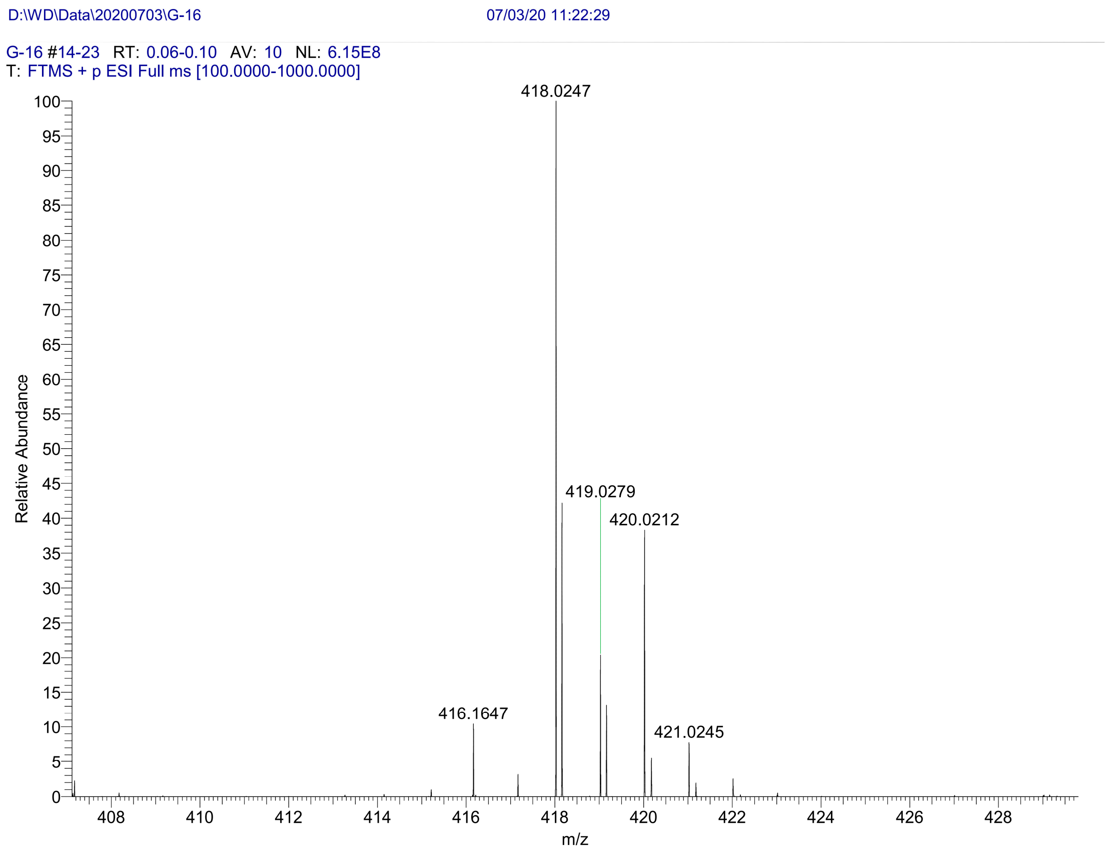

Figure S63. HRMS of compound **Il**.

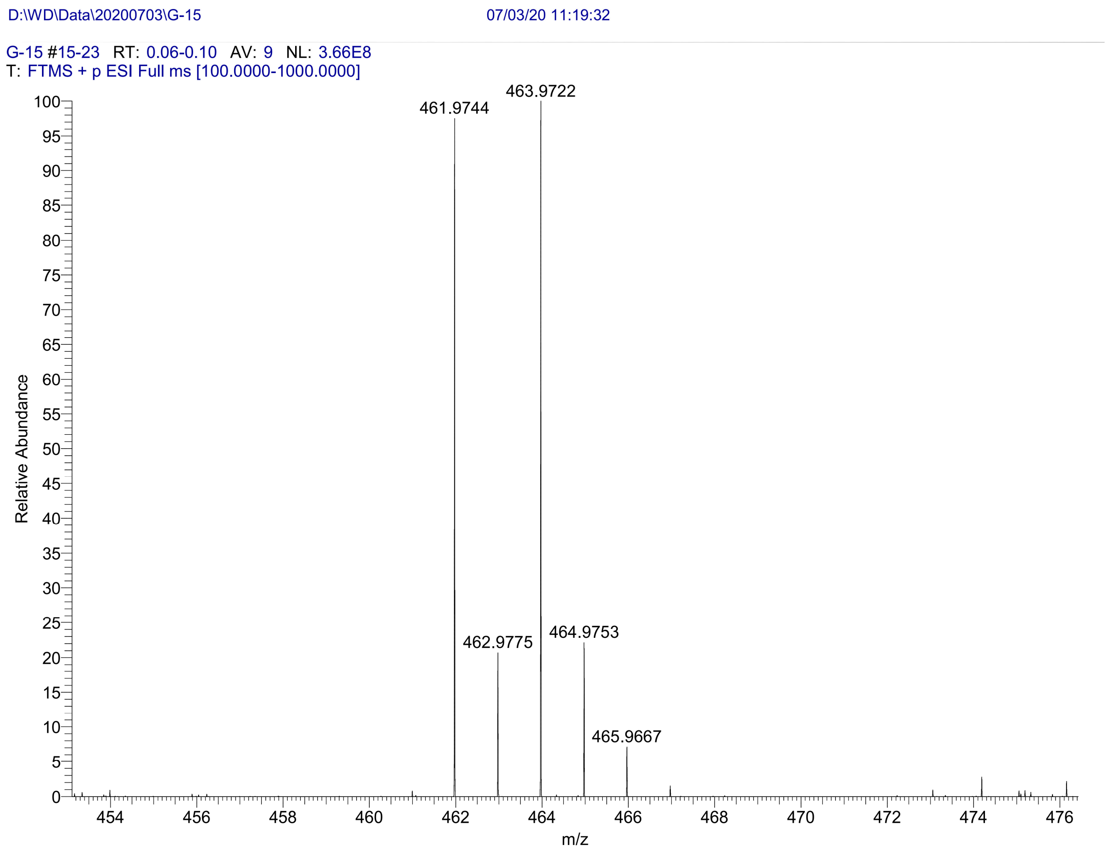

Figure S64. HRMS of compound **Im**.

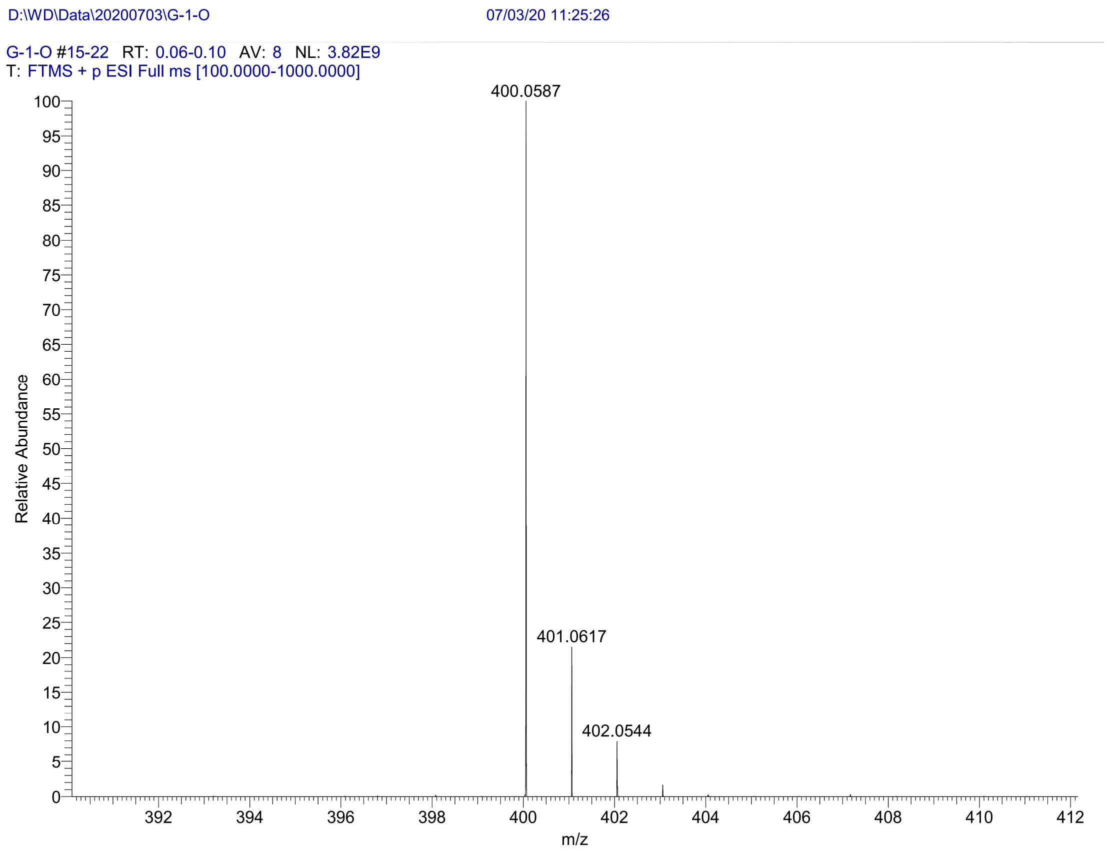

Figure S65. HRMS of compound **In**.

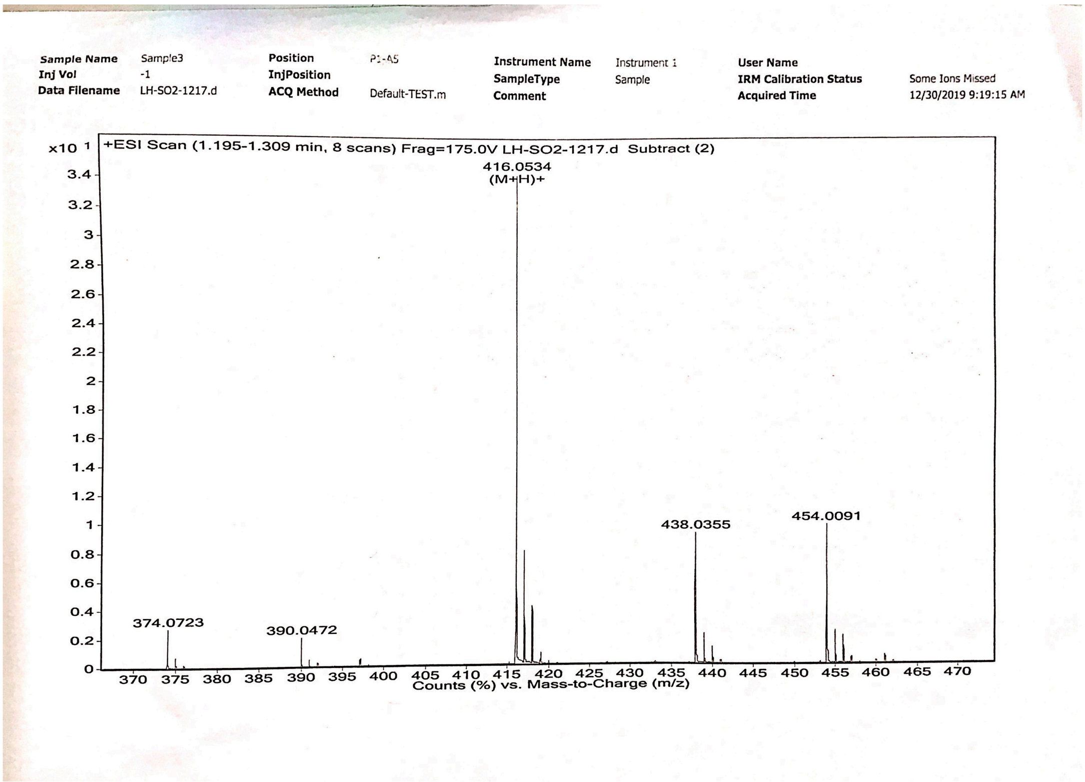

Figure S66. HRMS of compound **Io**.

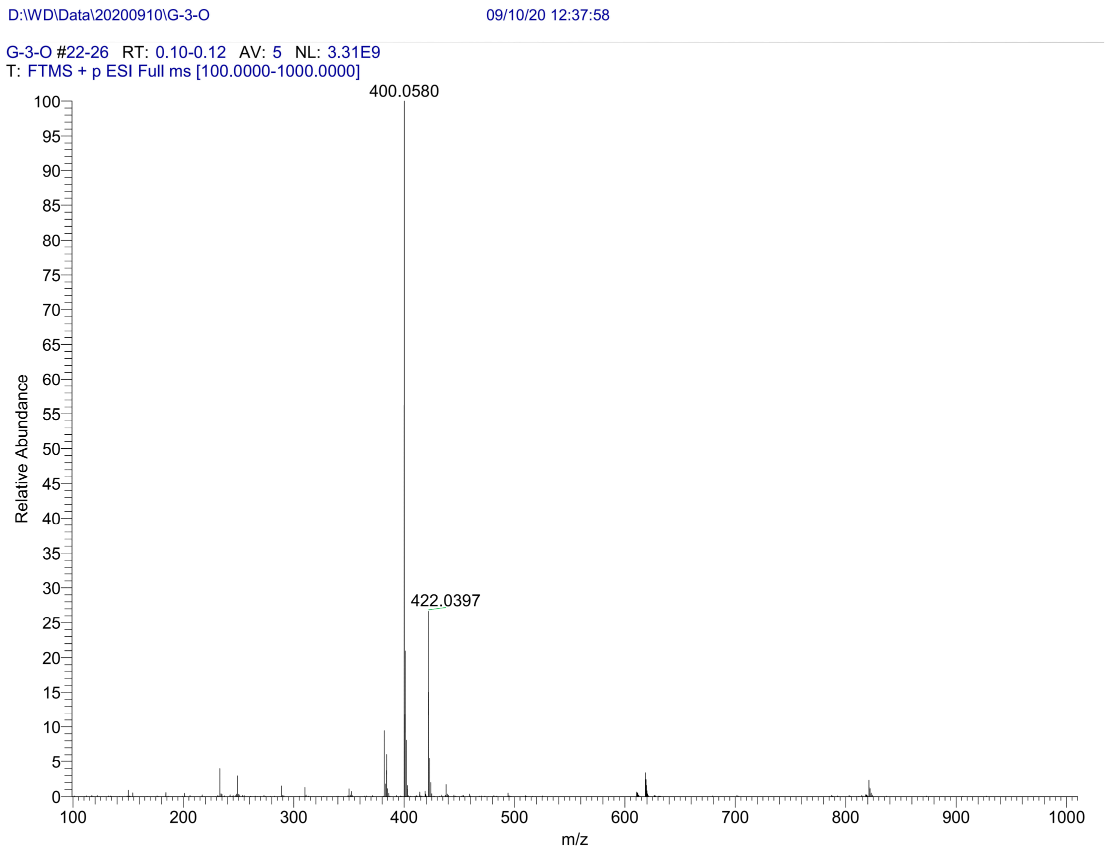

Figure S67. HRMS of compound **Ip**.

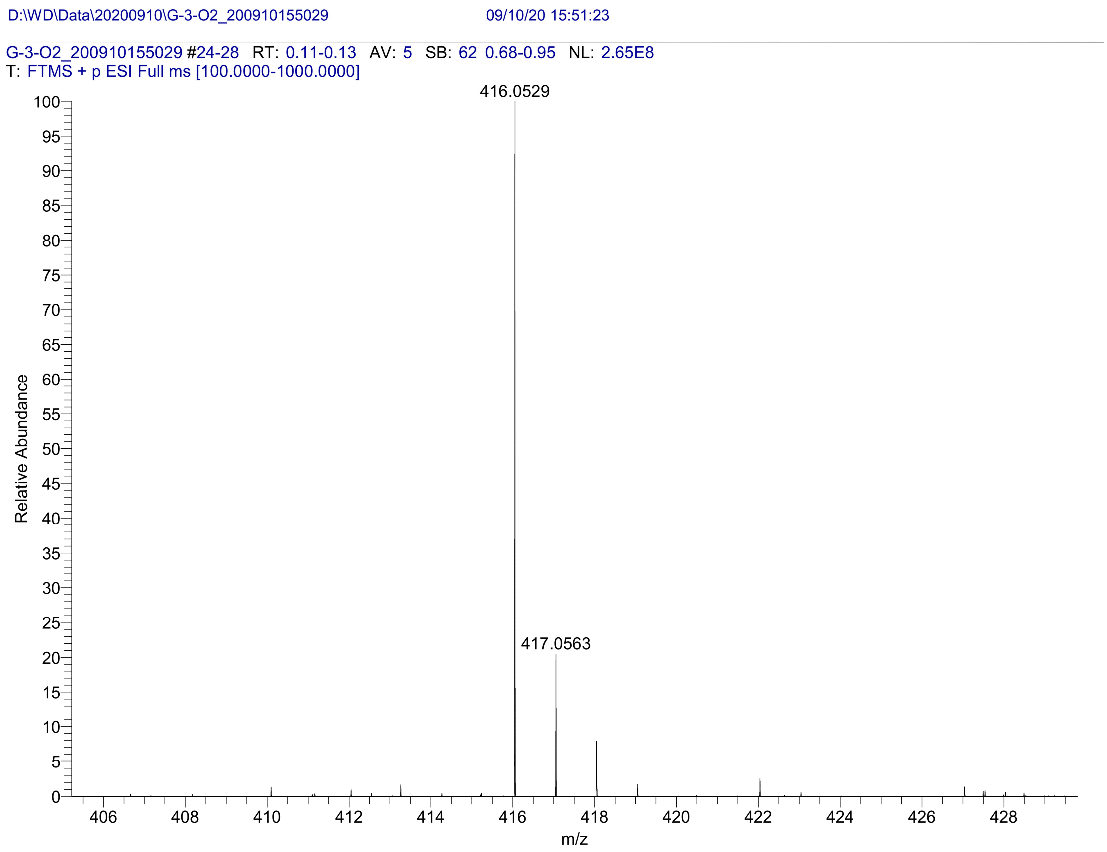

Figure S68. HRMS of compound **Iq**.

3. Crystal structure determination

Table S1. Crystal data and structure refinement for compound **A**.

Empirical formula	$C_{19}H_{14}FN_3OS_2$
Formula weight	383.45
Temperature/K	113.15
Crystal system, Space group	monoclinic, $P2_1/n$
Unit cell dimensions	a = 13.8820(5)Å α = 90 °.
	b = 8.7554(3)Å β = 100.698(4) °.
	c = 14.5268(6)Å γ = 90 °.
Volume	1734.93(11)$Å^3$
Z, Calculated density	4, 1.468 g/cm^3
Absorption coefficient	0.331 mm^{-1}
F(000)	792.0
Crystal size	0.2 × 0.18 × 0.16 mm^3
Radiation	Mo Kα (λ = 0.71073)
2Θ range for data collection	3.728 ° to 52.742 °
Limiting indices	$-17 \leq h \leq 17, -10 \leq k \leq 10, -18 \leq l \leq 16$
Reflections collected / unique	14637 / 3517 [R_{int} = 0.0423, R_{sigma} = 0.0323]
Data / restraints / parameters	3517 / 14 / 252
Goodness-of-fit on F^2	1.071
Final R indexes [I >= 2σ (I)]	R_1 = 0.0345, wR_2 = 0.0825
R indexes (all data)	R_1 = 0.0407, wR_2 = 0.0869
Largest diff. peak and hole	0.25 and -0.22 e·$Å^{-3}$

Table S2. Fractional atomic coordinates (×10^4) and equivalent isotropic displacement parameters (Å^2×10^3) for compound **A** (U(eq) is defined as one third of the trace of the orthogonalised Uij tensor).

Atom	*x*	*y*	*z*	U(eq)
S1	5545.2(6)	1200.8(11)	4092.3(8)	23.90(18)
C1	4999.8(19)	62(3)	3192.9(19)	24.6(5)
C2	4001.7(17)	169(4)	3059(2)	27.0(5)
C3	3670(5)	1194(13)	3713(7)	30.8(5)
C4	4418.2(17)	1871(8)	4307(4)	18.7(4)
S2	2525.2(3)	5415.1(5)	6257.6(3)	27.33(13)
F1	2850.8(8)	2899.9(13)	11272.9(7)	39.6(3)
O1	2217.7(9)	6054.8(14)	8560.8(9)	35.0(3)
N1	1894.6(10)	3469.5(18)	4145.7(10)	31.9(3)
N2	4446.0(9)	4777.3(14)	6692.0(9)	22.0(3)
N3	3710.0(9)	5006.7(15)	8487.3(9)	22.6(3)
C5	4425.8(11)	2919.3(16)	5090.4(10)	19.6(3)
C6	5289.9(11)	3237.9(17)	5732.6(11)	22.1(3)
C7	5276.9(11)	4147.3(17)	6505.1(11)	22.3(3)
C8	3623.0(11)	4549.6(17)	6074.9(10)	20.3(3)
C9	3573.6(11)	3648.3(17)	5263.2(10)	20.1(3)
C10	6196.2(12)	4474(2)	7200.3(12)	29.7(4)
C11	2643.4(11)	3528.3(18)	4639.8(11)	23.4(3)
C12	2949.1(13)	6667.8(18)	7243.3(11)	27.3(4)
C13	2914.7(12)	5892.3(17)	8169.0(11)	24.3(3)
C14	3878.8(11)	4045.5(17)	9278.1(10)	21.3(3)
C15	3260.2(12)	4002.8(18)	9930.1(11)	24.6(3)
C16	3486.8(12)	2983(2)	10661.6(11)	28.1(4)
C17	4286.3(13)	2040(2)	10800.2(12)	33.1(4)
C18	4903.6(13)	2116(2)	10150.8(12)	32.0(4)
C19	4699.6(12)	3099.9(18)	9393.4(11)	26.5(4)

Table S3. Bond lengths (Å) and angles (°) for compound **A**.

Atom-Atom	Dist. (Å)	Atom-Atom	Dist. (Å)
S1-C1	1.705(2)	N2-C7	1.351(2)
S1-C4	1.752(2)	N2-C8	1.3298(19)
C1-C2	1.366(3)	N3-C13	1.358(2)
C2-C3	1.443(7)	N3-C14	1.408(2)
C3-C4	1.357(5)	C5-C6	1.404(2)
C4-C5	1.461(3)	C5-C9	1.407(2)
C6-C7	1.379(2)	C7-C10	1.501(2)
C8-C9	1.410(2)	C9-C11	1.437(2)
C12-C13	1.515(2)	C14-C15	1.392(2)
S2-C8	1.7657(15)	C14-C19	1.393(2)
S2-C12	1.8129(17)	C15-C16	1.379(2)
F1-C16	1.3655(19)	C16-C17	1.368(3)
O1-C13	1.2186(19)	C17-C18	1.389(2)
N1-C11	1.149(2)	C18-C19	1.385(2)

Atom-Atom-Atom	angles(°)	Atom-Atom-Atom	angles(°)
C1-S1-C4	92.73(14)	N2-C7-C10	115.99(14)
C2-C1-S1	111.3(2)	C6-C7-C10	121.31(14)
C1-C2-C3	112.9(4)	N2-C8-S2	119.46(12)
C4-C3-C2	112.9(6)	N2-C8-C9	123.37(14)
C3-C4-S1	110.2(4)	C9-C8-S2	117.17(11)
C3-C4-C5	131.5(4)	C5-C9-C8	119.41(13)
C5-C4-S1	118.11(15)	C5-C9-C11	122.78(14)
C8-C9-C11	117.81(14)	N1-C11-C9	178.30(18)
C13-C12-S2	111.77(11)	O1-C13-N3	124.99(15)
O1-C13-C12	121.39(15)	N3-C13-C12	113.61(14)
C15-C14-N3	122.74(14)	C8-S2-C12	102.46(8)
C15-C14-C19	119.76(15)	C8-N2-C7	117.52(13)
C19-C14-N3	117.50(14)	C13-N3-C14	128.36(14)
C16-C15-C14	117.42(15)	C6-C5-C4	121.63(18)
F1-C16-C15	116.96(15)	F1-C16-C17	118.48(15)
C6-C5-C9	115.73(14)	C17-C16-C15	124.55(16)
C9-C5-C4	122.63(17)	C16-C17-C18	117.20(16)
C19-C18-C17	120.58(16)	C7-C6-C5	121.12(14)
C18-C19-C14	120.48(16)	N2-C7-C6	122.69(14)

Table S4. Torsion angles (º) for compound **A**.

A-B-C-D	Angle/°	A-B-C-D	Angle/°
S1-C1-C2-C3	-0.6(7)	S2-C12-C13-O1	-95.57(16)
S1-C4-C5-C6	-8.1(7)	S2-C12-C13-N3	83.20(15)
S1-C4-C5-C9	173.1(3)	F1-C16-C17-C18	178.04(14)
C1-S1-C4-C3	1.5(8)	N2-C8-C9-C5	1.1(2)
C1-S1-C4-C5	177.3(5)	N2-C8-C9-C11	-178.33(14)
C1-C2-C3-C4	1.8(12)	N3-C14-C15-C16	178.21(14)
C2-C3-C4-S1	-2.1(12)	N3-C14-C19-C18	-179.40(14)
C2-C3-C4-C5	-177.2(7)	C5-C6-C7-N2	0.1(2)
C3-C4-C5-C6	166.6(10)	C5-C6-C7-C10	179.17(14)
C3-C4-C5-C9	-12.1(13)	C6-C5-C9-C8	-3.8(2)
C4-S1-C1-C2	-0.4(4)	C6-C5-C9-C11	175.65(14)
C4-C5-C6-C7	-175.6(4)	C7-N2-C8-S2	-177.26(11)
C4-C5-C9-C8	175.0(4)	C7-N2-C8-C9	2.3(2)
C4-C5-C9-C11	-5.6(4)	C8-S2-C12-C13	-91.98(12)
C8-N2-C7-C6	-3.0(2)	C8-N2-C7-C10	177.97(13)
C9-C5-C6-C7	3.2(2)	C12-S2-C8-N2	8.19(14)
C12-S2-C8-C9	-171.40(12)	C13-N3-C14-C15	-7.5(2)
C13-N3-C14-C19	171.96(15)	C14-N3-C13-O1	4.0(3)
C14-N3-C13-C12	-174.75(14)	C14-C15-C16-F1	-177.05(13)
C14-C15-C16-C17	1.5(2)	C15-C14-C19-C18	0.1(2)
C15-C16-C17-C18	-0.5(3)	C16-C17-C18-C19	-0.7(3)
S2-C8-C9-C5	179.29(11)	C17-C18-C19-C14	0.9(2)
S2-C8-C9-C11	1.24(18)	C19-C14-C15-C16	-1.3(2)
